# Application and Challenges of Chimeric Antigen Receptor T Cell Therapy in Systemic Rheumatic Diseases and Autoimmune Disorders

**DOI:** 10.1002/mco2.70658

**Published:** 2026-03-16

**Authors:** Zhidan Fan, Li Zhang, Haiguo Yu

**Affiliations:** ^1^ Department of Rheumatology and Immunology Children's Hospital of Nanjing Medical University Nanjing China; ^2^ Department of Rehabilitation Children's Hospital of Nanjing Medical University Nanjing China

**Keywords:** autoimmune disorders (AIDs), biomarker discovery, CAR‐T cell therapy, immune reconstitution, in vivo CAR‐T, multiomics integration, precision immunology, universal CAR‐T

## Abstract

Chimeric antigen receptor T (CAR‐T) cell therapy, originally developed for hematologic malignancies, has emerged as a transformative candidate for systemic rheumatic diseases and autoimmune disorders (AIDs). Its unique efficacy in refractory AIDs relies on depleting autoreactive B cells and driving antigen‐naïve immune reconstitution, achieving durable drug‐free remission in early‐phase trials. Despite promising clinical and serological responses lasting 2–5 years without long‐term immunosuppression, the field faces unmet needs: complex manufacturing, limited tissue penetration, antigen escape, immunological sequelae, and lack of predictive biomarkers. Existing reviews predominantly focus on oncology adaptations or isolated technical aspects, lacking systematic integration of mechanisms, challenges, and precision‐oriented innovations for rheumatic diseases. This review comprehensively summarizes CAR‐T's action mechanisms in AIDs, analyzes core clinical challenges, and highlights emerging strategies—including universal/in vivo‐generated CAR‐T cells, multitargeted/logic‐gated designs, organ‐homing engineering, and rational combinations with tolerance‐enhancing agents. It further emphasizes multiomics integration (single‐cell transcriptomics, spatial mapping, B‐cell receptor/T‐cell receptor repertoire analysis) for patient stratification and relapse prediction. By bridging mechanism‐driven engineering with clinical translation, this work provides an actionable framework to advance CAR‐T toward functional immune reset, enabling precision immunotherapy for refractory rheumatic diseases and AIDs.

## Graphical abstract

CAR‐T cell therapy in systemic rheumatic diseases and autoimmune disorders. (This figure was created using BioRender.com.)

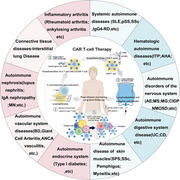

## 1 Introduction

**FIGURE 1:**
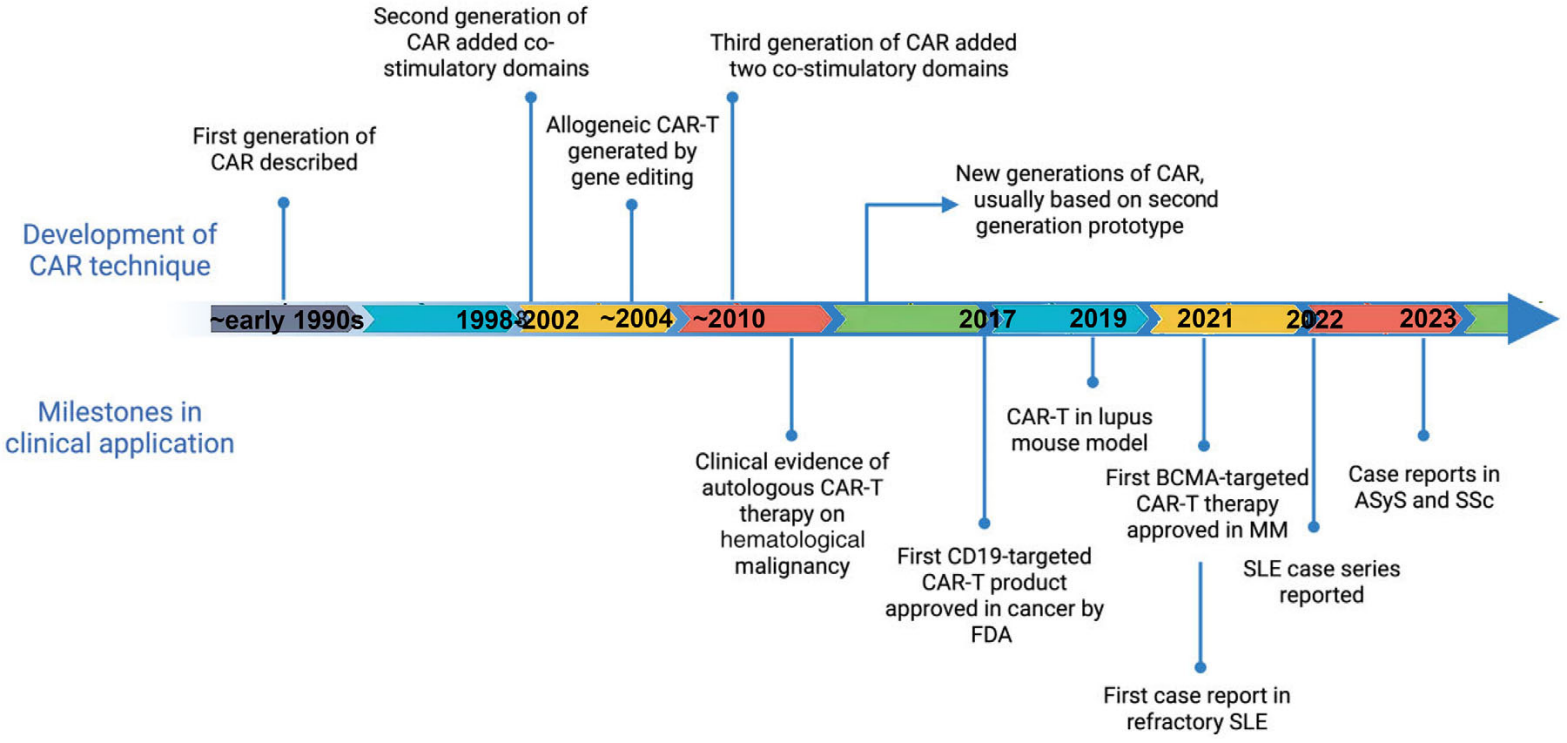
Evolution of chimeric antigen receptor (CAR) immunocyte therapy in the management of AIDs and rheumatic immune disorders. Initially developed for the targeted eradication of tumor cells, CAR‐T therapy reached a significant milestone in 2017 with the regulatory approval of a CD19‐targeted CAR‐T product for the treatment of hematologic malignancies. In 2021, a landmark achievement was reported with the successful application of CD19‐targeted CAR‐T therapy in a case of refractory systemic lupus erythematosus. From 2022 onward, there has been an increasing volume of clinical literature documenting the expanding use of CAR‐T therapy in various autoimmune and rheumatic immune conditions. (This figure was created using BioRender.com.)

Systemic rheumatic diseases (SRDs) and autoimmune disorders (AIDs)—encompassing rheumatoid arthritis (RA), systemic lupus erythematosus (SLE), Sjögren's syndrome (SS), and systemic sclerosis (SSc)—affect approximately 10% of the global population, leading to chronic tissue damage and impaired quality of life [1, 2]. Clinically, AIDs are classified into organ‐specific subtypes (e.g., myasthenia gravis [MG] targeting the nervous system, type 1 diabetes (T1D) damaging pancreatic islet cells, and autoimmune hepatitis involving hepatic tissues) [3, 4] and systemic forms characterized by multiorgan involvement and immune dysregulation, such as SLE, RA, and SSc [5, 6]. Key unmet therapeutic needs persist, including the inability to eliminate long‐lived plasma cells (LLPCs)—reservoirs of pathogenic autoantibodies—high relapse rates following treatment discontinuation, and infections associated with nonspecific systemic immunosuppression [7]. Autologous hematopoietic stem cell transplantation (HSCT) can induce durable remission but is limited to severe cases due to its high toxicity [8, 9], prompting the exploration of precision therapies like chimeric antigen receptor T (CAR‐T) cell therapy for restoring immune tolerance in AIDs [10] (Figure 1).

CAR‐T cell therapy integrates antibody specificity with T cell cytotoxicity to selectively eliminate pathogenic cells in an major histocompatibility complex (MHC)‐unrestricted manner, involving genetic engineering of T cells to express CARs, in vitro purification, expansion, activation, and patient reinfusion. Initially developed for B‐cell hematologic malignancies, its success in depleting CD19+ cells spurred repurposing for AIDs—driven by the recognition that autoreactive B cells and plasma cells (key pathogenic drivers of diseases like SLE and neuromyelitis optica spectrum disorder [NMOSD]) express CD19/B cell maturation antigen (BCMA) antigens targeted in oncology [11]. Unlike oncological applications focused on tumor eradication, CAR‐T therapy for AIDs aims to induce immune reset via naïve B‐cell reconstitution, addressing the root cause of AIDs (immune tolerance loss) rather than merely alleviating symptoms [12, 13]. As a novel therapeutic modality for non‐neoplastic diseases, CAR‐T has shown promising preliminary efficacy and tolerability in AIDs, with applications reported in SLE, antisynthetase syndrome (ASS), and SSc [5, 14, 15, 16, 17]. However, the rapid pace of clinical research, emerging advancements, and unresolved questions regarding long‐term efficacy, safety profiles, and optimal therapeutic strategies across different AIDs have not been systematically synthesized in existing literature.

This review comprehensively summarizes the applications and research progress of CAR‐T, chimeric autoantibody receptor T (CAAR‐T), and CAR regulatory T (CAR–Treg) therapies in AIDs, while critically discussing current challenges and future directions. By integrating the latest clinical and preclinical evidence, we aim to address the gap in systematic integration of mechanism, efficacy, and safety data—an unmet need given the fragmented nature of existing reviews that often focus on isolated disease subtypes or technical aspects.

The subsequent sections follow a logical sequence: first, we elaborate on the mechanisms underlying CAR‐T‐mediated immune reset in AIDs; second, we synthesize clinical progress across major rheumatic diseases and AIDs subtypes; third, we analyze core challenges including manufacturing complexity, tissue penetration limitations, and long‐term immunological sequelae; fourth, we highlight emerging innovative strategies such as universal CAR‐T (UCAR‐T), in vivo‐generated CAR‐T, and multitargeted designs; finally, we provide a forward‐looking perspective on precision translation guided by biomarkers and multiomics technologies. This structure aims to offer a comprehensive, mechanism‐driven framework for understanding and advancing CAR‐T therapy in AIDs.

## 2 Targeting B Cells as a Therapeutic Strategy for SRDs and AIDs

SRDs and AIDs are a group of disorders caused by the immune system mistakenly recognizing and attacking self‐tissues, with potential pathogenic factors related to AIDs not completely understood involving three main pathological factors [18]: (1) the presence of autoantibodies; (2) disease‐associated autoreactive lymphocytes; (3) reduced or dysfunctional regulatory T cells (Tregs) mediating immune tolerance. Autoantibodies can mediate tissue damage through mechanisms such as complement‐dependent cytotoxicity (CDC), antibody‐dependent cell‐mediated cytotoxicity (ADCC), and immune complex deposition [19]. Pathological cells associated with AIDs mainly include B cells producing autoantibodies, activated T cells, and antigen‐presenting cells (APCs) [20]. B cells play a critical role in the progression of various AIDs by producing autoantibodies, releasing proinflammatory cytokines, and acting as APCs to activate autoreactive T cells [21]. Tregs can directly inhibit the activation and proliferation of autoreactive cells, regulate the immune system, prevent immune abnormalities, and are crucial for maintaining peripheral tolerance [22, 23]. Tregs with high CD25 expression can inhibit the activation of effector T cells by competitively binding IL‐2, secrete anti‐inflammatory cytokines such as IL‐10, IL‐35, and TGF‐beta, and induce APC antigen‐mediated cell killing through CTLA4, thereby inhibiting APC activation and response to T cells [24]. Therefore, despite the varied clinical presentations of SRDs and AIDs, the common pathogenic mechanism involves the breakdown of immune tolerance, leading to the production of autoreactive T cells, B cells, and autoantibodies, ultimately causing inflammation and tissue damage [25]. For such diseases, the ideal treatment goal is to deeply eradicate pathogenic autoreactive cells and pathogenic antibodies while preserving normal immune function. Among these, B cells play a significant role in the development and progression of AIDs through pathways such as producing autoantibodies, antigen presentation, and cytokine release [26]. Therefore, deep B cell depletion is considered crucial for disease control and even achieving immune reconstitution. Traditional treatments like glucocorticoids and immunosuppressants (ISs) lack specificity in immune system suppression, leading to generalized immunosuppression and increased infection risks [26]. In contrast, targeted B cell therapy (e.g., rituximab [RTX]) can eliminate B cells by inducing apoptosis and ADCC [27]. Based on current retrospective studies and clinical experience, monoclonal antibodies targeting B cells have shown efficacy in AIDs [28]. However, a considerable proportion of patients do not respond to treatment [29], and some effectively treated patients often experience disease relapses upon treatment cessation, while others may not continue treatment due to the development of antibodies against the drug or severe infusion reactions. Currently, the main drugs for B cell‐targeted therapy are first‐generation anti‐CD20 monoclonal antibodies. However, CD20 is only expressed in pre‐B cells to effector and memory B cell stages, whereas plasma cells secreting autoantibodies and LLPCs associated with disease relapse do not express CD20, meaning that targeting CD20 antibodies cannot effectively clear these cells [30]. Additionally, B cells in bone marrow and lymphoid tissues cannot be completely eradicated. Gomez Mendez et al.’s posthoc analysis of the LUNAR study demonstrated that patients with complete peripheral B cell depletion had better treatment responses than those with partial clearance. Thus, how to effectively reduce the number of B cells while achieving comprehensive clearance of various B cell subgroups, including plasma cells, remains a major challenge in treating AIDs. Antiplasma cell therapy holds significant value in addressing this challenge. Clinical trials have shown that CD38 antibodies (such as daratumumab) improve clinical symptoms in refractory SLE (rSLE) patients by targeting high CD38‐expressing plasma cells and eliminating antibody‐producing plasma cells [31]. Telitacicept, as a biological agent targeting both B cell activation factor and proliferation‐inducing ligands, can comprehensively inhibit B cell maturation and plasma cell differentiation [32]. A single‐center retrospective study involving 30 lupus nephritis (LN) patients with poor response or adverse reactions to conventional steroid therapy, who were treated with telitacicept on top of standard therapy for at least 24 weeks, showed a high response rate of 86.67% in patients with a SLE Responder Index (RI) of 4 at the end of the treatment, further confirming the effectiveness of antiplasma cell therapy [33]. Therefore, targeting B cells is an important approach for treating AIDs.

Human B cells primarily develop in the bone marrow and migrate to peripheral blood upon maturation. Throughout their developmental process, distinct surface antigens and DNA rearrangements categorize them into: pro‐B cells, pre‐B cells, immature B cells, transitional B cells, naive B cells, memory cells, plasmablasts, and plasma cells. The commonly targeted antigens on B cells include CD19 (expressed throughout B cell lifespan), CD20 (not expressed in pro‐B cells and plasma cells), CD38 (expressed in plasmablasts and plasma cells), and CD138 (expressed in plasma cells) [34]. Specific surface molecules on B cells such as CD19, CD20, CD21, CD22, and CD23 have multiple monoclonal antibodies developed against them, although only a few have proven successful [35]. Indirect strategies targeting B cells primarily involve disrupting interactions between T and B cells, inhibiting B cell activation, proliferation, and differentiation, antagonizing key inflammatory immune factors, and targeting immune cells associated with B cell activation like T lymphocytes [36, 37].

**FIGURE 2:**
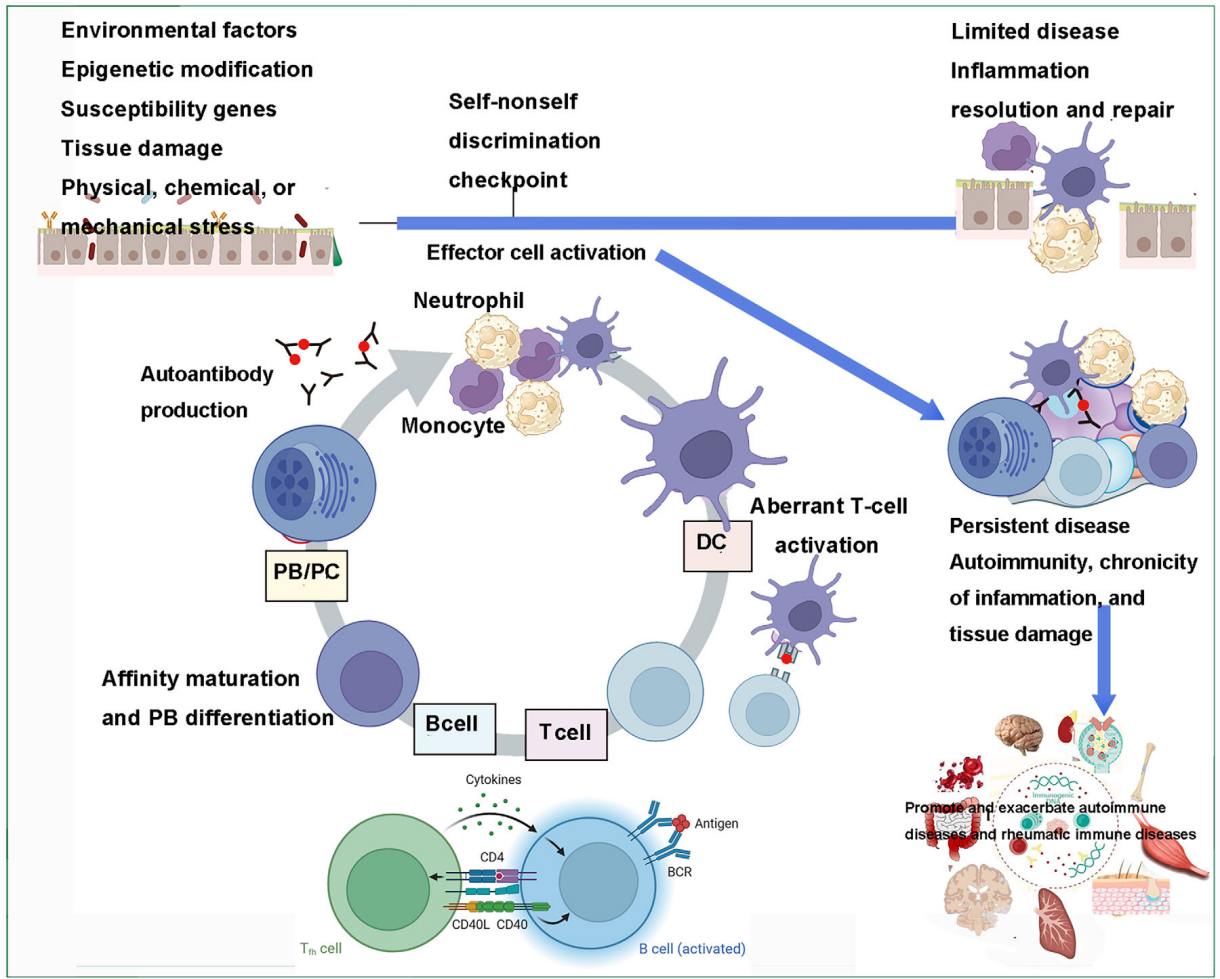
Pathogenesis of AIDs and rheumatic immune disorders: the interaction between environmental factors and genetic predispositions results in inflammatory immune‐mediated tissue damage. Subsequent to tissue injury, an acute inflammatory response is initiated, orchestrated by neutrophils and monocytes (activated effector cells), and is typically self‐limiting, giving way to tissue repair mechanisms. In autoimmune conditions, however, a self‐sustaining cycle is established, characterized by dendritic cell‐mediated presentation of self‐antigens, activation of autoreactive T lymphocytes, affinity maturation of B cells, and the production of autoantibodies by plasma cells. These autoantibodies maintain effector cell activation and perpetuate tissue damage. Once this autoimmune cycle is established, it contributes to the chronicity of the disease. (This figure was created using BioRender.com.)

In conclusion, current research indicates that the main pathogenic basis for most AIDs lies in autoreactive B cells producing autoantibodies. Using this mechanism, B cell depletion therapies (BCDTs) targeting B cell surface antigen CD20, such as the drug RTX, have shown efficacy in both cancer treatment and various AIDs. Subsequent studies have revealed that some T cell‐dependent AIDs are also responsive to BCDT strategies. The reason is that besides directly producing pathogenic autoantibodies, B cells exhibit other functions such as cytokine release and antigen presentation that activate autoreactive T cells, contributing to pathological alterations (Figure 2) and ultimately leading to disease onset.

## 3 Overview of CAR‐T Cell Therapy

This section provides a foundational overview of CAR‐T cell therapy to establish the technical framework for subsequent discussions on its application in AIDs. It is structured to first elaborate on the core biological principles and structural evolution of CAR‐T cells (Section 2.1), followed by a detailed breakdown of the clinical implementation process and emerging technical variants (Section 2.2). Specifically, Section 2.1 outlines the four key structural components of CARs and traces the developmental progression from first‐ to fifth‐generation CAR‐T cells, highlighting improvements in activation signaling, costimulatory domains, and functional modifications (e.g., cytokine integration) alongside their respective advantages and limitations in safety and efficacy. Section 2.2 systematically describes the standardized clinical workflow of CAR‐T therapy—including T cell collection, genetic transduction, ex vivo expansion, and in vivo infusion—while also introducing alternative cell sources (e.g., γδ T cells, NK cells) and novel derivative technologies (e.g., CAR–Tregs, CAAR‐T) that expand the therapeutic landscape beyond conventional autologous CAR‐T. Together, these two subsections aim to equip readers with a comprehensive understanding of CAR‐T technology's basic mechanisms, technical maturity, and translational potential, laying the groundwork for analyzing its specific applications and challenges in SRDs and AIDs.

### 3.1 Principles and Construction of CAR‐T Cells

CARs are fusion proteins obtained through genetic recombination techniques, comprising four main components: an extracellular target binding domain (ectodomain), a hinge region, a transmembrane domain anchoring the CAR to the cell membrane, and an endodomain for transducing T cell activation signals. The extracellular portion binding to tumor antigens consists of a single‐chain variable fragment (scFv) derived from the variable regions of heavy and light chains of monoclonal antibodies, enabling T cells expressing specific CARs to recognize surface antigen molecules targeted by the scFv‐derived antibodies. This structural feature expands the range of antigens recognized by CAR‐T cells to include not only protein molecules but also glycolipids. The intracellular signaling domain of CARs originates from the T‐cell receptor (TCR) complex and costimulatory molecules [38]. First‐generation CARs contained only the CD3ζ domain from the TCR complex in the intracellular region, transmitting activation signals to T cells, referred to as “signal 1”[39]. Due to the absence of costimulatory signals, first‐generation CAR‐T cells exhibited limited proliferation in vivo, short survival times, severely restricting their efficacy in tumor treatment. Building upon the TCR‐ζ intracellular domain, Finney et al. [40] included the intracellular domain of the costimulatory molecule CD28, leading to second‐generation CAR‐T cells that significantly increased the production of cytokines like IL‐2 compared with first‐generation CAR‐T cells. CARs with an additional costimulatory signal on top of the first generation are termed second‐generation CARs, where this additional signal is known as “signal 2.”

Apart from CD28, the costimulatory domain of second‐generation CARs can also come from molecules like CD27, OX40 (CD134), 4‐1BB (CD137), Lck, DNAX‐activating protein 10 (DAP10), and ICOS. CARs containing more than one costimulatory molecule are referred to as third‐generation CARs. Studies have shown that the proliferation, long‐term survival, cytokine secretion, and tumor clearance abilities of second‐generation and third‐generation CAR‐T cells significantly improve upon antigen activation [41]. Third‐generation CARs, however, possess a lower activation threshold, making them prone to off‐target effects, such as targeting normal tissues, alongside tumor cells. Thus, current clinical trials predominantly use second‐generation CARs. Further evidence is necessary to ascertain whether third‐generation CAR‐T cells outperform second‐generation CAR‐T cells in terms of functionality and safety.

**FIGURE 3:**
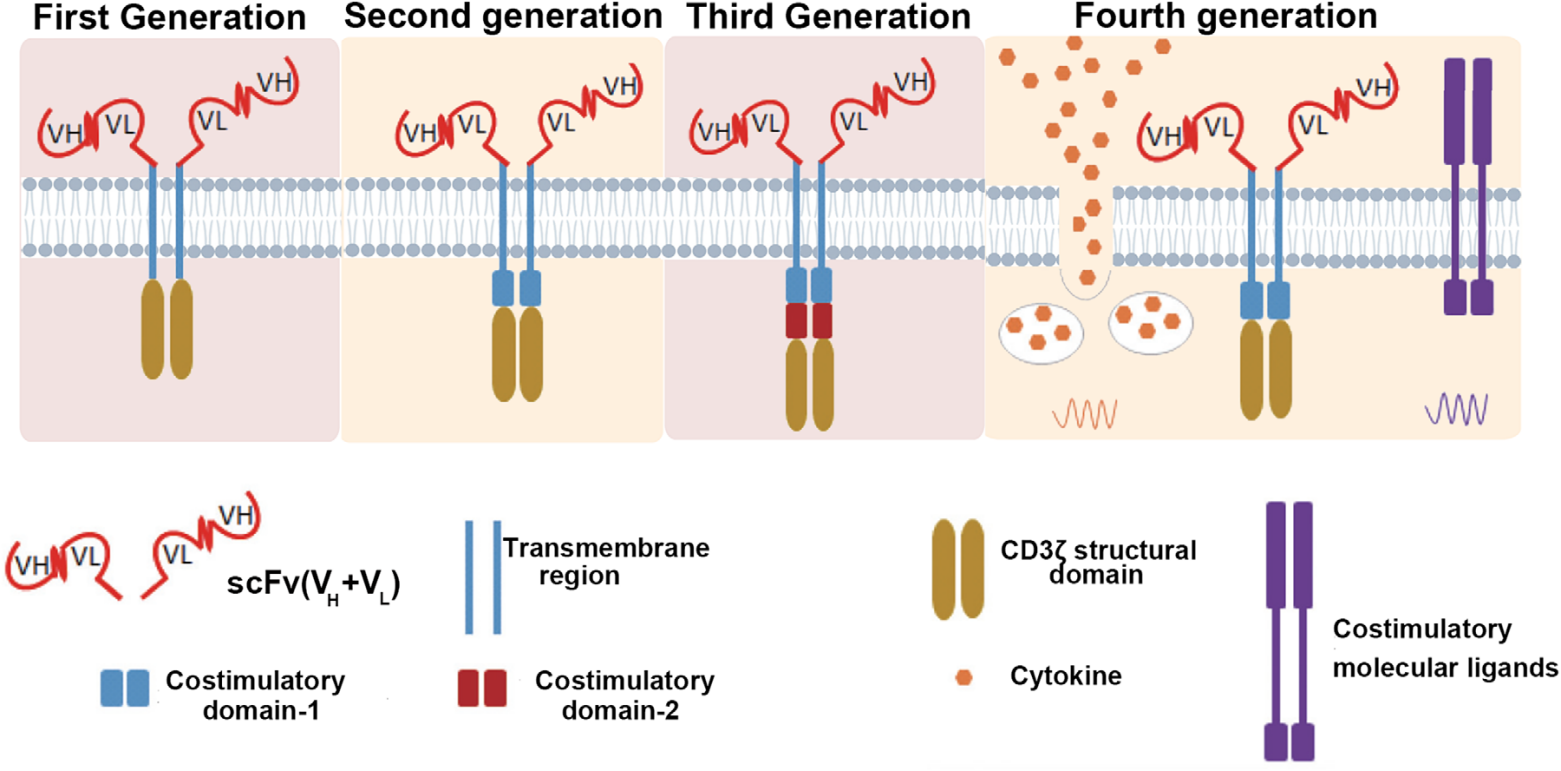
The progression of CAR‐T technology. The first generation consisted of an antigen‐binding domain directly linked to the intracellular CD3ζ chain of the TCR complex. These first‐generation CARs initiated activation cascades through the phosphorylation of immunoreceptor tyrosine‐based activation motifs (ITAMs) within CD3ζ. However, this signaling was insufficient for comprehensive T‐cell activation, necessitating the administration of exogenous cytokines. As a result, early clinical trials produced suboptimal outcomes. The second generation incorporated costimulatory domains, such as CD28 and 4‐1BB, into the CAR's intracellular segment, significantly enhancing T‐cell activation, proliferation, and survival. This advancement was clinically validated in studies showing that CD28 costimulation in CD19‐targeted CAR‐T cells markedly improved cellular expansion and persistence. The CD28‐ and 4‐1BB‐based second‐generation constructs were developed in 2003 and 2004, respectively. The third generation integrated multiple signaling domains to synergistically amplify T‐cell activation and durability. However, the clinical superiority of these tripartite signaling architectures remains a subject of debate. The fourth generation was engineered with additional modifications, although the sentence is incomplete and further details are required for a comprehensive understanding. (This figure was created using BioRender.com.)

In response to challenges encountered in CAR‐T therapy, researchers have explored modifying the CAR structure. Fourth‐generation CARs, built on the foundation of second/third‐generation CARs, incorporate gene segments encoding specific cytokines and chemokines in the intracellular domain. This modification enables stimulated CAR‐T cells to produce or induce the secretion of particular cytokines, promoting T cell proliferation, activation, enhancing cytotoxicity, inducing nonspecific antitumor immunity and increasing CAR‐T cell infiltration into tumors, thus further augmenting therapeutic efficacy. These cytokines include IL‐2, IL‐12, IL‐15, IL‐21, and CCL19 [42]. These CAR‐T cells are also known as “T‐cell redirected for universal cytokine‐mediated killing (TRUCKs)” or “armored CAR‐T cells” (Figure 3).

CAR technology has progressed to the fifth generation, primarily by enhancing downstream signaling pathways upon inducing cytokine signals. This novel CAR‐T cell, upon antigen‐specific activation, can activate the JAK kinase and STAT3, STAT5 transcription factor signaling pathways. Activation of these pathways can inhibit terminal differentiation of CAR‐T cells and enhance their proliferation. In animal models of hematologic and solid tumors, long‐term survival and tumor‐killing functions of these fifth‐generation CAR‐T cells significantly surpass traditional CAR‐T cells [43]. These structural optimizations and functional modifications continually enhance the efficacy and safety of CAR‐T cells, making the optimization of CARs for different tumors a burgeoning research area [12]. Given that third‐, fourth‐, and fifth‐generation CAR‐T cells enhance immune activation and antitumor capabilities but also increase the occurrence of adverse effects, current clinical practice still predominantly employs second‐generation CAR‐T cells.

### 3.2 Clinical Steps of CAR‐T Cell Therapy

In clinical practice, achieving effective and standardized CAR‐T cell immunotherapy requires the following steps: collecting a sufficient number of peripheral blood T cells from the patient; safely and efficiently introducing CAR‐related genetic material into T cells; amplifying genetically modified T cells ex vivo to reach the required quantity for clinical treatment; transferring therapeutic T cells into the patient's body to circulate to tumor sites, expand within the body, persist for a certain period to achieve beneficial antitumor immune responses. The gene sequence encoding CAR is typically transferred into cells in the initial stages of T cell expansion ex vivo by viral vectors or nonviral vectors (such as lipid nanoparticles [LNPs], CRISPR–Cas9 gene‐edited transduction to introduce CAR genetic information into activated cells). Commonly used viral vectors include gamma retrovirus and lentivirus, which can integrate the gene sequences into the host cell genome, ensuring permanent transgene expression. Nonviral gene transfer methods include transposon/transposase systems like sleeping beauty and RNA electroporation [44]. Compared with viral vectors, nonviral vectors offer higher safety, relatively lower costs, and can also ensure high‐level, permanent expression of the target gene. To achieve optimal expansion of CAR‐T cells ex vivo, typically, anti‐CD3 antibody alone or in combination with costimulatory antibodies such as anti‐CD28 antibodies are administered, or cell factors like IL‐2, IL‐7, IL‐12, or IL‐15 during the culture process. Alternative approaches involve using artificial APCs, such as irradiated K562 cells or EBV‐transformed cells [45]. Following intravenous infusion, CAR‐T cells redistribute rapidly in the body. CAR‐T cells can enter bone marrow, lymph nodes, and other tissues with target antigens, recruiting or proliferating in tumor tissues through circulation. Like normal T cells in the body, CAR‐T cells develop specific immunological memory upon stimulation by tumor antigens, persisting long term in the patient's circulation. The presence of anti‐CD19 CAR‐T cells in the patient's blood circulation can last up to 4 years [46].

With continuous optimization and improvement, the preparation and treatment procedures of CAR‐T cells have become more standardized. After infusion into the patient's body, CAR‐T cells circulate to tumor tissues, recognize tumor antigens, become activated, proliferate, release cell factors, ultimately lyse tumor cells, and persist in the patient's body as memory T cells, preventing tumor recurrence and enhancing treatment efficacy. Furthermore, besides autologous CAR‐T cell therapy, allogeneic CAR‐T cell therapy, and in vivo CAR‐T cell therapy are also emerging [15, 47]. T cells are a common cell source for CAR‐T cell therapy, while γδ T cells and natural killer (NK) cells are also used [48]. Various therapies continue to emerge, including CAAR cells, chimeric autoantigen TCR cells, and “super CAR.” CAAR‐T cells, similar to CAR‐T cells, express autoantigens on T cells to target B‐cell receptor (BCR) on the surface of B cells specifically to eliminate B cells causing autoimmune reactions without affecting normal B cell function [49]. CAR–Tregs, loading CAR structures onto Treg cell surfaces, target autoimmune sites, exert immune suppression by Tregs, and restore immune balance at autoimmune sites without inducing systemic immune tolerance [50, 51]. Compared with traditional immune modulation therapies, CAR–Treg therapy more accurately targets and regulates immune responses at lesion sites, promoting the reestablishment of immune tolerance in patients with AIDs and reducing interference with normal immune function. CAR‐T cells can also form memory T cells, providing long‐lasting therapeutic effects crucial for the long‐term management and control of AIDs, possibly reducing the risk of relapse [52, 53, 54].

## 4 Role and Mechanisms of CAR‐T Therapy in SRDs and AIDs

**FIGURE 4:**
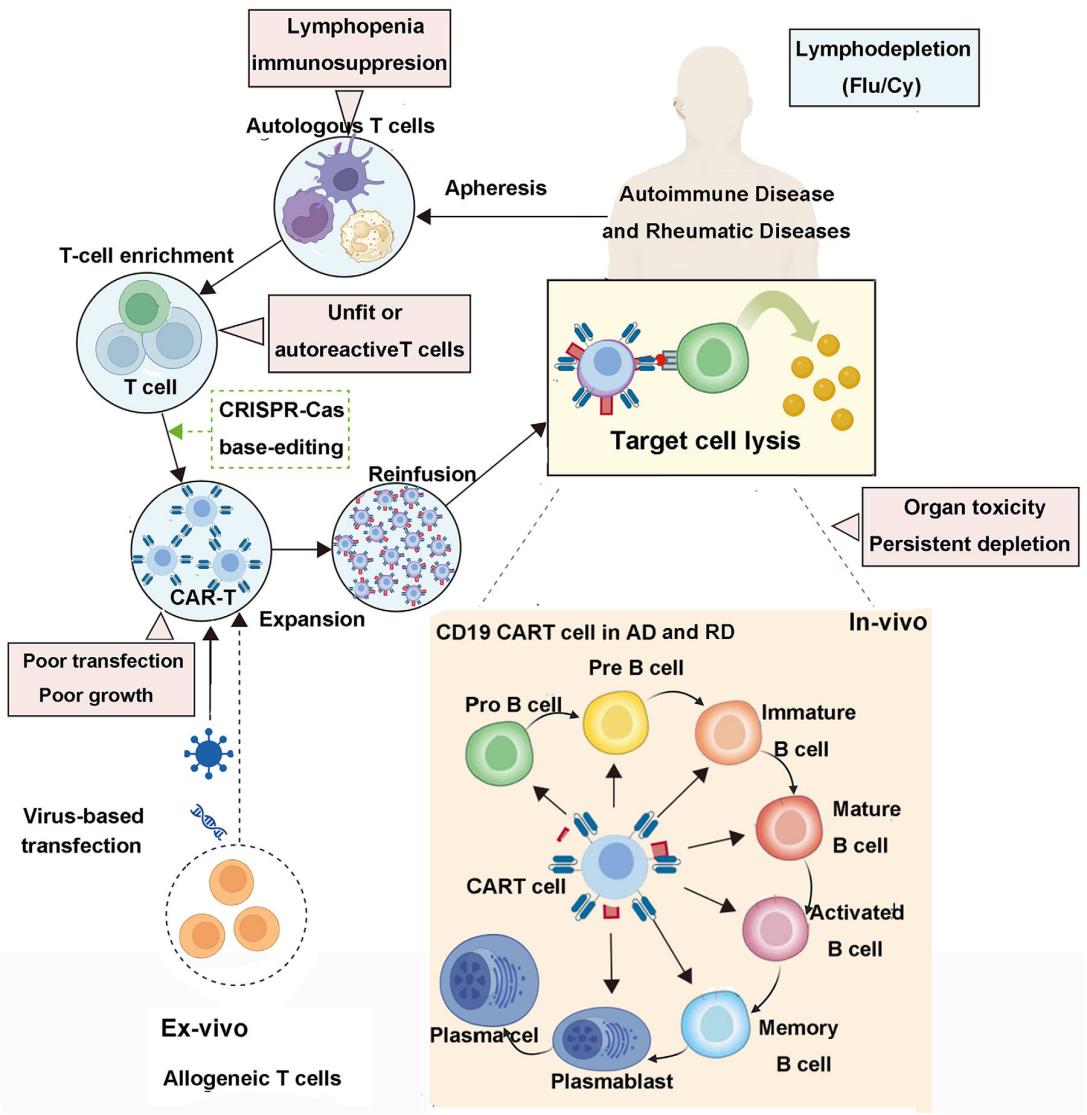
The mechanism of autologous CAR‐T cell therapy for the treatment of autoimmune and rheumatic diseases. The process comprises four key steps: Step 1 involves the collection and isolation of T cells, which are harvested from the patient, typically through peripheral blood separation. Step 2 is the genetic engineering phase, where genes encoding specific antigen recognition domains, known as CAR genes, are introduced into the T cells using viral or nonviral vectors in a laboratory setting. Step 3 encompasses ex vivo expansion and reinfusion, during which the genetically modified T cells undergo large‐scale proliferation under controlled laboratory conditions to achieve a sufficient quantity for therapeutic application. Subsequently, the expanded CAR‐T cells are reintroduced into the patient via intravenous infusion or alternative delivery methods. Step 4 entails the specific recognition and elimination of target cells. The CAR‐T cells employ their surface CAR structures to precisely identify tumor‐associated antigens on target cells. Upon recognition, the CAR‐T cells become activated, proliferate, and exert cytotoxic effects. (This figure was created using BioRender.com.)

Given the fundamental principles of CAR‐T cell therapy, it is inferred that it holds therapeutic potential in AIDs. Compared with traditional ISs, CAR‐T cell therapy may have advantages in terms of depth, efficacy, and persistence in clearing B cells [55, 56]. Targeting CD19 or BCMA with CAR‐T cell therapy is at the core of treating B cell‐driven SRDs and AIDs (e.g., SLE, autoimmune neurological disorders, Sjogren's syndrome, idiopathic inflammatory myopathies [IIMs], RA, and SSc) by achieving profound B cell depletion (elimination of self‐reactive B cell clones) [57]. Researchers have validated its therapeutic efficacy in animal models, such as observing a decrease in anti‐double‐stranded DNA (dsDNA) antibody levels, reduced urine protein levels, and decreased immune complexes in kidney tissues in SLE mouse models [58, 59]; effective disease suppression in autoimmune arthritis mouse models with CD8 CAR‐T cell therapy [60]. Additionally, considering inherent T cell abnormalities and the impact of ISs on T cells in immune disease patients, Kretschmann et al. [61] conducted exploratory CAR‐T cell therapy studies in SLE patients, demonstrating successful isolation and strong expansion capabilities of T cells in six SLE patients. Based on these findings, CAR‐T cell therapy has commenced its preliminary clinical application in AIDs (Figure 4).

### 4.1 Mechanisms of CAR‐T Therapy in SLE/LN

SLE is a systemic AID characterized by autoimmune inflammation, impacting over 3.4 million individuals worldwide. The disease primarily affects women, with a ratio of approximately 9:1 compared with men. Although SLE can occur at any age, it is most common in reproductive‐age women, typically between 15 and 44 years old; childhood‐onset SLE (defined as onset before 18 years old) often presents more severe clinical courses [62]. Traditional therapies for SLE include corticosteroids, antimalarials, nonsteroidal anti‐inflammatory drugs, cytotoxic drugs, and immunosuppressive agents, but long‐term tolerability issues with these treatments affect their efficacy, leading to poor disease control, organ damage, and impacting prognosis and long‐term survival, notably in moderate to severe cases requiring high‐dose steroids and ISs [63]. Consequently, challenges persist in clinical efficacy and long‐term disease control with BCDT, paving the way for targeted and durable new therapies. A milestone event in 2021 was the successful treatment of a 20‐year‐old rSLE patient using CD19 CAR‐T cell therapy, showing complete clinical and serological remission 44 days posttreatment, with a reduction in SLE disease activity index (SLEDAI) from 16 to 0, enabling steroid discontinuation over a 6‐month follow‐up period without disease relapse [64]. Subsequently, in 2022, this study group included five rSLE patients aged medianly 22 years, with all patients recovering normal complement levels and anti‐dsDNA antibody titers by the 3‐month follow‐up after CAR‐T cell reinfusion; four patients achieved an SLEDAI of 0, and one patient achieved a score of 2, without relapse during a 17‐month follow‐up period, achieving drug‐free remission with only one case of Grade 1 cytokine release syndrome (CRS) event observed during treatment [65]. To further assess its long‐term efficacy and safety, results from a study involving 15 severe AID patients receiving CD19 CAR‐T cell therapy, including eight with SLE, were reported in 2024 [66]. This research showed that all patients achieved a low disease activity status in SLE at 6 months post‐CAR‐T cell therapy, with SLEDAI scores of 0 persisting up to 29 months follow‐up while experiencing only mild upper respiratory tract infection symptoms in the safety evaluation. These clinical studies confirm the effectiveness, durability, and good tolerability of CAR‐T cell therapy in the treatment of adult SLE.

Adolescent‐onset SLE often presents with higher disease activity and medication burden, more susceptible to renal, hematologic, and neurological involvement, with a higher mortality rate compared with adult‐onset [67]. Therefore, early intervention and exploration of new treatment strategies are crucial within the pediatric and adolescent population. Krickau et al. [64] applied CAR‐T cell therapy to a 15‐year‐old rSLE female patient who had received various treatments including steroids, ISs, belimumab, and plasma exchange, as her renal function deteriorated progressively (severe proteinuria, creatinine up to 150 µmol/L, hyperphosphatemia, renal tubular acidosis), requiring hemodialysis maintenance, with a SLEDAI score of 23. Following treatment, the patient's renal function improved rapidly, with discontinuation of hemodialysis by the 17th day, complement C3 and C4 levels normalized within 6 weeks, and relevant autoantibodies disappeared, experiencing only transient Grade 4 neutropenia and Grade 1 CRS. By the fourth month of CAR‐T cell therapy, the patient had returned to normal life, achieving drug‐free remission. It is noteworthy that for LN patients, creatinine and urine routine tests may not accurately reflect the actual severity of renal damage, making posttreatment renal biopsy more compelling. Recent efforts by de Benedetti et al. [68] in this regard have been promising. In their study, successful application of CD19 CAR‐T cell therapy in a 16‐year‐old rSLE patient exhibited significant clinical efficacy, with follow‐up kidney biopsies after 6 months showing clearance of C3, C1q, and IgG deposits in the glomeruli.

Shu et al. [69] reported a Phase I clinical trial evaluating relmacabtagene autoleucel, a commercially available CD19‐directed CAR‐T cell product, in patients with moderate‐to‐severe active SLE. The trial (NCT05765006) enrolled eight female patients whose disease remained active despite standard‐of‐care therapies; all exhibited multiorgan involvement at baseline, with some presenting concomitant LN. Following lymphodepletion with fludarabine and cyclophosphamide (CTX), patients received a single infusion of CAR‐T cells at escalating doses (25, 50, 75, or 100 × 10^6^ cells). No dose‐limiting toxicities (DLTs) were observed. The majority of adverse events (AEs) were Grade 1–2 (mild to moderate); the most common included cytopenias ≤Grade 3 (incidence: 100%) and CRS (CRS; incidence: 88%, predominantly Grade 1). One patient developed immune effector cell‐associated hemophagocytic lymphohistiocytosis‐like syndrome (IEC‐HS), which resolved promptly with appropriate intervention. All patients demonstrated profound B‐cell depletion and rapid clinical improvement. Notably, 100% of patients achieved an SRI‐4 response within 1–4 months postinfusion; seven met criteria for Lupus Low Disease Activity State (LLDAS), and four fulfilled the DORIS definition of clinical remission. Importantly, all patients remained free of glucocorticoids and ISs throughout follow‐up. Collectively, these findings provide preliminary evidence of the safety, tolerability, and clinical efficacy of relmacabtagene autoleucel in patients with moderate‐to‐severe active SLE.

*CD19/BCMA Dual‐Targeted FAST CAR‐T (GC012F/AZD0120): Preliminary Phase I Data in Refractory SLE*. At the 2025 European Hematology Association Congress, AstraZeneca/Gracell presented early results from a first‐in‐human Phase I trial (NCT05846347) evaluating GC012F/AZD0120—a CD19/BCMA dual‐targeted, FasTCAR‐T platform‐derived CAR‐T therapy—in patients with rSLE. Eligible patients had SLEDAI‐2K scores ≥8 and had failed at least two ISs and one biologic agent. As of November 12, 2024, 15 rSLE patients had been enrolled and treated across escalating dose levels (DL1: *n* = 3; DL2: *n* = 3; DL3: *n* = 9). Median follow‐up was 384 days (range: 172–526 days). Median age was 28 years (range: 22–55); 93% (14 out of 15) were female; median disease duration was 7.3 years (range: 1.3–23.7). Eleven patients had biopsy‐proven LN. Baseline median SLEDAI score was 12 (range: 6–26). No DLTs were observed. CRS occurred in 14 patients (median onset: Day 5.5, range 3–10; median duration: 4.5 days, range 2–16), with 12 cases being Grade 1. Two patients in the 3 × 10^6^ CAR^+^ T cells/kg cohort developed Grade 3 CRS, both successfully managed with methylprednisolone. One patient experienced Grade 2 immune effector cell‐associated neurotoxicity (NT) syndrome (ICANS) on Day 7, manifesting as ataxia (ICE score: 10/10), which resolved with dexamethasone and levetiracetam. Seven patients developed infections (all Grade 1–2), none life‐threatening. All patients discontinued glucocorticoids, ISs, and biologics prior to infusion. Improvements were observed in autoantibody profiles (including anti‐dsDNA) and complement levels, accompanied by sustained reductions in SLEDAI‐2K scores. At Month 6, 33% (five out of 15) of evaluable patients achieved DORIS remission; this increased to 45% (five out of 11) at Month 9 and 56% (five out of nine) at Month 12. Nonresponders included patients with residual proteinuria or one nonrenal patient who relapsed at Month 6. These preliminary data suggest favorable early safety and promising efficacy of dual‐targeted CAR‐T therapy in rSLE. An ongoing Phase I/II trial (NCT06530849) is further evaluating its safety and efficacy [70].

*Rapcabtagene Autoleucel (YTB323) in Severe Refractory SLE: 12‐Month Clinical and Biomarker Outcomes*. Novartis is developing YTB323 (rapcabtagene autoleucel), a rapidly manufactured autologous CD19 CAR‐T therapy previously shown to have a favorable risk–benefit profile in hematologic malignancies. At the 2025 EULAR Annual Meeting, interim results from an ongoing open‐label Phase 1/2 study [71] were presented, detailing clinical, cellular kinetics, pharmacodynamic, and biomarker outcomes in patients with severe rSLE (srSLE) over 12 months posttreatment. As of data cutoff, 21 patients had been enrolled; updated results including extended follow‐up will be presented at the conference. Baseline median age was 36 years (range: 24–54); 12 out of 13 reported patients were female. Median follow‐up was <4 months, with one patient followed to Month 12 and three to Month 9. Among the three patients with ≥9 months of follow‐up, marked clinical improvement was observed: mean SLEDAI‐2K reduction of 14.7 points, decreased anti‐dsDNA titers, and increased C3 levels. Cellular kinetics and pharmacodynamic data from 13 patients revealed peak CAR‐T expansion at 2–3 weeks postinfusion, followed by profound B‐cell depletion. B‐cell reconstitution occurred in most patients between Days 60 and 90. Early‐repopulating B cells were predominantly naïve in phenotype, with marked reductions in memory B‐cell subsets and plasmablasts. Safety data indicated YTB323 was generally well tolerated. All patients experienced transient, lymphodepletion‐associated cytopenias (Grade 3/4); no Grade 4 neutropenia or lymphopenia persisted beyond 28 days. Eight out of 13 patients developed Grade 1/2 CRS, all resolved with tocilizumab (*n* = 7) or supportive care (*n* = 1). One patient developed Grade 2 ICANS (ataxia; ICE score 10/10), which resolved within 4 days of corticosteroid initiation. Three serious AEs (SAEs) were reported: two treatment related (CRS, pneumonia) and one unrelated (urinary tract infection); all resolved completely. In summary, YTB323 induced significant reductions in disease activity, achieved durable B‐cell depletion, and promoted immune reconstitution dominated by naïve B cells. Early clinical data support its robust efficacy in srSLE, with a safety profile consistent with prior reports of CD19 CAR‐T therapies in AIDs.

*GC012F (AZD0120) in Refractory SLE: Dose‐Escalation IIT with Extended Follow‐Up*. At the 2025 EULAR Annual Meeting, Gracell Biotechnologies reported interim results from an investigator‐initiated, dose‐escalation trial (NCT05858684) evaluating GC012F—a next‐generation, dual‐targeted CD19/BCMA CAR‐T therapy manufactured within 24 h via the FasTCAR‐T platform—in patients with refractory active SLE [72]. As of November 12, 2024, 10 patients (SLEDAI‐2K: 8–20) had received GC012F between June 2023 and April 2024 at doses of 1.0 × 10^5^ (*n* = 4), 2.0 × 10^5^ (*n* = 3), or 3.0 × 10^5^ CAR‐T cells/kg (*n* = 3). Patients were predominantly young (median age: 26.5 years, range: 19–42) and all had biopsy‐confirmed LN (Class III: 1; IV: 5; V: 1; III + V: 1; IV + V: 2); median disease duration was 6 years (range: 2–19). Median follow‐up postinfusion was 313.5 days (range: 211–487). No DLTs occurred. Seven patients experienced CRS (Grade 1: *n* = 6; Grade 2: *n* = 1), with median onset at Day 7 (range: 6–14) and median duration of 1 day (range: 1–4). The single Grade 2 CRS event (in the 3.0 × 10^5^/kg cohort) occurred on Day 7 and resolved by Day 8 with dexamethasone and tocilizumab. No ICANS or ≥Grade 3 CRS was observed. Eight patients developed infections (mostly Grade 1–2); one patient (1.0 × 10^5^/kg) experienced Grade 3 sinusitis and pneumonia, resolved with antibiotics. Robust CAR‐T expansion was observed, with median *C*_max_ of 20,180 copies/µg DNA (range: 11,482–50,316) at median *T*_max_ of 10 days (range: 7–11). Complete B‐cell depletion (0 cells/µL) was achieved in all patients, with nadir at Day 10 (range: 7–10) and reconstitution beginning at Day 84 (range: 84–168); repopulating B cells were predominantly naïve. All patients discontinued ISs and biologics preinfusion. Postinfusion, glucocorticoids (prednisone 5–20 mg/day) and/or stable‐dose hydroxychloroquine were used per disease activity. By Month 9, seven out of 10 patients had discontinued glucocorticoids; four also discontinued hydroxychloroquine; three remained on prednisone 5 mg/day. Complement levels normalized in all patients. Seven out of 10 achieved sustained seronegativity for ANA, ENA panel, and anti‐dsDNA by Month 9. As of January 2025, nine out of 10 patients met DORIS remission criteria at Month 9. Proteinuria markedly decreased in all; six achieved complete renal response (CR), and four achieved partial response (PR) per KDIGO 2024 criteria. Three of four patients with residual proteinuria underwent repeat renal biopsy at Months 6–9, revealing near‐absent active inflammation, disappearance of immune complex deposits, and restoration of podocyte foot processes—suggesting residual proteinuria reflected prior structural damage rather than active lupus, and thus was excluded from SLEDAI‐2K scoring. The remaining nonbiopsied patient had SLEDAI‐2K = 4.

In conclusion, GC012F demonstrates potent efficacy and favorable early safety in rSLE. A multicenter Phase 1/2 trial (NCT06530849) is ongoing to further evaluate its safety and efficacy in a broader SLE population.

*Rese‐cel (CABA‐201) in SLE: Immune Reset via Transient, Deep B‐Cell Depletion*. Cabaletta Bio presented at EULAR 2025 interim results from the ongoing RESET‐SLE trial (NCT06121297), a Phase 1/2 study evaluating rese‐cel—a fully human, autologous, anti‐CD19 CAR‐T therapy designed to induce transient yet profound CD19^+^ cell depletion—in patients with nonrenal SLE and LN [73]. Six patients (four nonrenal SLE: SLE‐1 to SLE‐4; 2 LN: LN‐1, LN‐2) received rese‐cel and completed ≥1 month of follow‐up. The therapy was well tolerated: two patients (SLE‐2, LN‐1) experienced Grade 1 CRS (fever), neither requiring tocilizumab. As previously reported, patient LN‐1 developed Grade 4 ICANS related to an occult infection, which resolved rapidly with standard management. No other ICANS events occurred. All six patients showed clinical improvement (follow‐up: 1–9 months). Among nonrenal SLE patients, three achieved LLDAS by Week 4 and DORIS remission by Week 8 (SLE‐2, SLE‐4) or Week 4 (SLE‐3), maintaining both states through last follow‐up. The remaining patient (SLE‐1, with Class V LN) showed SLEDAI‐2K reduction from 26 to 8 by Week 28, with >50% reduction in UPCR (1.08 to 0.52 mg/mg). In the LN cohort, LN‐1 achieved LLDAS by Week 24; LN‐2 showed SLEDAI reduction from 14 to 11 by Week 4 (latest follow‐up). Both LN patients exhibited reduced proteinuria: LN‐1 achieved CR by Week 24 (UPCR: 7.22 → 0.45 mg/mg; prednisone <10 mg/day); LN‐2 showed 47% UPCR reduction by Week 4 (4.85 → 2.55 mg/mg). All patients discontinued SLE‐related ISs; SLE‐1 and SLE‐2 completed glucocorticoid taper; LN‐1 was on prednisone 6 mg/day at last visit. Pharmacodynamic analyses revealed peak CAR‐T expansion (*C*_max_) between Days 8 and 15 postinfusion in five out of six patients; LN‐1 exhibited a secondary peak at Day 29. Serum IFN‐γ peaked concurrently with or prior to CAR‐T *C*_max_. CAR‐T products were predominantly CD4^+^ at infusion; three patients shifted to CD8^+^ dominance at *C*_max_. Peripheral B cells were rapidly depleted within 1 month in four out of five evaluable patients. Transitional naïve B‐cell repopulation was observed in SLE‐1 by Week 8; insufficient follow‐up precluded assessment in others. Serum BAFF levels increased postinfusion in most patients. In summary, rese‐cel induced early, immunosuppression‐free clinical remission or response in SLE patients, accompanied by favorable safety (including CRS), robust CAR‐T expansion, and profound peripheral B‐cell depletion. These preliminary data support its potential to “reset” the immune system in SLE, enabling sustained clinical responses despite discontinuation of all immunosuppressive therapies and glucocorticoid tapering.

Concerning SLE‐related complications, approximately 16% of Chinese SLE patients suffer from immune thrombocytopenia (ITP), with these patients showing significantly lower long‐term survival rates compared with those without thrombocytopenia [74]. A research team from China pioneered the application of CD19 CAR‐T cell therapy for treating SLE‐related ITP [75]. In one case involving a 38‐year‐old female patient with a 10‐year history, refractory severe thrombocytopenia persisted despite multiple steroid pulses and various immunosuppressive treatments, with platelet counts consistently below 20 × 10^9^/L, accompanied by purpura, skin bruising, and gum bleeding. After CAR‐T cell infusion, the patient's platelet count rose from 4 × 10^9^/L before treatment to 29 × 10^9^/L after 1 month of treatment, reaching 109 × 10^9^/L by the sixth month, along with a rapid decline in related antibodies, allowing the gradual discontinuation of steroids and ISs. The patient experienced only transient Grade 1 fever on the ninth day postinfusion, which resolved with physical cooling.

In terms of structural optimization, a clinical study employing a complex CAR‐T cell therapy targeting both CD19 and BCMA [76] showed promising results. The team recognized the association between the severity of SLE and increased expression of the BCMA surface antigen on LLPCs, as well as the presence of CD19‐negative LLPCs in the bone marrow, hence constructing a dual‐target structure to enhance efficacy. The study revealed that in 13 SLE patients after 3 months of treatment, disease activity scores (DASs) decreased from a baseline of 10.6–2.7, significant improvement in renal function was observed, and by the 4–6 month follow‐up, all patients achieved drug‐free sustained remission, exhibiting good tolerability with only Grade 1 CRS and no observed ICANS.

On May 30, 2024, during the European Congress of Rheumatology 2024 (EULAR 2024), the latest clinical data of rujitolincept injection (a CD19‐targeted CAR‐T cell therapy) in active SLE adult patients in China were disclosed. As of April 8, 2024, the study had enrolled a total of 12 subjects who completed rujitolincept infusion, investigating safety in low, medium, and high dose groups, pharmacokinetics (PK), pharmacodynamics (PD), and efficacy, with the longest follow‐up exceeding 9 months. In the 25 × 10^6^ (25 M) dose group, three active SLE adult subjects received a single CAR‐T cell intravenous infusion and completed at least 4 months of follow‐up. Postrujitolincept infusion, these three subjects showed continual improvement in signs and symptoms, with SELENA‐SLEDAI scores decreasing from 8 to 14 at baseline to 0 or 1, all achieving an SLE RI 4 (SRI‐4), including two subjects meeting the more stringent LLDAS criteria. At the data cutoff, all three subjects had ceased using steroids and other SLE treatment drugs. PK/PD data reaffirmed the expansion and depletion of peripheral blood B cells by rujitolincept. These three patients are currently under study follow‐up, with all follow‐up periods exceeding 6 months, showing continued improvement in disease activity and clinical symptoms. The data suggest that even at significantly low doses compared with hematologic indications, a single infusion of rujitolincept injection can provide profound and sustained disease remission for moderate to severe lupus erythematosus patients, demonstrating good safety.

A landmark clinical study targeting BCMA [77] reported the first‐in‐human application of BCMA‐directed CAR‐T cells in patients with LN. Among seven enrolled SLE patients (five female, two male; median age 35 years, range 27–61), all had biopsy‐confirmed LN (median interval from biopsy to infusion: 1.5 months, range 1–5 months). Histopathological classification revealed four cases of Class IV LN, and one each of III + V, III, and IV + V. All patients had received multiple prior immunosuppressive regimens, including glucocorticoids (seven out of seven), mycophenolate mofetil (MMF; six out of seven), CTX (four out of seven), cyclosporine A (four out of seven), tacrolimus (four out of seven), belimumab (two out of seven), telitacicept (three out of seven), and three had undergone plasma exchange. Despite intensive prior therapy, disease remained highly active at enrollment, with a median SLEDAI‐2K score of 18 (range 10–22). Following BCMA CAR‐T infusion, all seven LN patients exhibited marked clinical and laboratory improvements. Five achieved drug‐free complete remission, three of whom maintained remission beyond 6 months. Notably, renal biopsy from Patient 2 at 6 months posttreatment showed substantial reduction in immune complex deposition, supporting preliminary therapeutic efficacy. Incomplete normalization of proteinuria in some patients likely reflects pre‐existing irreversible renal damage, underscoring the potential benefit of earlier CAR‐T intervention. Safety evaluation revealed no SAEs; only one case of Grade 1 CRS occurred. Transient cytopenias, attributable to lymphodepleting chemotherapy, were common but fully reversible. Hypogammaglobulinemia was universal and managed prophylactically with intravenous immunoglobulin (IVIG). No cases of ICANS were observed, affirming the favorable safety profile of BCMA CAR‐T therapy.

*Dual‐Targeted CD20/BCMA CAR‐T (C‐CAR168) in Refractory AIDs: Preliminary Phase I Data*. At the 16th International Congress on Systemic Lupus Erythematosus (LUPUS 2025), AbelZeta Pharma [78] presented preliminary results from an investigator‐initiated Phase I trial (NCT06249438) evaluating C‐CAR168, a dual‐targeted CD20/BCMA CAR‐T therapy, in patients with refractory AIDs. Ten patients received C‐CAR168: three LN patients at 1.5 × 10^6^ cells/kg, and seven others—including one with ITP, one with multiple sclerosis (MS), one with neuromyelitis optica (NMO), and four with LN—at 0.75 × 10^6^ cells/kg. C‐CAR168 demonstrated favorable tolerability across all LN patients, with only low‐grade CRS and no ICANS or severe infections. Clinically, it induced robust efficacy in highly refractory LN, including reduction in proteinuria, preservation of renal function, and improvement in both renal and extrarenal disease manifestations—including successful withdrawal of IS. Four patients completed Month 6 (M6) assessments and all met SRI‐4 response criteria. Patients C004 and C007 had not yet reached M6. Patient C009 experienced disease flare at Month 3 (M3) and withdrew. All patients discontinued ISs and biologics following lymphodepletion. Most achieved steroid‐free status postinfusion. Among six patients under active follow‐up, sustained reductions in SLEDAI, Physician's Global Assessment (PGA), and proteinuria were observed: three remained on low‐dose steroids, three were steroid‐free. Patients C002 and C003 achieved complete renal remission (LN‐CR); patient C009 relapsed at M3. Early normalization of complement levels was observed in all LN patients, with six out of seven achieving normal C3/C4 during follow‐up. Renal function remained stable, with no decline in estimated glomerular filtration rate (eGFR). Anti‐dsDNA titers declined in most patients. Robust C‐CAR168 expansion was detected in peripheral blood (median T_max: 11 days, range 7–21 days), persisting for 1–3 months in five patients (shorter duration in DL1 cohort: Patients 02 and 03). Profound and rapid depletion of circulating B cells and plasma cells was observed. Transcriptomic profiling further confirmed deep plasma cell depletion and attenuation of type I interferon (IFN) pathway activity. Bone marrow biopsy from Patient C009 revealed elimination of CD19^+^ plasma cells and long‐lived CD19^+^ plasma cells. Peripheral B‐cell reconstitution was dominated by naïve B cells, suggesting immune reset. In the single SPMS (secondary progressive MS) patient treated, no SAEs occurred. C‐CAR168 exhibited robust PK/PD profiles, with potent expansion and complete depletion of blood B cells, plasma cells, and CD20^+^ T cells. Functional improvements were noted in 9‐Hole Peg Test (9‐HPT), timed 25‐foot walk (T25‐FW), and mini‐mental state examination scores. Expanded Disability Status Scale (EDSS) scores improved, with reductions in antinuclear antibodies (ANA) and neurofilament light chain (NFL). T1‐weighted MRI showed reduction in periventricular/enhancing lesions; no new T1 or T2 lesions emerged by M3.

*Autologous CD19‐Directed CAR‐T Therapy in Pediatric Refractory SLE: A Chinese Single‐Center Experience*. Since March 2024, the Children's Hospital of Zhejiang University School of Medicine has pioneered the first domestic clinical trial of autologous CD19‐targeted CAR‐T therapy in pediatric rSLE. To date, 20 patients (16 girls, four boys; age range 6 years 8 months to 19 years; disease duration 4 months to 11 years) have been treated. The first infusion occurred on March 12, 2024; the 20th was completed by October 4, 2024 — establishing the center as China's leader in pediatric CAR‐T therapy for rSLE. Posttreatment, all patients successfully discontinued glucocorticoids and all ISs. SLEDAI‐2K scores dropped to 0 in some; all others showed marked clinical improvement, enabling return to normal daily life. CAR‐T cells expanded robustly in vivo, peaking at Days 7–10, accompanied by undetectable CD19^+^ B cells. Although B‐cell reconstitution began at 2–3 months postinfusion, immunomodulatory effects persisted. By Month 3, all patients exhibited clinical improvement; 15/20 achieved SLEDAI‐2K ≤4. All normalized complement C3 levels. Notably, all patients remained off glucocorticoids and ISs with stable disease control at last follow‐up. AEs were mild, primarily Grade 1 CRS [79].

*Allogeneic CAR‐T Therapies in SLE: Emerging Safety and Efficacy Signals*. Three pivotal studies collectively demonstrate the potential of allogeneic CAR‐T therapy in SLE. Yang et al. [80] conducted a single‐center pilot trial (NCT05988216) evaluating allogeneic CD19‐targeted CAR‐T cells (TyU19) in four young women (aged 22–24 years) with rSLE (baseline SELENA‐SLEDAI 14–26). All had multiorgan involvement; three had prior lupus cerebritis (inactive at enrollment). All had failed multiple ISs and biologics. Posttreatment, all four showed sustained clinical improvement. At 3 months, all achieved SELENA‐SLEDAI = 0 and PGA <1. Symptoms including arthritis (S02, S03, S04), alopecia (all), and digital vasculitis/ulcers (S01, S04) resolved. C3/C4 normalized within 1 month. Anti‐dsDNA titers declined; proteinuria resolved. Anti‐Sm, U1‐RNP, and Ro52 antibodies markedly decreased. In a parallel study using the same TyU19 product in three severe SLE patients, therapy was well tolerated, with no graft‐versus‐host disease (GvHD), CRS, ICANS, or macrophage activation syndrome (MAS). CAR‐T cells expanded robustly, inducing profound B‐cell depletion and significant reduction in serum autoantibodies. All three achieved SRI‐4‐defined clinical remission—highlighting the safety and efficacy of allogeneic CAR‐T in srSLE.

Wang et al. [81] reported the first clinical use of CRISPR–Cas9‐engineered “off‐the‐shelf” allogeneic CD19 STAR‐T cells (YTS109) in five patients with srSLE and LN (diagnosed per 2019 EULAR/ACR criteria). All met primary endpoint (SRI‐4 response) by Month 3 (M3), sustained through Month 6 (M6). SELENA‐SLEDAI and PGA scores declined significantly postinfusion. Four patients achieved SELENA‐SLEDAI = 0 between Months 2 and 4, maintained through M6. Mean SLE‐DAS scores plummeted from baseline 31.30 to 7.11 at M3 and 5.35 at M6; Patients 1 and 5 achieved complete remission. Patient 4 showed PR (SLE‐DAS: 15.06 → 8.33 at M3 → 16.43 at M6). Renal BILAG improved from Grade A (severe) to B (moderate) in all; one improved to Grade C (mild). Proteinuria markedly declined; Patients 1 and 5 achieved complete renal remission (24‐h proteinuria <500 mg) within 1–2 months. Histopathology (PAS staining, immunofluorescence) revealed reduced immune complex deposition, diminished inflammatory infiltrates, and restored glomerular/tubular architecture. Transcriptomic analysis of renal tissue demonstrated downregulation of inflammatory pathways and expansion of structural cell populations—indicating a shift toward tissue repair and immune quiescence. A recent study investigated allogeneic CD19‐targeted CAR‐NK cells and reported notable efficacy in 18 patients with srSLE [82]. Of 26 enrolled (median age 38 years, median disease duration 8.5 years; 82% female), 75% had failed biologics (belimumab, telitacicept); one had undergone plasma exchange. Maximum tolerated dose was not reached. Among 18 treated, only two (8%) experienced Grade 1 CRS; no NT or CAR‐NK‐related SAEs occurred. Of 12 patients followed >12 months, 66.7% (eight out of 12) achieved DORIS remission; 75% (nine out of 12) achieved LLDAS. These data support allogeneic CAR‐NK as a viable, scalable alternative to autologous CAR‐T, potentially overcoming limitations in manufacturing, accessibility, safety, and cost.

*iPSC‐Derived Dual‐Targeted CAR‐NK (QN‐139b) in Systemic Sclerosis*. A groundbreaking study [83] reported the first clinical application of QN‐139b—an iPSC‐derived, CD19/BCMA dual‐targeted CAR‐NK cell therapy—in a patient with severe, refractory diffuse cutaneous SSc (dcSSc). The product was engineered via noncutting cytosine base editors (to avoid genomic rearrangements), with six transgenes knocked‐in (human leukocyte antigen [HLA]‐E, tEGFR, HLA‐G, IL‐2RF, aCD19 CAR, aBCMA‐CAR) and three genes knocked‐out (B2M, CIITA, CD16). Transgenes were inserted into genomic “safe harbors”; cells were derived from clonally expanded, sequenced iPSCs to minimize genotoxicity. The inclusion of tEGFR as a safety switch and deletion of CD16 (to mitigate NK‐mediated disease exacerbation) exemplify innovative safety engineering. Proprietary NK persistence and low‐immunogenicity modules enhanced in vivo expansion and potency. The patient received four infusions (6 × 10^8^ cells each) on Days 0, 3, 7, and 10 (August 4, 2024). At 6‐month follow‐up, QN‐139b demonstrated excellent safety and profound clinical efficacy: patient‐reported global improvement, significant autoantibody reduction, complement normalization, marked decline in modified Rodnan Skin Score (mRSS), and improved ACR‐CRISS scores. Single‐cell and proteomic analyses confirmed pathologic B‐cell clearance, suppression of inflammation/fibrosis, elimination of skin‐infiltrating lymphocytes, and microvascular remodeling—collectively indicating successful immune reset. This represents the world's first clinical success of iPSC‐derived CAR‐NK therapy in AID, establishing a new paradigm for “off‐the‐shelf, low‐toxicity, multitargeted” precision immunotherapy.

*In Vivo CAR‐T via Targeted Lipid Nanoparticles: A Paradigm Shift*. To overcome limitations of ex vivo CAR‐T manufacturing, novel approaches using LNPs or lentiviral vectors to deliver CAR‐encoding mRNA directly to T cells in vivo are emerging. A recent study [84] evaluated HN2301—a CD8‐targeted LNP delivering CD19 CAR mRNA. Preclinically, HN2301 successfully reprogrammed T cells in cynomolgus monkeys, achieving potent B‐cell depletion in blood and tissues with favorable safety. In a first‐in‐human trial, five female patients with rSLE (four with LN; age 31–46 years; SLEDAI‐2K 8–22; disease duration 7–18 years)—all previously treated with CTX, tacrolimus, cyclosporine, MMF, belimumab, and/or telitacicept—received escalating doses: two received 2 mg IV, followed by 4 mg single infusions repeated every 48 h for 2–3 cycles. CD8^+^CD19–CAR‐T cells were detectable in peripheral blood within 6 h; B cells dropped to <10% of baseline. With repeated dosing, B cells were fully depleted (<1 cell/µL), persisting 7–10 days before recovery. CAR‐T frequency peaked at >60% of CD8^+^ T cells 6 h postinfusion, returning to baseline within 2–3 days. Clinically, all patients exhibited sustained SLEDAI‐2K reduction, rapid decline in antinucleosome and anti‐dsDNA antibodies, and partial complement normalization. Only low‐grade CRS was observed; no NT, hepatic/renal injury, or hematologic toxicity occurred. This study provides the first proof‐of‐concept for in vivo CAR‐T generation via targeted LNP delivery in SLE. As this technology matures, it promises to revolutionize AID therapy—enabling scalable, cost‐effective, and accessible cell immunotherapy without lymphodepletion or complex manufacturing. Collectively, these studies demonstrate that allogeneic CD19‐targeted CAR‐T therapies are both safe and efficacious in rSLE, highlighting their potential as transformative therapeutics. Further investigation is warranted to evaluate long‐term durability, optimize dosing regimens, and expand applications across the spectrum of AIDs. Concurrent advances in dual‐targeting, iPSC‐derived platforms, and in vivo CAR‐T generation herald a new era of precision immunotherapy—one poised to overcome current limitations and deliver scalable, curative‐intent therapies to patients worldwide (Table 1).

**TABLE 1:** Clinical characteristics and treatment outcomes of published CAR‐T therapy clinical trials for autoimmune diseases.

Item	Autologous CAR‐T (Nature Med, 2022) [65]	Allogeneic (off‐the‐shelf) CAR‐T (CELL RES) [80]	Allogeneic (off‐the‐shelf) CAR‐T (N Engl J Med.) [84]
Patients/characteristics	5 patients (4F, 1M), median age 22 years, baseline SLEDAI‐2K 8–16	4 patients (all female), age 22–24 years, baseline SELENA‐SLEDAI 14–26, including lupus cerebritis	5 patients, baseline SLEDAI‐2000 8–24, with lupus nephritis
Treatment regimen	Lymphodepletion (fludarabine + cyclophosphamide), single infusion of 1 × 10^6^ CAR‐T/kg (ex vivo transduction)	Reduced‐intensity lymphodepletion (three out of four patients), single infusion of 1 × 10^6^ CAR‐T/kg (CRISPR‐edited)	No lymphodepletion, multiple repeated infusions (2–4 mg mRNA‐LNP, every 2–4 days)
Follow‐up period	Median 8 months (up to 20 months)	At least 6 months	At least 3 months
Safety highlights	Hospitalized monitoring for 10 days; only low‐grade CRS observed	Infection prophylaxis; no GvHD or ICANS observed	Low‐grade CRS; no chemotherapy‐related toxicity

### 4.2 Mechanisms of CAR‐T Cell Therapy in Autoimmune Neurological Disorders

Autoimmune neurological diseases, such as MS, are primarily characterized by autoimmune‐mediated nerve damage. MS and MG are disabling neurological diseases with a common immunological mechanism, with B cells playing a central role [85, 86]. In MS, B cells drive disease progression through various mechanisms, such as antigen presentation to T cells, production of proinflammatory cytokines, and local effects in the surrounding environment [87]. However, for most neuroimmune‐mediated diseases, including MS, specific pathogenic antibodies have not been identified. Currently, only two diseases have well‐defined pathogenic antibodies: MG associated with acetylcholine receptor (AChR) or muscle‐specific kinase antibodies and NMOSD associated with aquaporin‐4 (AQP4)–immunoglobulin G (IgG) antibodies [85]. These diseases lack a cure, and cell therapy strategies that “reset” the immune system, such as CAR‐T cell therapy, are emerging as future treatment options [88]. Therefore, CAR‐T cells, through deep B cell depletion strategies, can achieve long‐term immune tolerance in autoimmune neurological diseases, eliminating the need for continuous immunosuppressive treatments.

#### 4.2.1 Neuromyelitis Optica Spectrum Disorders

NMOSD is a central nervous system (CNS) autoimmune inflammatory disease characterized by acute bilateral optic neuritis or transverse myelitis (leading to severe vision loss and motor/sensory deficits) with a typical relapsing course. The pathogenesis of NMO is mainly related to demyelination and axonal damage mediated by AQP4 antibodies (AQP4–IgG). B cells and plasma cells produce anti‐AQP4 antibodies, leading to damage to astrocytes through ADCC and CDC effects, inducing autoimmune‐related demyelinating changes [89]. Given the remarkable ability of CAR‐T cells to eliminate B cell tumors, they can be used in the treatment of refractory NMOSD.

A tandem CAR‐T cell therapy targeting CD19 and CD20 (tanCART19/20) is being evaluated in a clinical trial (NCT03605238) to assess its safety and efficacy in treating NMOSD [90]. Additionally, an open‐label Phase I clinical trial (NCT04561557) initiated by Professor Wei Wang's team at Tongji Hospital, Tongji Medical College, Huazhong University of Science and Technology, uses BCMA‐specific CAR‐T cells to treat AQP4 antibody‐related NMOSD patients [91]. Twelve patients received BCMA CAR‐T cell therapy, with a median follow‐up of 5.5 months. A total of eleven patients (92%) did not experience NMOSD relapse. All 12 patients experienced Grade 1–2 CRS without any immune effector cell‐associated neurologic syndrome (ICANS). Improvements in vision (50%), walking ability (67%), and bladder/bowel functions (75%) were observed in the cohort. Corresponding improvements were also noted in other quality of life and pain scales.

#### 4.2.2 Myasthenia Gravis

MG is an acquired AID characterized by neuromuscular junction transmission impairment mediated by autoantibodies, primarily induced by anti‐AChR antibodies [92]. B cells play a crucial role in the pathological process of MG [93]. The Descartes‐08 therapy involves mRNA expression of CAR for CAR‐T preparation, enabling the transient rather than permanent expression of CAR molecules, thereby reducing the inherent risks of traditional CAR‐T cell therapy. Targeting BCMA, Descartes‐08 is utilized for treating generalized MG (gMG). Recently, Mozaffar et al. reported the results of a Phase Ib/IIa clinical trial using Descartes‐08 to treat gMG patients [94]. The first cohort of three gMG patients demonstrated significant disease improvement with good drug tolerance, without CRS or other SAEs. In the second cohort of 11 gMG patients with a median follow‐up of 5 months (range 3–9 months), there were no DLTs, CRS, or neurotoxicities. Clinical improvements in MG were significantly observed during a 9‐month follow‐up postinfusion. Additionally, Haghika et al. [95] treated a refractory MG patient using CAR‐T cells targeting CD19. During follow‐up, only a transient increase in transaminases occurred as an AE, with observed clinical improvements in Besinger's DAS and quantitative MG score. The patient was undergoing immunosuppressive treatment with planned cessation. Qin et al. [96] conducted a Phase I clinical trial using CAR‐T cell therapy targeting BCMA for the treatment of relapsed refractory NMOSD. The intermediate analysis results revealed that anti‐BCMA CAR‐T exhibited preliminary clinical efficacy and safety. Compared with other treatment modalities, CAR‐T cells have advantages in inducing direct target cell death, self‐amplification, and crossing the blood–brain barrier. Furthermore, they highlighted that BCMA CAR‐T cells with high CXCR3 expression effectively crossed the blood–brain barrier, eliminating plasma cells and inhibiting neuroinflammation in the cerebrospinal fluid (CSF). Notably, CAR‐T cells from NMOSD patients exhibited distinct cytotoxic inhibitory features compared with multiple myeloma patients receiving the same CAR‐T cell therapy, presenting innovative insights into the unique mechanisms of CAR‐T cell function in patients with neuroimmune AIDs.

#### 4.2.3 CAR‐T Therapy for MS

A recent study [97] reported the first use of fully human CD19‐targeted CAR‐T cell therapy (KYV‐101) for two patients with progressive MS. Developed by Kyverna Therapeutics, the therapy showed acceptable safety profiles in patients following administration, with the presence of CAR‐T cells detected in the patients’ CSF without clinical signs of NT. Both patients received a single dose of autologous CD19 CAR‐T cell therapy. Prior to this treatment, the two patients had ineffective responses to ocrelizumab and received fludarabine and CTX preconditioning before CAR‐T cell infusion. The first patient had SPMS for 23 years. Following CAR‐T cell infusion, the patient experienced Grade 1 CRS and elevated transaminases, with temporary exacerbation of disability. Although the EDSS score eventually returned to baseline levels, new spinal cord lesions were observed on MRI 2 months posttreatment. During follow‐up, a decrease in Ig production was noted in the patient's CSF. The second patient had primary progressive MS (PPMS) for 4 years. Following CAR‐T cell infusion, a transient increase in transaminases was the only AE observed during the follow‐up period, with stable EDSS scores and no decrease in Ig production in the CSF. Overall, this study strengthens the therapeutic potential of CAR‐T cell therapy for late‐stage MS patients with ineffective B cell depletion by traditional antibody‐mediated treatments. CD19‐targeted CAR‐T cell therapy can not only inhibit inflammation recurrence but also clear residual B cells in the CNS that may contribute to disease progression. Further evaluation of the short‐term and long‐term safety and efficacy of this therapy is necessary. CAR‐T therapy, as a promising novel approach, particularly in targeting B cells, such as the CD19 antigen present on virtually all B cells, holds significant clinical and economic benefits by selectively targeting and correcting defective immune systems, resulting in lasting efficacy and reduced reliance on long‐term immunosuppression. Several Phase 1/2 CAR‐T cell clinical trials targeting CD19‐positive cells for relapsing and progressive MS are currently underway. In a landmark update presented on June 23, 2025, IASO Bio announced that preliminary clinical data from an investigator‐initiated trial (IIT) [98]evaluating its BCMA‐targeted CAR‐T therapy, equecabtagene autoleucel, in patients with MS, were delivered as an oral e‐poster presentation at the 11th Congress of the European Academy of Neurology. As of December 31, 2024, three patients with severe progressive MS—two with SPMS and one with PPMS—had been enrolled. All exhibited high baseline disability, with EDSS scores ranging from 6 to 7, and had previously failed biologic therapies. Following a single infusion of equecabtagene autoleucel at a dose of 1.0 × 10^6^ cells/kg, all patients demonstrated rapid and sustained clinical improvement. At last follow‐up, each patient exhibited a significant reduction in EDSS score compared with baseline. Functional gains were evident: upper limb dexterity, as measured by the 9‐HPT, and lower limb ambulatory capacity, assessed via the T25‐FW, both markedly improved. CSF analysis revealed complete disappearance of oligoclonal bands (OCBs), accompanied by a substantial decline in kappa free light chain levels. Serial MRI evaluations showed no new or enlarging gadolinium‐enhancing T1 lesions or new T2 hyperintense lesions, indicating suppression of active CNS inflammation. Safety profiling revealed only transient Grade 1 CRS in all patients; no ICANS or other neurotoxic events were observed. All Grade ≥3 cytopenias occurred within 30 days postinfusion and were limited to neutropenia and lymphopenia; no Grade ≥3 anemia or thrombocytopenia was reported. In summary, equecabtagene autoleucel demonstrates a favorable safety profile and clinically meaningful efficacy in progressive MS, characterized by measurable improvements in motor function and the unprecedented clearance of CSF OCBs—a hallmark of intrathecal inflammation in MS. These findings support its potential as a disease‐modifying immunotherapy capable of resetting pathogenic humoral immunity in the CNS.

Further research is needed to validate the long‐term effects of CAR‐T cell therapy in autoimmune neurological disorders and to determine the optimal patient selection criteria. With advancing technology and ongoing clinical trials, CAR‐T cell therapy is poised to become a potent tool for treating such complex diseases. However, additional clinical trials and long‐term follow‐up studies are required to confirm its long‐term safety and effectiveness.

#### 4.2.4 Chronic Inflammatory Demyelinating Polyneuropathy

Chronic inflammatory demyelinating polyneuropathy (CIDP) is a chronic, progressive, or relapsing‐remitting neuropathy mediated by autoimmune responses targeting the peripheral nervous system [99]. First‐line therapies include IVIG, corticosteroids, or plasma exchange; however, approximately 15% of patients remain refractory to current treatment modalities [99]. Given the putative pathogenic roles of B cells and autoantibodies in CIDP, BCMA‐targeted CAR‐T cell therapy represents a rational therapeutic strategy. The study by Dong et al. [100] provides the first clinical evidence supporting the safety and therapeutic potential of BCMA‐directed CAR‐T cells in refractory CIDP, while also offering mechanistic insights into the molecular basis of disease remission. In this novel study, researchers from Tongji Hospital, Tongji Medical College, Huazhong University of Science and Technology, employed a BCMA‐targeted CAR‐T cell product developed by IASO Bio (Nanjing, China) to treat two patients with refractory CIDP. The therapy demonstrated a manageable safety profile in both cases: one patient experienced disease relapse at 12 months postinfusion, while the other achieved sustained clinical remission exceeding 24 months. To elucidate the molecular mechanisms underlying therapeutic efficacy and differential clinical responses, the team performed multiomics analyses on peripheral blood and CSF samples collected pre‐ and postinfusion, as well as on the infused CAR‐T cell product.

Both patients achieved drug‐free remission within 6 months following CAR‐T infusion and experienced no SAEs. Patient 1 relapsed at 12 months following SARS‐CoV‐2 infection, accompanied by reactivation of pathogenic B‐cell clones and reappearance of autoantibodies/peptides targeting axonal or myelin antigens. Metabolic reprogramming of B cells—characterized by hyperactive glycolysis—was associated with relapse and appeared to be regulated by the transcription factor RFX5. In contrast, Patient 2 maintained durable remission for over 24 months. Collectively, this study demonstrates the safety and therapeutic potential of BCMA‐targeted CAR‐T cell therapy in refractory CIDP and provides novel mechanistic insights into the immunological basis of disease control and relapse.

#### 4.2.5 Autoimmune Nodopathies (Autoimmune Node/Paranode Diseases)

Autoimmune nodopathies (ANs) constitute a spectrum of immune‐mediated peripheral neuropathies characterized by serum autoantibodies targeting the nodes of Ranvier and adjacent paranodal structures [101]. Key autoantibodies include those against neurofascin‐155 (NF155), contactin‐1, and contactin‐associated protein 1—collectively termed paranodal antibodies—and those against NF186/NF140, which target the nodal region. These antibodies are predominantly of the IgG4 subclass, and their specificities correlate with distinct clinical phenotypes [101]. AN may present with acute, subacute, or chronic onset, with progression typically exceeding 8 weeks, manifesting as a multifocal motor and sensory peripheral neuropathy [102]. Therapeutic options include corticosteroids, RTX, or plasma exchange; notably, IVIG demonstrates limited efficacy [103]. A landmark clinical report published in *The Lancet Neurology* by researchers at St. Josef Hospital, Ruhr University Bochum, Germany, presents the world's first application of CAR‐T therapy in severe autoimmune neuropathies [104]. The study included two patients: one with CIDP and one with AN. Patient 1, a 72‐year‐old male, was diagnosed in 2022 with a motor‐predominant CIDP variant. By 2024, despite multiple immunotherapies—including IVIG, corticosteroids, plasma exchange, CTX, RTX, obinutuzumab, and bortezomib—he became wheelchair‐bound and developed catheter‐related thrombosis, Staphylococcus aureus infection, and pulmonary embolism (PE). Patient 2, a 54‐year‐old male, presented after 2 years of progressive weakness and gait impairment. In 2023, he was diagnosed with anti‐NF155 IgG‐positive AN. Despite treatment with IVIG, plasma exchange, and RTX, he remained wheelchair‐dependent with persistent tremor. Both patients received a single infusion of anti‐CD19 CAR‐T cells in 2024. Peak CAR‐T expansion occurred on Days 8 and 14 postinfusion, respectively, accompanied by complete B‐cell depletion. Both experienced mild‐to‐moderate CRS, which was successfully managed with tocilizumab and corticosteroids. Clinically, both patients exhibited dramatic improvement. By 6 months, Patient 1 progressed from intermittent nonambulation to performing squats and pull‐ups. His Inflammatory Neuropathy Cause and Treatment–Overall Disability Sum Score (INCAT‐ODSS), walking distance, and serum NFL concentrations improved substantially. Anti‐GM1 IgM antibodies became undetectable by Month 10. Patient 2 regained the ability to walk independently for >200 m by Month 4; his INCAT‐ODSS improved from 7 to 4, serum NfL and tremor normalized, and anti‐NF155 IgG antibodies became undetectable. Distal compound muscle action potentials improved by up to 200% in both patients. All concomitant immunotherapies were successfully discontinued post‐CAR‐T infusion.

These findings indicate that anti‐CD19 CAR‐T therapy is a promising intervention for severe, refractory autoimmune neuropathies, capable of inducing sustained clinical remission without ongoing immunosuppression. The therapy was well tolerated; early toxicities, including CRS, were mild‐to‐moderate and manageable with standard interventions. Over a maximum follow‐up of 10 months, safety remained favorable; one patient developed hypogammaglobulinemia requiring periodic IVIG supplementation—consistent with prior reports of CAR‐T safety in autoimmune contexts. CAR‐T therapy may thus represent a single‐administration treatment with potential for long‐term remission. Larger, prospective, controlled trials are warranted to confirm efficacy, durability, and long‐term safety. Notably, the investigators report that 11 additional patients with severe autoimmune neurological disorders—including CIDP, MG, and stiff‐person syndrome (SPS)—have since been enrolled in their CAR‐T clinical program, heralding a potential paradigm shift in the management of these debilitating conditions.

#### 4.2.6 Antidiacylglycerol Lipase‐α Antibody‐Associated Autoimmune Encephalitis

Refractory antidiacylglycerol lipase‐α (DAGLA) antibody‐associated autoimmune encephalitis is a CNS inflammatory disorder mediated by autoantibodies targeting neuronal DAGLA, leading to neuroinflammation and functional impairment [105]. Clinical manifestations may include rapidly progressive generalized myoclonus, cerebellar head tremor, vertical gaze‐evoked nystagmus, quadriparesis, cognitive deficits, psychiatric symptoms, and seizures [105]. This condition is notoriously refractory to conventional immunotherapies—including corticosteroids, plasma exchange, and RTX—with disease progression often continuing unabated despite aggressive intervention, underscoring the urgent need for novel therapeutic strategies [106]. In a pioneering case report, Hegelmaier et al. [107] successfully treated a patient with refractory anti‐DAGLA encephalitis using CD19‐directed CAR‐T cell therapy (KYV‐101, a fully human, second‐generation construct). At 1‐year follow‐up, the patient maintained sustained clinical improvement. Clinical response was assessed using the International Cooperative Ataxia Rating Scale and the Clinical Assessment Scale in Autoimmune Encephalitis, while anti‐DAGLA antibody titers in serum and CSF were quantified via recombinant cell‐based indirect immunofluorescence and confirmed by staining of primary murine neurons and brain sections. The 36‐year‐old male patient had previously failed pulse corticosteroids, plasma exchange, and RTX, yet continued to deteriorate with rapidly progressive myoclonus, cerebellar tremor, vertical nystagmus, and quadriparesis. Anti‐DAGLA antibodies were confirmed positive in both serum and CSF, with specific binding to neuronal elements in tissue assays. Given the severity and treatment‐refractory nature of his disease, he received a single infusion of autologous anti‐CD19 CAR‐T cells. Posttreatment, clinical scores improved markedly, and anti‐DAGLA autoantibody levels in both serum and CSF declined significantly. Notably, CSF OCBs, initially positive, converted to negative following CAR‐T therapy—indicative of abrogated intrathecal humoral autoimmunity.

### 4.3 Mechanisms of CAR‐T Cell Therapy in Autoimmune Muscle–Skin System Diseases

#### 4.3.1 CAR‐T Therapy for SSc

SSc is characterized primarily by fibrosis of the skin and internal organs [108]. Antifibrotic therapy is key in managing the condition, and in recent years, CAR‐T cell therapy has shown progress in the antifibrotic domain. Bergmann et al. [109] reported a case of a 60‐year‐old SSc patient presenting severe skin hardening, myocardial fibrosis, pulmonary interstitial fibrosis, and pulmonary arterial hypertension, along with serum positivity for anti‐RNA Polymerase III antibodies. Following ineffective conventional treatments, this patient underwent CD19 CAR‐T cell therapy, and within 3–6 months of follow‐up, significant improvements were observed in the patient's cardiac, pulmonary, skin, and joint symptoms, along with a notable decrease in autoantibody titers. Merkt et al. [110] also documented a case of an SSc patient positive for Scl‐70 antibodies, with rapidly progressive interstitial lung disease (ILD). After the infusion of CAR‐T cells for 10 days, the patient experienced marked relief in cough and dyspnea symptoms, significant improvement in chest CT showing ground‐glass opacities, and confirmed regression of activated lung fibroblasts through Ga–FAPI–PET/CT imaging in the sixth month. In these studies, both patients experienced only Grade 1 CRS with no specific intervention. Müller et al. [66] similarly observed four SSc patients achieving comparable therapeutic effects post‐CAR‐T cell therapy. Notably, in the study by Merkt et al. [110], the third‐generation CD19 CAR‐T cell therapy resulted in Scl‐70 antibody seroconversion, with detectable CAR‐T cells even after 11 months of treatment; meanwhile, in the study by Müller et al. [66], although Scl‐70 antibodies decreased, seroconversion did not occur, and CAR‐T cells disappeared within weeks. These differences might be related to variations in CAR‐T cell structure and infusion dosages. However, the pathogenic mechanisms of Scl‐70 antibodies in SSc, the necessity of sustained presence of CAR‐T cells for antibody seroconversion, and the risks and benefits of sustained CAR‐T cell action over time and B‐cell depletion remain unclear, necessitating further exploration.

Recently, the team led by Georg Schett/Fabian Muller detailed the effects of autologous CD19 CAR‐T cell therapy on organ and skin manifestations of SSc [111]. From April 20, 2022 to November 8, 2023, the study enrolled six patients with severe diffuse SSc, with a median age of 42 years, including four males and two females, all of white European descent. The median disease duration (since the first non‐Raynaud's symptom) was 36 months. All patients had previously received immunosuppressive therapy. Over a median follow‐up time of 487 days in the study, organ manifestations of SSc did not deteriorate, with no new pulmonary, cardiac, or renal events, and an improvement in the ACR‐CRISS index was observed. Similar to other autoimmune connective tissue diseases like SLE, patients undergoing CD19 CAR‐T cell therapy experienced depletion of ANAs and a reduction in SSc‐specific autoantibodies. The disease course of SSc is characterized by alternating stages of progression and stability, unlike the flares and remissions seen in SLE. Safety and tolerability regarding CRS and infection rates in different connective tissue diseases are comparable. In this study and others investigating CD19 CAR‐T cell therapy for various connective tissue diseases, the incidence of CRS is relatively low compared with hematologic diseases, and no ICANS events were reported. The reasons for this phenomenon are not fully elucidated, but possible explanations include a lower load of CD19‐positive B cells in AIDs compared with B‐cell malignancies, along with the shorter duration of CD19 CAR‐T cell activity. Comparative studies on the safety and efficacy of CD19 CAR‐T cell therapy versus autologous stem cell transplantation with deep immune ablation, as well as with targeted CD20 B‐cell depletion therapy and other advanced treatments, along with longer follow‐up, will help clarify the role of CD19 CAR‐T cells in the current treatment landscape and better understand which patient subgroups benefit the most from each treatment modality.

Recently, this research team successfully used allogeneic UCAR‐T cell therapy to treat one patient with necrotizing myopathy and two SSc patients [112]. Through CRISPR–Cas9 gene editing technology, the team knocked out immune‐related genes like HLA‐A and HLA‐B from T cells sourced from healthy donors to create UCAR‐T cells targeting CD19. The results showed effective expansion of CAR‐T cells and complete B‐cell clearance in all three patients, with B‐cell reconstitution achieved 3 months posttherapy. Skin biopsies of diffuse SSc patients in the study indicated significant improvement in inflammation, restoration of hair follicle atrophy, and reduced sweat gland loss, demonstrating hair follicle regeneration and new gland formation. Imaging studies showed fibrosis reversal in vital organs like the heart and lungs, maintaining a state of continuous remission throughout the 6‐month follow‐up period.

*Cabaletta Bio's Rese‐cel in Systemic Sclerosis: Early Clinical Signals of Immune Reset*. In 2025, Cabaletta Bio reported preliminary clinical data from its ongoing Phase 1/2 RESET‐SSc trial (NCT06328777) evaluating rese‐cel (formerly CABA‐201)—a fully human, autologous, 4‐1BB costimulatory domain‐containing, anti‐CD19 CAR‐T cell therapy—in adult patients with SSc and severe cutaneous or organ involvement [113]. Designed for single weight‐based dosing, rese‐cel aims to induce transient yet profound depletion of CD19^+^ B cells, thereby facilitating immune system “reset” and potentially enabling durable clinical responses without long‐term immunosuppression. As of the reporting date, three patients had been enrolled. One patient, treated in the severe skin involvement cohort, completed 3 months of follow‐up; two additional patients had undergone leukapheresis and were scheduled to receive rese‐cel in early 2025. The first treated patient exhibited favorable tolerability, with no DLTs or ICANS. Grade 2 CRS, manifesting as fever and hypotension, was managed with intravenous fluids alone, without tocilizumab. The patient maintained a drug‐free response throughout the 3‐month follow‐up. Clinically, mRSS improved progressively from 42 at baseline to 34. Pulmonary function also improved: forced vital capacity increased from 91 to 97% predicted, and diffusing capacity for carbon monoxide rose from 70 to 85% predicted. Pharmacodynamic analyses revealed robust rese‐cel expansion, peaking at Day 15 (*C*_max_) and becoming undetectable by Day 29. The infused product was predominantly CD4^+^; however, at *C*_max_, a CD8^+^‐dominant phenotype emerged. Serum IFN‐γ peaked on Day 8—preceding *C*_max_—while interleukin‐6 (IL‐6) peaked concurrently with *C*_max_. Peripheral B cells were rapidly depleted, becoming undetectable by Day 15. B‐cell reconstitution, observed at 2 months postinfusion, was characterized by transitional (CD19^+^CD20^+^CD38^+^^+^CD24^+^^+^) naïve B‐cell phenotypes. Ultrasound‐guided core needle biopsy of the left inguinal lymph node further confirmed profound B‐cell depletion within lymphoid tissue. Immunohistochemical staining of baseline and Day 21 postinfusion biopsies revealed a mixed infiltrate of CD3^+^ T cells and CD19^+^/CD20^+^ B cells at baseline, whereas only CD3^+^ T cells remained detectable at Day 21—with no residual CD19^+^ or CD20^+^ B cells. In summary, data from the first patient with severe cutaneous SSc demonstrate early, immunosuppression‐free clinical response and favorable safety, coinciding with robust CAR‐T expansion and complete depletion of B cells in both peripheral blood and lymphoid tissue. These preliminary findings suggest that rese‐cel may facilitate immune reset in SSc, enabling meaningful clinical improvement without ongoing immunosuppressive therapy.

*Bristol Myers Squibb's BMS‐986353 (CC‐97540) in SSc: Preliminary Efficacy and Safety from Breakfree‐1 Trial*. At the 2025 EULAR Annual Meeting, Bristol Myers Squibb presented updated data from the ongoing Phase 1, multicenter, open‐label Breakfree‐1 trial (NCT05869955) evaluating BMS‐986353 (CC‐97540)—a CD19‐directed CAR‐T cell therapy utilizing the clinically validated lisocabtagene maraleucel (liso‐cel) construct, manufactured via the proprietary NEX‐T process to shorten production time and optimize CAR‐T cell product phenotype—in patients with srSLE, inflammatory myopathies, and SSc [114]. As of December 6, 2024, five SSc patients had received BMS‐986353 (four in DL1, one in DL2). All were evaluable for safety and efficacy. Median age was 51 years (range: 43–82) in DL1 and 41 years in DL2. Median follow‐up was 106 days (range: 32–173) in DL1 and 46 days in DL2. Time from diagnosis to infusion was 1.5 years (range: 1.3–2.7) in DL1 and 3.1 years in DL2. Patients had received a median of three prior therapies (range: 2–5 in DL1; *n* = 3 in DL2). At baseline, DL1 patients exhibited median PGA of 6.5 (range: 6.0–8.0), median Health Assessment Questionnaire–Disability Index of 1.8 (range: 1.1–2.8), and median mRSS of 24.5 (range: 4.0–42.0); DL2 values were 4.0, unavailable, and 7.0, respectively. Three DL1 patients had ILD (two diffuse, one limited); the DL2 patient had limited ILD. All five patients experienced treatment‐emergent AEs (TEAEs). One DL1 patient developed a Grade 3/4 TEAE (Grade 3 headache) deemed related to BMS‐986353. One DL1 patient experienced Grade 1 neutropenia. Three DL1 patients developed CRS (all Grade 1/2; median duration: 2.0 days); one DL1 patient experienced Grade 1 ICANS (duration: 3.0 days). No prolonged Grade ≥3 cytopenias, systemic infections, or DLTs were reported. Among DL1 patients with dcSSc (*n* = 3), mRSS reductions at last evaluable visit were 15, 13, and 0 points (limited follow‐up at Day 29 postinfusion). All DL1 patients achieved complete peripheral B‐cell depletion. In summary, BMS‐986353—leveraging the United States Food and Drug Administration (US FDA)‐approved liso‐cel backbone and optimized via NEX‐T manufacturing—demonstrates a manageable safety profile in severe refractory SSc. All CRS events were Grade 1/2, transient, and reversible; one Grade 1 ICANS event resolved rapidly. Preliminary efficacy signals are encouraging. Dose escalation continues across all cohorts to identify the recommended Phase 2 dose (RP2D) balancing optimal efficacy and safety per indication.

*Fate Therapeutics’ FT819: Off‐the‐Shelf iPSC‐Derived CD19 CAR‐T for AIDs*. Also at EULAR 2025, Fate Therapeutics presented early clinical data on FT819—a first‐in‐class, off‐the‐shelf, iPSC‐derived CD19 CAR‐T cell product candidate engineered from a clonal master iPSC line, enabling scalable, reproducible manufacturing for broad patient access [115]. FT819 is designed to enhance safety and potency through three key innovations: (1) a novel 1XX CAR signaling domain shown to prolong T‐cell effector function without exhaustion; (2) targeted CAR gene integration into the TRAC locus to ensure uniform CAR expression and enhanced functionality; and (3) complete biallelic disruption of the TCR to eliminate GvHD risk. Three patients received a single dose of FT819 (3.6 × 10^8^ cells) following fludarabine‐free lymphodepletion and were followed for 2–8 months. Median age was 28 years (range: 22–29). All had biopsy‐confirmed LN with renal BILAG A scores. Patients had received a median of eight prior therapies, including RTX; two had previously received CTX. At study entry, all continued hydroxychloroquine; two remained on low‐dose glucocorticoids. Lymphodepletion regimens consisted of CTX alone (*n* = 2) or bendamustine alone (*n* = 1).

All patients achieved rapid and profound peripheral B‐cell depletion. No FT819‐related SAEs, CRS, NT, or DLTs were observed. Clinical improvement was evident across all patients: reductions in SLEDAI‐2K, PGA, and FACIT‐Fatigue scores, accompanied by decreased proteinuria in LN patients and declining serum anti‐dsDNA antibody titers. The first patient, now at 6 months of follow‐up, achieved DORIS‐defined clinical remission and low lupus disease activity, and successfully discontinued glucocorticoids by Month 3. Collectively, initial data from the first three patients support the favorable safety profile, potent B‐cell depletion, and early clinical efficacy of FT819. Reconstituting B cells exhibited a predominantly naïve phenotype. These findings warrant continued evaluation of FT819 in SLE and expansion into other B‐cell‐mediated AIDs, including antineutrophil cytoplasmic antibody (ANCA)‐associated vasculitis (AAV), IIMs, and SSc.

#### 4.3.2 CAR‐T Therapy for Inflammatory Myositis

Antisynthetase syndrome (ASS) encompass a group of SRDs characterized by antibodies against synthetases, myositis, interstitial pneumonia, fever, arthritis, and mechanic's hands [116]. The likelihood of interstitial pneumonia is higher in this disease category compared with dermatomyositis, and the prognosis varies significantly based on different types of antisynthetase antibodies. For instance, ASS with anti‐EJ antibodies can exhibit severe acute respiratory distress syndrome [116]. Reports indicate that tacrolimus, a calcineurin inhibitor, shows some effectiveness in patients with ASS and interstitial pneumonia unresponsive to glucocorticoid therapy, achieving not only pulmonary symptom improvement but also glucocorticoid dose reduction [116]. In recent years, IVIG [117, 118], CTX, and RTX have demonstrated positive effects in the treatment of ASS combined with refractory interstitial pneumonia [119], with RTX showing superior long‐term efficacy over CTX [120]. Refractory interstitial pneumonia combined with ASS that is unresponsive to traditional ISs and RTX therapy poses a common challenging issue in clinical practice, necessitating exploration of novel treatment modalities.

Pecher et al. [121] reported a case of a patient with ASS and interstitial pneumonia who underwent CAR‐T immunotherapy targeting CD19. The patient received fluorouracil in combination with CTX as a pretreatment before CAR‐T cell infusion and was subsequently maintained on methotrexate therapy. Results showed rapid clinical improvement after CAR‐T cell infusion, with significant enhancements in clinical assessments, cytokine levels, and anti‐Jo‐1 antibody titers after 8 months of treatment. Similarly, Müller et al. [122] reported another case of refractory ASS patient who achieved similar efficacy with CAR‐T therapy following ineffective conventional treatments. Both patients had tried various ISs before CAR‐T therapy, with IVIG and RTX also proving ineffective. Even after treatment, the patients continued to exhibit severe muscle weakness and progressive respiratory difficulties. Both patients experienced Grade 1 CRS during CAR‐T therapy, with one receiving tocilizumab for management. Taubmann et al. [123] documented a case of a 44‐year‐old female ASS patient positive for anti‐Jo‐1, anti‐Pm‐Scl‐100 antibodies, and rheumatoid factors (RFs), who underwent CAR‐T therapy. The patient presented with recalcitrant myositis, arthritis, interstitial pneumonia, and skin manifestations. Despite being unresponsive to glucocorticoids, traditional ISs, tofacitinib, tocilizumab, IVIG, CD20‐targeted monoclonal antibodies (including first and second generations), and even the investigational cannabinoid receptor 2 agonist lenabasum, the patient showed only mild improvement. However, post‐CAR‐T infusion, significant efficacy was observed, with marked improvement in joint, muscle, and pulmonary symptoms, and minimal adverse reactions, limited to mild CRS. The patient remained in remission without medication for 5 months following CAR‐T infusion. Hence, for refractory ASS patients, especially those with aggravating interstitial pneumonia endangering life, CAR‐T immunotherapy may serve as a secondary treatment option after conventional therapies have failed.

In the realm of advances in UCAR‐T therapy, a recent study [112] utilized CRISPR–Cas9 gene editing technology to genetically modify CAR‐T cells targeting CD19 sourced from healthy donors, addressing the issue of immune rejection. This led to the development of a new generation of allogeneic UCAR‐T therapy (TyU19), which successfully treated one patient with refractory immune‐mediated necrotizing myopathy (IMNM) and two patients with dcSSc. The CAR‐T cells derived from healthy donor T cells were overall similar to the patients’ autologous CAR‐T cells. The research team isolated T cells from a 21‐year‐old female donor's peripheral blood mononuclear cells, transduced them with a lentivirus encoding anti‐CD19 CAR to create CD19‐targeted CAR‐T cells, utilized CRISPR–Cas9 gene editing to knock out five genes (HLA‐A, HLA‐B, CIITA, TRAC, and PD‐1) in the CAR‐T cells, and performed magnetic cell sorting to purify CD3‐negative cells to avoid graft‐versus‐host reactions caused by allogeneic T cells. Each batch could typically yield a quantity sufficient for >100 patients (with each infusion containing 1 × 10^6^/kg CAR‐positive cells).

During a 6‐month follow‐up after treatment, all three patients experienced significant alleviation of symptoms, marked improvement in disease Clinical Response Index scores, and reversal of inflammation and organ fibrosis. Remarkably, all three patients exhibited good tolerability throughout the treatment course, with no observed common AEs like CRS, GvHD, or ICANS typically seen in cancer patients undergoing CAR‐T cell therapy. These clinical study findings illustrate the significant potential of off‐the‐shelf universal allogeneic CAR‐T cell products in treating refractory AIDs. The new generation of UCAR‐T products utilized in this study offers several clinical advantages including extensive patient accessibility, higher clinical safety, and superior therapeutic efficacy, overcoming the challenges of highly personalized autologous CAR‐T cells such as long preparation cycles, high failure risks, and incredibly high costs, potentially shaping the direction of CAR‐T cell therapy development. The leader of this clinical trial, Xu, mentioned that 24 AID patients have also received this cell therapy, with overall positive treatment outcomes. These clinical results indicate that off‐the‐shelf UCAR‐T cells exhibit high safety and efficacy in treating severe refractory AIDs.

*CD19 CAR‐T in Pediatric MDA5^+^ DM with RPILD and MAS*. París et al. [124] recently reported a pediatric case of refractory MDA5^+^ dermatomyositis (DM) complicated by rapidly progressive ILD (RPILD), severe neurologic dysfunction associated with MAS, and progressive motor and pulmonary impairment. A single, fractionated dose of CD19 CAR‐T cells (ARI‐0001) induced progressive, drug‐free remission sustained for 11 months. Prior to CAR‐T infusion (Day 0), the patient's neurobehavioral status had normalized, indicating intact neurological function. From Day 0 to Day +22, CRS and ICANS were monitored thrice daily; no events occurred. On Day +31, the patient developed low‐grade fever and pulmonary consolidation, requiring hospitalization. *Pseudomonas aeruginosa* and rhinovirus were identified; symptoms resolved after 14 days of intravenous antibiotics. By Day +25, motor function, head control, and upper limb strength had fully recovered; lower limb strength continued to improve progressively. Physician Global Assessment and Childhood Health Assessment Questionnaire scores reflected satisfactory clinical remission. Muscle strength testing remained unfeasible due to residual neuropathy. Serial chest CT scans at Days +35, +113, and +325 demonstrated marked pulmonary improvement compared with baseline (Day −3), with reduced parenchymal opacities and fewer, smaller pulmonary cysts. Gottron's papules gradually regressed posthospitalization. Given the patient's poor short‐term prognosis and ineligibility for lung transplantation, compassionate use of second‐generation ARI‐0001 CD19 CAR‐T cells was approved following discontinuation of all immunosuppressive therapy. This represents the first reported case of CD19 CAR‐T therapy in pediatric MDA5^+^ DM‐RPILD following extracorporeal membrane oxygenation. Treatment yielded remarkable efficacy: skin, motor, and respiratory functions improved progressively over 11 months without immunosuppression. Although RTX administered on Day −112 achieved sustained peripheral B‐cell depletion, the expansion of CD19‐specific CAR‐T cells by Day +22 suggests that CD19^+^ B‐cell reservoirs persisted in deep tissues—potentially explaining the superior efficacy of CAR‐T over RTX, as it may target B‐cell subsets resistant to conventional therapies. B‐cell reconstitution began after Day +67, accompanied by reappearance of endogenous IgM and IgG—yet clinical improvement persisted throughout the immunosuppression‐free period (Days −7 to +325), supporting the hypothesis that elimination of autoreactive B‐cell clones underlies therapeutic efficacy. B‐cell repertoire analysis further corroborates this: 280 days post‐CAR‐T, B‐cell reconstitution was characterized by numerical recovery, maturation toward memory phenotypes, and causal correlation with rising IgM—without recurrence of autoimmunity, reinforcing successful immune reset.

However, CAR‐T therapy does not correct underlying genetic susceptibility; disease relapse remains possible. The team emphasizes ongoing close monitoring. For patients with complex, refractory AIDs and poor prognosis, profound B‐cell depletion may represent an innovative strategy to achieve sustained, drug‐free remission.

*Case of Relapsed IIM and Sequential CD19 → BCMA CAR‐T Rescue*. A recent study [125] describes a patient with idiopathic inflammatory myositis (IIM) who achieved 9 months of remission following initial CD19 CAR‐T therapy but subsequently relapsed. Readministration of CD19 CAR‐T failed to control disease, prompting successful rescue with BCMA‐targeted CAR‐T therapy. Initial CD19 CAR‐T infusion induced rapid, deep remission: normalization of creatine kinase (CK), improvement in manual muscle testing‐8 (MMT8) score, resolution of ILD, complete B‐cell depletion, and robust CAR‐T expansion. After 9 months, disease recurred. Upon reinfusion of CD19 CAR‐T, despite comparable lymphodepletion‐induced leukopenia, no CAR‐T expansion or B‐cell depletion occurred. To investigate resistance mechanisms, antidrug antibodies (ADA) and CAR‐reactive T cells were assessed. No anti‐FMC63 antibodies were detected prior to the second infusion; ADA emerged only 6 months later. However, peptide‐MHC multimer assays using 15‐mer epitopes revealed rapid emergence of CAR‐specific cytotoxic T cells immediately after reinfusion—suggesting that pre‐existing CAR‐reactive T cells eliminated CAR‐T cells before they could expand or deplete B cells, with ADA developing secondarily. Following CD19 CAR‐T failure, the patient received daratumumab (anti‐CD38)—motivated by persistent anti‐Jo‐1 antibody titers despite prior CD19 CAR‐T—aiming to deplete antibody‐secreting plasma cells. Clinical response was partial and transient, with disease progression by 5 months. This partial efficacy, however, provided rationale for BCMA CAR‐T therapy. BCMA CAR‐T infusion induced robust expansion and concomitant depletion of CD19^+^ B cells (reconstituting by Day 100). CK normalized within 3 months and remained stable. MMT8 scores improved by Week 3 and were sustained >9 months. Anti‐Jo‐1 titers declined. Serum IgG, IgM, and IgA decreased transiently, recovering by Day 100. Lymph node biopsy confirmed complete elimination of CD138^+^ plasma cells, while CD20^+^ B cells remained unaffected—implicating BCMA^+^ plasma cells as key drivers of disease progression. Initial response to CD19 CAR‐T likely reflected depletion of CD19^+^ plasmablasts and a subset of plasma cells. The patient has maintained remission for 9 months, with progressive reduction in arthritic inflammation. This case highlights the challenges of CAR‐T readministration and underscores the therapeutic potential of alternative targets. Most autoimmune CAR‐T therapies currently target CD19 or BCMA to eliminate autoantibody‐producing B‐lineage cells, primarily in antibody‐driven diseases (SLE, LN, MG, SSc, MS, NMOSD). However, CD19 expression is low or absent on plasma cells and LLPCs [126]. LLPCs, as persistent memory reservoirs, may survive CD19 CAR‐T therapy and—upon antigen re‐exposure—resume autoantibody production, leading to relapse [127], as evidenced by persistent anti‐Jo‐1 in this case.

Daratumumab, while effective in MM, shows limited efficacy in AID—likely due to inadequate tissue penetration and incomplete plasma cell depletion. The most advanced dual‐targeted CD19/BCMA CAR‐T in clinical development is iCell's cCAR [128]. Published data indicate durable, drug‐free remissions in most patients. However, one patient—due to severe marrow suppression from prior therapies—received an insufficient CAR‐T dose initially, achieving only 6 months of remission upon retreatment. Literature suggests initial failure was dose related; secondary failure likely resulted from anti‐CAR immunity. This pattern reinforces that readministration of identical CAR‐T products risks immune‐mediated rejection; switching targets or CAR constructs may offer superior outcomes.

*First Successful CD19 CAR‐T Therapy in Refractory Juvenile Dermatomyositis*. Nicolai et al. [129] reported the first successful application of anti‐CD19 CAR‐T therapy in a 12‐year‐old Caucasian boy with severe, refractory juvenile dermatomyositis (JDM). Diagnosed at the age of 6 years in Ukraine, the patient presented with symmetric proximal muscle weakness, elevated CK, and classic cutaneous manifestations (Gottron's papules, heliotrope rash, V‐sign, shawl sign). Despite multiple immunosuppressive regimens—including RTX—only partial, transient improvements in muscle strength, MRI findings, and skin involvement were achieved. Following CD19 CAR‐T infusion, progressive clinical improvement began at Week 4. By Week 34, the patient achieved Physician Global Assessment Visual Analog Scale (VAS) = 1/10, Childhood Myositis Assessment Scale = 50/52, and Cutaneous Assessment Tool–Binary Method activity score = 2/17—reflecting normalized muscle strength, near‐complete resolution of rash, marked reduction in calcinosis, and healing of chronic skin ulcers. At last follow‐up (8 months postinfusion), residual cutaneous lesions (minimal Gottron's signs on elbows/hands) continued to improve. Baseline CK was normal; however, persistent myositis was confirmed by muscle weakness and whole‐body MRI showing diffuse abnormal signal in axial/limb muscles and subcutaneous tissues, with extensive calcinosis. Postinfusion MRI at 3 and 6 months demonstrated resolution of myositis, marked reduction in subcutaneous signal abnormalities, and regression of calcinosis.

IFN‐related biomarkers mirrored clinical/radiological improvement. Preinfusion, the patient exhibited persistently elevated type I IFN scores—albeit with a declining trend despite prior therapies. Following CAR‐T infusion, IFN scores further decreased, normalizing by Week 24 and remaining stable. Serum CXCL9 and CXCL10, elevated pretherapy, progressively declined into the normal range from Week 24 onward. This represents the first documented case of successful CD19 CAR‐T therapy in JDM. Results suggest CD19 CAR‐T may be an effective therapeutic option for refractory JDM. The patient suffered from chronic, active disease with severe muscular and cutaneous involvement, resistant to multiple ISs—including RTX. In contrast, a single CD19 CAR‐T infusion induced complete peripheral B‐cell depletion through Week 8. Following discontinuation of immunosuppression, unprecedented clinical response emerged from Week 4: progressive resolution of myositis, normalization of strength, and marked skin improvement. Calcinosis persisted but substantially regressed. Despite B‐cell reconstitution beginning at Week 8—with mature B cells detectable by Week 18—residual skin manifestations continued to improve. As a single‐patient case, long‐term follow‐up and prospective trials are essential to validate the durability, safety, and broader applicability of profound B‐cell depletion via CD19 CAR‐T in JDM and other IIMs. These findings support the hypothesis that CD19‐targeted CAR‐T cells can induce sustained remission in refractory AIDs driven by autoreactive B cells, while avoiding the long‐term toxicities of chronic immunosuppression.

*Cabaletta Bio's Rese‐Cel in IIMs*. In 2025, Cabaletta Bio [130] reported interim data from its ongoing Phase 1/2 RESET‐Myositis trial (NCT06154252) evaluating rese‐cel (formerly CABA‐201)—a fully human, autologous, anti‐CD19 CAR‐T therapy designed for single weight‐based dosing to induce transient, profound CD19^+^ B‐cell depletion—in adult patients with IMNM, DM, and ASS, plus a fourth cohort of juvenile IIM patients. The therapy aims to achieve “immune reset” and durable, immunosuppression‐free remission. As of December 16, 2024, three patients (IMNM‐1, IMNM‐2, DM‐1) had completed ≥1 month of follow‐up; a fourth (IMNM‐3) had recently received infusion. Rese‐cel was well tolerated in the first three patients: no DLTs, CRS, or ICANS occurred. IMNM‐2 developed PE on Day 38, deemed unrelated to rese‐cel due to multiple genetic and clinical thromboembolic risk factors. DM‐1 exhibited rapid, profound response: achieved major Total Improvement Score (TIS) response by Day 29, sustained through Week 12, while discontinuing all therapies and tapering steroids. Cutaneous Dermatomyositis Area and Severity Index–Activity improved from 25 to 8 by Week 12. IMNM‐1 discontinued steroids shortly postinfusion, achieved clinically significant TIS by Week 12 and moderate TIS by Week 24, remaining therapy‐free through Week 40. IMNM‐2 showed declining CK by Day 29, but PE and prolonged hospitalization likely confounded subsequent efficacy assessment.

All three patients exhibited CAR‐T expansion, peaking between Days 8 and 15. CAR‐T cells became undetectable in peripheral blood by Day 29 in IMNM‐1 and IMNM‐2; detectable through Week 12 in DM‐1. Serum IFN‐γ was elevated preinfusion in IMNM‐1 and DM‐1; CAR‐T products shifted to CD8^+^‐dominant phenotype at peak. B cells were rapidly depleted to undetectable levels within 15 days postinfusion, remaining undetectable through Month 1. Naïve B‐cell reconstitution was observed by Week 8 in IMNM‐1 and DM‐1; B cells remained undetectable in IMNM‐2 through Week 8. In summary, early data from IIM patients demonstrate that rese‐cel can induce early, immunosuppression‐free clinical responses alongside robust CAR‐T expansion and profound peripheral B‐cell depletion, with a favorable safety profile. These preliminary findings suggest rese‐cel has the potential to reset the immune system in IIM, enabling significant clinical improvement despite discontinuation of all immunomodulatory therapies and glucocorticoid tapering.

*CASTLE Trial: Controlled Evaluation of CD19 CAR‐T in AIDs*. Friedrich‐Alexander‐Universität Erlangen‐Nürnberg and Universitätsklinikum Erlangen conducted CASTLE—a Phase I/II basket trial evaluating the safety (primary endpoint) and preliminary efficacy (secondary endpoint) of CD19 CAR‐T therapy (MB‐CART19.1; 1 × 10^6^ cells/kg after CTX/fludarabine lymphodepletion) in srSLE, IIM, and SSc [131]. An unplanned interim analysis included 20 patients (8 SLE, 8 SSc, 4 IIM) from Phases I (*n* = 8) and II (*n* = 16). Median age: 39.5 [27; 54.25] years; median disease duration: 3.75 [1; 6] years; median follow‐up: 7 [5.5; 12.25] months.

CAR‐T cells expanded in all patients (median peak: 146 [83; 360] cells/µL). Median B‐cell depletion duration: 84 [83; 360] days (*n* = 12 with B‐cell reconstitution). Circulating CAR‐T cells fell below 1 cell/µL by median Day 56 [35; 58], becoming undetectable by median Day 83 [56; 105] (*n* = 16). No high‐grade CRS (≥Grade 3; Grade 1: *n* = 13; Grade 2: *n* = 1) or ICANS occurred. Early neutropenia (any grade) occurred in 19 patients (95%) due to lymphodepletion; late Grade 3/4 neutropenia occurred in four patients (all SLE). Bone marrow biopsies (*n* = 2) showed no dysplasia or blasts. All cytopenias resolved. Three patients (one SLE, one IIM, one SSc) developed severe infections (all pneumonia, none ICU‐requiring). At 24 weeks (*n* = 15 evaluable), all seven SLE patients achieved DORIS remission; both IIM patients achieved ACR moderate/significant response; all six SSc patients showed stable/improved lung function and reduced skin scores. Critically, all patients remained off ISs and glucocorticoids for their underlying disease post‐CAR‐T. These data demonstrate the safety of CD19 CAR‐T in autoimmunity—with no high‐grade CRS/ICANS or persistent marrow toxicity—and encouraging preliminary efficacy across SLE, IIM, and SSc.

In a companion report [125], the team described a 45‐year‐old woman with refractory Jo‐1^+^ ASS (myositis, arthritis, recurrent fever, progressive ILD). She initially responded to CD19 CAR‐T but relapsed at 9 months. Reinfusion of cryopreserved autologous product failed due to lack of CAR‐T expansion and detection of anti‐CAR‐reactive T cells—likely mediating acute rejection. Despite full‐dose lymphodepletion, no clinical response occurred. Subsequent daratumumab (nine doses) induced partial, nondurable response. CMV viremia occurred, resolved with valganciclovir. Due to persistent disease activity, she received BCMA CAR‐T therapy. Grade 1 CRS occurred on Day 3, resolved with single‐dose tocilizumab. No NT or hematologic toxicity; no CMV reactivation under letermovir prophylaxis. BCMA CAR‐T expanded, depleted circulating B cells and lymphoid plasma cells, reduced autoantibodies, and reinduced stable, drug‐free remission within 3 months, with CK normalization. These data indicate: (i) In AID relapsing after initial CAR‐T, switching targets can restore drug‐free remission; (ii) retreatment with identical CAR‐T products risks immune‐mediated rejection; (iii) lymphodepletion alone, without CAR‐T expansion, exerts limited immunosuppressive effect in AID.

#### 4.3.3 CAR‐T Therapy in SPS

SPS is a rare AID characterized by progressive muscular rigidity and spasms, most commonly associated with autoantibodies against glutamic acid decarboxylase (GAD) or amphiphysin [132]. Despite available B‐cell‐targeted therapies—including plasma exchange, IVIG, anti‐CD20 monoclonal antibodies, and ISs—many patients remain refractory to treatment [133]. A recent landmark case reported by Kyverna Therapeutics [134] demonstrates the clinical and immunological impact of CD19‐directed CAR‐T therapy in refractory SPS. The patient, a 69‐year‐old woman with a 9‐year history of SPS, had failed multiple prior therapies, including RTX and bortezomib. She received a single infusion of autologous, second‐generation anti‐CD19 CAR‐T cells (KYV‐101; Kyverna Therapeutics), targeting CD19—a surface antigen expressed on B cells and their precursors. Following CAR‐T infusion, the patient exhibited marked clinical improvement. Leg rigidity substantially diminished, with her right knee Modified Ashworth Scale score declining from 2–3 to 0 within 14 days. Gait and ambulatory capacity improved dramatically: walking speed increased by >100%, and daily walking distance expanded from <50 m to >6 km within 3 months. Notably, her requirement for GABAergic medications (benzodiazepines) was reduced by 40%.

Immunological assessment revealed declining titers of anti‐GAD65 antibodies in both serum and CSF, suggesting suppression of pathogenic autoantibody production. CAR‐T cells expanded robustly, peaking at 56.7% of all CD3^+^ T cells by Day 14. Intriguingly, during CAR‐T expansion, the patient exhibited an initial rise followed by a decline in CD8^+^ T‐cell frequency, with a subsequent rebound beginning at Day 42—resulting in a dynamic shift in the CD4/CD8 ratio. Safety‐wise, anti‐CD19 CAR‐T therapy was well tolerated, with only low‐grade CRS and transient, mild elevation of hepatic enzymes observed. No SAEs (e.g., Grade ≥3 CRS or ICANS) occurred. This case adds to the growing body of clinical evidence supporting the use of anti‐CD19 CAR‐T therapy in CNS‐affecting AIDs. Prior studies have demonstrated efficacy in SLE, ASS, SSc, and MG. The successful application of anti‐CD19 CAR‐T cells in this severe, treatment‐refractory SPS case underscores the potential of this immunological strategy in B‐cell‐mediated AIDs.

While larger, controlled clinical trials are needed to fully evaluate the safety and efficacy of anti‐CD19 CAR‐T therapy in SPS and other neuroimmunological conditions, this report provides encouraging preliminary evidence to support further exploration of this promising therapeutic modality.

Notably, Kyverna Therapeutics anticipates several pivotal milestones for KYV‐101 in 2026. Like Bristol Myers Squibb's approach, KYV‐101 targets CD19 to eliminate pathogenic immune cells, thereby achieving what Kyverna terms “immune reset” in AID. KYV‐101 is uniquely engineered to mitigate CRS and enhance tolerability. The therapy is currently being evaluated in three indications: SPS, MG, and LN. SPS is the most advanced indication, with Phase II enrollment expected to conclude by mid‐2025 and top‐line results anticipated in early 2026.

#### 4.3.4 CAR‐T Therapy for Pemphigus Vulgaris

Pemphigus vulgaris (PV) is an autoimmune blistering disease characterized by autoantibodies targeting desmoglein 1 and desmoglein 3 (Dsg3), leading to blister formation and ulceration on the skin or mucous membranes [135]. Recently, Ellebrecht et al. designed a CAAR‐T cell therapy targeting B cells producing Dsg3‐specific autoantibodies. In vitro, Dsg3‐CAAR‐T cells exhibited specific toxicity against B cells expressing Dsg3 BCR, reducing Dsg3 autoantibody titers. Additionally, studies in a PV mouse model demonstrated that Dsg3–CAAR‐T cells could persist in vivo and target and suppress Dsg3‐specific B cells [136]. Data from a Phase I clinical trial (NCT04422912) of Dsg3–CAAR‐T cells for mucosal PV patients are pending. This Dsg3–CAAR‐T cell therapy aims to selectively target and eliminate B cells producing Dsg3 antibodies while preserving healthy B cells crucial for immune functions. Further research and clinical trials will help evaluate the potential of CAAR‐T in treating PV.

### 4.4 Application of CAR‐T Immunotherapy in Primary SS

pSS is a prototypical SRD characterized by B cell activation and aggregation in target organs, with a close association with lymphoma [137]. A predictive model study suggests that persistent salivary gland swelling and cryoglobulinemic vasculitis contribute to predicting the risk of lymphoma in pSS patients [138]. A large cohort study of primary pSS with lymphoma suggests that in the diagnosis of primary pSS, a composite index formed by cryoglobulinemia, focal lip biopsy lymphocytic foci, and SS disease activity index is an independent predictor of mucosa‐associated lymphoma [139]. Currently, conventional slow‐acting antirheumatic drugs have not been definitively proven effective in treating pSS. The efficacy of CD20 monoclonal antibody (RTX) in lymphoma suggests a potential benefit in treating pSS, but two large randomized controlled trials show limited benefits of RTX in pSS treatment [140, 141]. Combining the effectiveness of CAR‐T therapy in lymphoma treatment with the pathogenesis of SS leads to the prediction that CAR‐T immunotherapy might be effective in treating SS. When SS is combined with lymphoma, CAR‐T seems to be a suitable treatment option based on its mechanism of action. Sheng et al. [142] reported a case of a 76‐year‐old female pSS patient with concomitant diffuse large B‐cell lymphoma. After receiving CD19‐targeted CAR‐T therapy, the lymphoma achieved complete remission. Furthermore, after 10 years of pSS, following CAR‐T treatment, the patient seroconverted for antinuclear and anti‐Ro‐52 antibodies for the first time, and notably, 6 months after CAR‐T immunotherapy, the patient remained in complete remission with normalized serum cytokine levels, improved dry mouth symptoms, and a significant decrease in SS disease activity index, demonstrating the promising efficacy of CAR‐T therapy in SS. This patient experienced Grade 2 CRS and Grade 1 NT early in CAR‐T treatment, which were completely controlled with active intervention, illustrating the safety and promising application of CAR‐T immunotherapy in the diagnosis and treatment of SS. Currently, two Phase I clinical trials targeting CD19/BCMA with CAR‐T therapy for pSS are ongoing, focusing primarily on efficacy and safety, with results pending publication.

In terms of novel therapeutic targets, Ma et al. [143] further demonstrated that PD‐1+CD8+ tissue‐resident memory T cells, which are specifically enriched in the labial salivary glands of patients with Sjögren's disease (SjD), may be chronically stimulated by glandular antigens, thereby mediating glandular damage and driving disease progression. The authors engineered a CAR‐T cell therapy targeting the PD‐1 molecule and found that it rapidly depleted PD‐1+CD8+ T cells in both peripheral circulation and glandular tissues. This intervention effectively ameliorated xerostomia‐like symptoms in SjD mouse models and promoted structural and functional restoration of salivary gland tissue. This discovery not only provides a novel, precision‐based therapeutic strategy for SjD—a disease currently lacking effective treatments—but also offers critical mechanistic insights into the pathogenesis of AIDs.

### 4.5 Application of CAR‐T Immunotherapy in RA

Disorders in T cell immune tolerance play a major role in the pathogenesis of RA, highlighting the significance of restoring immune tolerance to control disease activity in RA. However, current traditional treatments are largely ineffective in restoring immune tolerance. CAR‐T cells are T lymphocytes engineered to express specific receptors on their membrane, enabling them to recognize specific antigens on target cells without being restricted by the MHC, thus interacting with target cells [144]. A study investigating CAR‐T therapy for RA targeted four citrullinated peptide epitopes, including citrullinated fibrinogen, citrullinated type II collagen, citrullinated fibronectin, and cyclic citrullinated peptide (CCP)‐1, as ligands for targeting autoreactive B cells. They constructed CAR‐T cells expressing a fixed antifluorescein isothiocyanate isomer I (FITC) CAR and demonstrated that the specifically engineered anti‐FITC CAR‐T cells successfully identified corresponding FITC‐tagged citrullinated peptide epitopes ex vivo and had the ability to eliminate RA patients’ specific autoreactive B cells by recognizing the FITC‐labeled self‐antigen peptide epitopes. This study showed that the specifically modified anti‐FITC CAR‐T cells represent a novel approach for treating RA [145].

Various types of autoimmune reactions exist in the pathogenesis of RA. If CAR‐T therapy acts only on a single type of B cell, its application in RA is limited. Immunotherapy targeting specific antigens has long been a goal in the treatment of AIDs, but developing a therapy targeting autoreactive T cells in an antigen‐specific manner poses certain difficulties. Whittington et al. conducted a study using CD8+ CAR‐T therapy targeting CD4+ T cell surface HLADR1 as the target antigen in a mouse model of autoimmune arthritis [60]. The results demonstrated the cell specificity of CD8+ CAR‐T therapy, accompanied by the suppression of autoantibody production in the model mice. Additionally, the incidence and severity of autoimmune arthritis in the model mice were reduced. CAR‐T therapy, whether targeting autoreactive B cells or autoreactive CD4+ T cells, contributes to the restoration of immune tolerance, offering a promising new approach to control the progression of RA. Currently, there are no reported cases of CAR‐T therapy applied in clinical treatment for RA patients, as severe visceral involvement in RA is relatively rare, leading to a lower benefit‐to‐risk ratio of CAR‐T therapy in RA patients. This might be one of the factors influencing its implementation in RA patients.

A recent landmark study [146] reported the first‐in‐human application of fourth‐generation CAR T cells for the treatment of refractory RA. The defining feature of fourth‐generation CAR‐T cells, distinguishing them from earlier generations, lies in their engineered capacity to secrete immunomodulatory cytokines or cytokine‐neutralizing antibodies upon activation, in addition to delivering canonical TCR‐like activation signals. This design was specifically conceived to mitigate excessive T‐cell activation and the consequent risk of cytokine‐driven inflammatory toxicity. In this trial, three female patients with clinically refractory RA received a single infusion of autologous fourth‐generation anti‐CD19 CAR‐T cells at a dose of 1 × 10^6^ cells/kg following standard lymphodepleting conditioning. Robust in vivo expansion of CAR‐T cells was observed in all three patients. Peripheral B cells were rapidly depleted within 3–7 days postinfusion and subsequently reconstituted by Day 60. Clinically, patients exhibited rapid and substantial improvements in joint swelling and tenderness, accompanied by marked reductions in systemic inflammatory markers—including erythrocyte sedimentation rate and C‐reactive protein (CRP). By Month 3, two patients achieved clinical remission, and one attained low disease activity; notably, all were able to discontinue their prior immunosuppressive regimens. At 6 months posttreatment, serum RF became undetectable in all patients. Anti‐CCP antibodies turned negative in two patients, while the third exhibited a 90% reduction in anti‐CCP titer compared with baseline. Imaging assessments further confirmed significant amelioration of synovitis relative to pretreatment levels. Regarding safety, no CAR‐T cell‐associated AEs, such as CRS or NT, were observed within 14 days of infusion. One patient developed a SARS‐CoV‐2 infection within 1 month posttreatment but fully recovered following antiviral therapy. This study represents the world's first clinical deployment of fourth‐generation anti‐CD19 CAR‐T cells in refractory RA. Although still exploratory and requiring longer‐term follow‐up to fully assess durability and safety, these preliminary results demonstrate compelling efficacy and a favorable safety profile, heralding a promising therapeutic avenue for refractory RA. Looking ahead, this engineering paradigm may be extended to other autoimmune conditions—such as SLE and SSc—potentially revolutionizing the therapeutic landscape for a broad spectrum of immune‐mediated diseases. Haghikia et al. [147] reported a case of CD19‐directed CAR‐T cell therapy in a female patient with severe, treatment‐refractory RA. The 39‐year‐old woman, who had a 20‐year history of erosive, seropositive RA, exhibited an inadequate response to all available biologic and targeted synthetic disease‐modifying antirheumatic drugs, resulting in severely impaired activities of daily living. Notably, while her peripheral blood B‐cell counts were persistently low, synovial biopsy revealed a substantial infiltration of CD19+ B cells (>70%). In light of this compartmentalized B‐cell pathology, the clinical team opted for CD19 CAR‐T cell therapy. The patient received an infusion of 1 × 10^6^ anti‐CD19 CAR‐T cells per kilogram of body weight, following lymphodepletion with fludarabine (75 mg/m^2^) and CTX (900 mg/m^2^). The CAR‐T cells were manufactured using the FMC63‐28‐CD3ζ construct. On Day 2 postinfusion, she developed Grade 3 CRS, which was followed on Day 5 by Grade 4 ICANS. These toxicities were successfully managed with tocilizumab, anakinra, and high‐dose corticosteroids. By Day 100, the patient had achieved sustained, drug‐free remission, with her DAS in 28 joints using CRP (DAS28–CRP) decreasing to 2.5. She exhibited no residual neurological deficits, and imaging studies alongside normalization of inflammatory markers confirmed significant clinical and radiological improvement. This case aligns with emerging reports of profound and sustained responses to CD19 CAR‐T therapy in polyrefractory RA.

### 4.6 CAR‐T Cell Therapy for Other AIDs

#### 4.6.1 CAR‐T Cell Therapy for T1D

T1D is an AID characterized by T cell‐mediated destruction of pancreatic B cells, influenced by factors such as autoimmunity, genetics, and viral infections [148]. Two types of CAR‐T cells are used for treating T1D, one of which targets and eliminates self‐reactive cells involved in B cell destruction. The primary self‐antigen epitope in nonobese diabetic (NOD) mice is situated between residues 9–23 of the insulin B chain, which, upon binding to NOD MHC II molecules (I‐Ag7), forms a pathogenic complex I‐Ag7‐B:9‐23 (R3)[148]. In 2019, Zhang et al. [149] introduced the monoclonal antibody mAb287 targeting this pathogenic complex into a CAR structure, creating 287‐CAR, and developed cytotoxic 287‐CAR‐T cells. These cells selectively bind to the pathogenic complex R3, delaying or preventing T1D. Although the exact mechanism of action of mAb287 remains unclear, it is speculated that it may be related to the depletion of R3‐presenting APCs by 287‐CAR‐T CD8+ cells. In 2020, Kuhns et al. from the University of Arizona reported a five‐module biomimetic CAR (5MCAR) targeting self‐reactive CD4+ T cells in a T1D mouse model [150]. The 5MCAR‐T cells can eliminate pathogenic self‐reactive T cells, preventing and treating T1D in mice. These cells were observed to persist in vivo for up to a year in treated mice. There are reports of conducting a study on the safety, preliminary efficacy, and cellular PK of a UCAR‐T cell injection targeting CD7 for the treatment of T1D.

#### 4.6.2 CAR‐T Cell Therapy in IgG4‐Related Disease

IgG4‐related disease (IgG4‐RD) is a chronic, immune‐mediated fibroinflammatory disorder capable of affecting multiple organs and systems [151]. Pathogenetic studies reveal that B cells, T cells, and macrophages play pivotal roles in disease initiation and progression [152]. In IgG4‐RD patients, B cells—particularly circulating plasmablasts and IgD^−^CD27^−^ double‐negative B cells—undergo oligoclonal expansion, infiltrate tissues, and secrete profibrotic mediators. Aberrant activation of B cells and plasmablasts constitutes a central driver of IgG4‐RD pathogenesis, positioning B‐cell depletion as a key therapeutic strategy. Although monoclonal antibody‐based B‐cell depletion therapies have demonstrated clinical promise, their efficacy is often limited by treatment dependency and high relapse rates, precluding sustained remission. Thus, there is an urgent need for more durable and innovative B‐cell‐targeted interventions [153]. Sun et al. [154] successfully engineered murine anti‐CD19 CAR‐T cells incorporating either CD28 or 4‐1BB costimulatory domains. These CAR‐T cells exhibited specific activation, robust proliferation, and potent cytotoxicity against target cells in vitro. In the Lat mouse model of IgG4‐RD, CD28‐based CAR‐T cells achieved B‐cell depletion comparable to that of anti‐CD19 monoclonal antibody therapy. Moreover, adoptive transfer of CD28 CAR‐T cells significantly reduced the frequencies of plasma cells and Th subsets secreting IL‐4 and TGF‐β, thereby attenuating both inflammation and fibrosis. Compared with 4‐1BB–based CAR‐T cells, CD28 CAR‐T cells demonstrated superior therapeutic efficacy without inducing severe CRS. The study further validated the feasibility of generating human CAR‐T cells ex vivo from IgG4‐RD patients; patient‐derived CAR‐T cells exhibited specific cytotoxicity and effectively suppressed the differentiation of profibrotic immune subsets. Collectively, these findings demonstrate that anti‐CD19 CAR‐T cells exert potent therapeutic effects with favorable safety profiles in murine IgG4‐RD models, highlighting their clinical potential as a novel therapeutic modality. Future optimization of CAR architecture and treatment regimens may further enhance efficacy and broaden applicability across rheumatic diseases.

Clinically, the rheumatology team led by Prof. Dong Lingli at Tongji Hospital, Tongji Medical College, Huazhong University of Science and Technology, recently initiated an investigator‐sponsored clinical trial evaluating BCMA‐targeted CAR‐T therapy in patients with recurrent or refractory IgG4‐RD [155]. Six‐month follow‐up data from the first two treated patients revealed marked reductions in IgG4‐RD RI scores. Radiographic assessments demonstrated significant regression—or even complete resolution—of disease‐associated masses. Laboratory analyses showed normalization of serum IgE and IgG4 levels, and VAS scores indicated substantial improvement in quality of life. These therapeutic benefits remained sustained through the 6‐month follow‐up period, with neither patient requiring additional immunosuppressive or supportive therapies post‐CAR‐T infusion.

#### 4.6.3 CAR‐T Cell Therapy in ITP

ITP frequently occurs secondary to a spectrum of AIDs. Patients with refractory thrombocytopenia face heightened risks of life‐threatening visceral hemorrhage and poor prognoses. Autoantibodies produced by B cells and plasma cells—including LLPCs—are considered the root pathogenic drivers of ITP. iCell Gene Therapeutics conducted a Phase I, open‐label, single‐arm, single‐center trial (IRB #2023‐1610) to evaluate the safety and efficacy of a dual‐target (BCMA/CD19) CAR‐T cell therapy in Chinese patients with refractory thrombocytopenia. Following lymphodepletion with CTX (500 mg/m^2^/day), patients received CAR‐T cells at a dose of 0.5–1.0 × 10^6^ cells/kg. Efficacy was assessed via platelet counts and antiplatelet antibody titers. AEs, including CRS and NT, were meticulously documented. CAR‐T cell kinetics were monitored by flow cytometry.

Between August and December 2024, four patients with AID‐associated refractory thrombocytopenia received dual‐target CD19/BCMA CAR‐T therapy: one with SLE and three with undifferentiated connective tissue disease. Preliminary safety, PK, and efficacy data are available for the first three treated patients; safety data are available for all four. All patients were female, aged 32–48 years, and had previously failed multiple lines of therapy, including high‐dose corticosteroids, hydroxychloroquine, ISs (cyclosporine, MMF, tacrolimus), IVIG, biologics (RTX, telitacicept, belimumab), and thrombopoietin receptor agonists. Bone marrow biopsies confirmed immune‐mediated thrombocytopenia and excluded clonal hematopoiesis (CHIP).

Following CAR‐T infusion, all four patients experienced transient, lymphodepletion‐associated cytopenias (Grade 3–4), as anticipated. Grade 3 neutropenia occurred in all patients but resolved within 2 weeks with supportive care. CRS was observed in all four patients; all events were Grade 1, with two patients receiving tocilizumab. No ICANS or infectious complications were reported. Remarkably, platelet counts in all patients normalized (>100 × 10^9^/L) postinfusion. Although Patient P2's platelet count initially normalized, it dipped slightly below the normal range by Day 42. Complete B‐cell depletion (0 cells/µL) was observed between Days 4–11, followed by gradual B‐cell reconstitution; Patient P1 exhibited detectable B‐cell recovery by Day 84. Nadir B‐cell levels occurred between Days 7–28, with recovery initiating by Day 56. CAR‐T cell expansion was confirmed via flow cytometry, with a median peak concentration (*C*_max_) of 806 cells/µL, reached between Days 7 and 14 postinfusion (*T*_max_). Preliminary data from these first four patients indicate that this novel dual‐target BCMA/CD19 CAR‐T therapy exhibits a favorable safety profile and promising efficacy. The rapid and robust platelet recovery observed in all patients by Day 14 postinfusion is particularly encouraging. To date, no severe toxicities have been reported [156].

#### 4.6.4 CAR‐T Cell Therapy in AAV

AAV is characterized by a breakdown in B‐cell tolerance, leading to pathogenic ANCA production, which in turn activates neutrophils and monocytes, culminating in systemic organ damage [157]. Although anti‐CD20 therapy (e.g., RTX) is clinically effective, up to 30% of patients exhibit primary treatment failure, and over 40% experience disease relapse—often necessitating lifelong immunosuppression. Notably, some patients relapse within 18 months despite adherence to standardized maintenance regimens. Alternative therapeutic modalities, such as anti‐CD19 CAR‐T cell therapy, may offer transformative clinical benefit for refractory cases [158]. In a landmark case presented at the 2025 EULAR by Charité—Universitätsmedizin Berlin [159], a 52‐year‐old male with a 20‐year history of severe, treatment‐refractory PR3‐ANCA^+^ AAV—manifesting multisystem involvement including pulmonary, renal, articular, cutaneous, sinonasal, and ocular disease—was treated with anti‐CD19 CAR‐T cells. At baseline, he presented with fever, weight loss, myalgia, arthralgia, exertional dyspnea, and productive cough (BVAS score: 6), despite repeated RTX infusions over the preceding decade. Circulating CD19^+^ B cells were profoundly depleted, yet PR3‐ANCA titers remained elevated (121.1 AU/mL). Reinduction therapy with RTX and avacopan partially alleviated dyspnea and stabilized weight, but persistent myalgia and arthralgia remained (BVAS: 2). Due to recurrent disease activity, the patient received an infusion of 1 × 10^8^ autologous anti‐CD19 CAR‐T cells following lymphodepleting conditioning with CTX and fludarabine. CAR‐T cells rapidly expanded in vivo, peaking at Day 14 (44.48% of CD3^+^ T cells) before gradually declining over 6 weeks. The patient experienced Grade 1 CRS, managed successfully with three doses of tocilizumab, and Grade 3 neutropenia, which resolved promptly with filgrastim support. No ICANS, infections, or other significant toxicities were observed. Clinically, the patient achieved complete symptom resolution, with stabilization of granulomatous lesions and a BVAS score of 0 by Day 132 postinfusion. CD19^+^ B cells were undetectable by Day 7 and began to reconstitute by Month 6. At Month 7, the patient remained asymptomatic (BVAS: 0) and off all immunosuppressive therapy—despite full B‐cell recovery—demonstrating durable clinical remission independent of ongoing B‐cell depletion. Key clinical insight: anti‐CD19 CAR‐T cell therapy represents a safe and potentially curative strategy for the subset of AAV patients with relentless relapses and resistance to RTX. This case underscores the capacity of CAR‐T therapy to reestablish immune tolerance and induce sustained, treatment‐free remission—even after B‐cell reconstitution—challenging conventional paradigms of B‐cell depletion as the sole mechanism of efficacy. These findings warrant further investigation in controlled clinical trials to define optimal patient selection, long‐term durability, and mechanistic correlates of response.

### 4.7 Advancements in CAR–Treg Therapy for AIDs

In this context, using Tregs as the raw material for CAR‐T cells might present a safer approach since Tregs lack cytotoxicity and are capable of suppressing excessive immune responses via the expression of inhibitory factors like CTLA4, secretion of IL‐10, and TGF‐Beta. Dysfunction in the quantity and function of Tregs in AIDs leads to impaired immune tolerance, immune dysregulation, inflammation, and the emergence of autoimmune cells [160]. Introducing functionally normal Tregs into the autoimmune milieu can potentially reestablish immune tolerance and ameliorate the disease [161]. However, nonspecific Tregs pose certain challenges, potentially leading to nonspecific immune tolerance, impacting their immune responses, and increasing the risk of cancer for patients [162, 163]. Additionally, in certain immunological environments, nonspecific Tregs have the potential to differentiate into proinflammatory Th17 cells. Therefore, the development of specific CAR–Tregs holds promise in mitigating the risk of systemic immune suppression, enhancing the precision and efficacy of CAR–Tregs, and reducing off‐target effects. Key advantages of CAR–Tregs include being independent of HLA limitations, heightened specificity through coreceptor signaling, and possessing target flexibility [164]. Studies have demonstrated in murine models that CAR–Tregs can aggregate at target antigen sites and elicit bystander suppression effects, indicating that while Tregs are activated by a specific antigen, they can inhibit the activity of T cells targeting other antigens in close proximity. This phenomenon holds promise for reconstructing immune tolerance near cells not expressing the target antigen, thereby reducing immune damage to affected organs [165].

#### 4.7.1 CAR–Treg Therapy for Active Inflammatory Bowel Disease

Active inflammatory bowel disease (IBD), encompassing ulcerative colitis and Crohn's Disease (CD), represents a group of chronic nonspecific intestinal inflammatory disorders where the pathogenesis of IBD is associated with dysregulated mucosal immune responses to gut microbiota [166]. Tregs function to modulate the intensity and duration of immune responses by inhibiting immune cell activity, thereby maintaining immune equilibrium. Increasing the quantity or enhancing the function of Tregs can alleviate inflammation and ameliorate disease symptoms. In a clinical Phase I/IIa nonrandomized trial (CATS1 study) led by Colombel, Tregs were isolated from 20 subjects, activated and expanded ex vivo, and subsequently reinfused into patients, resulting in significant symptom improvement within 8 weeks posttreatment for eight patients, with two patients approaching remission [167]. Research by Eshhal from the Weizmann Institute of Science validated the therapeutic efficacy of CAR–Tregs in colitis using a mouse model induced by 2,4,6‐trinitrobenzene sulfonic acid [168]. Furthermore, CAR–Tregs targeting different antigens, such as carcinoembryonic antigen, have shown effectiveness in suppressing colitis [169]. Kuchroo et al. from Harvard Medical School employed second‐generation CAR‐transduced Tregs for CD treatment, integrating a CAR with a CD28 costimulatory domain targeting IL‐23R. In murine models, these CAR–Tregs could inhibit T cell proliferation, localize to target organs, and reduce intestinal inflammation [170].

#### 4.7.2 CAR–Treg Therapy for MS

MS is an autoimmune demyelinating and neurodegenerative disease where genetic and environmental factors play critical roles in its onset. In MS, autoreactive T cells recognize antigenic epitopes of nerve cell myelin, leading to impaired nerve conduction function. Compared with healthy controls, patients with MS exhibit increased secretion of IFN‐γ and reduced IL‐10 by Tregs, indicating that the inhibitory activity of Tregs is disrupted in disease progression [171]. Loss of the transcription factor Foxp3 may lead Tregs to acquire proinflammatory characteristics, exacerbating disease progression or significantly increasing immune responses [172, 173]. Transfection of T cells to express FoxP3 can induce regulatory activity to address low peripheral blood‐derived Treg cell numbers and FoxP3 expression deficiency in endogenous Tregs [174]. Willekens et al. from the University of Antwerp genetically modified CD4+ T cells in an MS mouse model to coexpress a CAR targeting myelin oligodendrocyte glycoprotein (MOG) and FOXP3. The resulting MOG–CAR Tregs successfully entered the mouse brain, reducing levels of proinflammatory cytokine mRNA in brain tissue. Compared with the control group, mice treated with MOG–CAR Tregs showed disease improvement [175].

#### 4.7.3 CAR–Treg Therapy for T1D

Patients with T1D demonstrate reduced immune inhibitory function in Tregs, paving the way for using CAR–Tregs in T1D therapy [176]. GAD is a crucial autoantigen found in pancreatic islets, the CNS, and testes. Approximately 70% of T1D patients have anti‐GAD antibodies at diagnosis. Shahnawazmam et al. [177] designed CAR–Tregs targeting the GAD65 epitope on β cells. After infusion, these CAR–Tregs located in the pancreatic islets of a humanized T1D mouse model within 24 h. Compared with the control group, the CAR–Treg treatment group showed a significant increase in Tregs in the pancreas and spleen, along with lower blood glucose levels. Furthermore, allogeneic islet transplantation is a promising T1D treatment, yet host immune system‐mediated rejection limits its widespread application. Pierini et al. [178] engineered FTC CAR–Tregs to prolong the survival of allogeneic islet grafts in vivo, indicating the potential of CAR–Tregs in treating T1D.

Inspired by the recent success of HLA‐A*02‐restricted CAR–Tregs in establishing localized immune tolerance, the research team led by Pieper et al. [179] engineered β‐cell–targeted CARs specific for the antigen ENTPD3. Leveraging a novel phage display platform, they generated multiple CAR constructs recognizing the natively folded ENTPD3 protein, which is selectively expressed on pancreatic β cells. These CARs were transduced into natural Tregs to redirect their antigen specificity in an MHC‐unrestricted manner, thereby enabling precise, localized immune regulation within the islet microenvironment. In the spontaneous T1D model using NOD mice, ENTPD3‐specific CAR Tregs demonstrated preferential homing to pancreatic islets, local activation, robust expansion, and sustained persistence—collectively resulting in effective prevention of T1D onset. In contrast, control CAR Tregs showed no therapeutic benefit. Comprehensive phenotypic and functional characterization of human ENTPD3–CAR Tregs revealed a stable regulatory signature, potent Treg activation capacity, and strong immunosuppressive function. Critically, these ENTPD3–CAR Tregs were fully activated upon recognition of human pancreatic islets, confirming their physiological relevance and target specificity. ENTPD3‐specific CAR Tregs thus represent a promising and durable therapeutic strategy to achieve localized immune control in individuals at the prediabetic stage, those with newly diagnosed T1D, or patients undergoing β‐cell replacement therapies. This approach holds transformative potential to restore immune homeostasis within the islet niche while avoiding systemic immunosuppression—offering a path toward disease modification and long‐term remission in T1D.

#### 4.7.4 CAR–Treg Therapy for Vitiligo

Vitiligo is a progressive skin disorder characterized by depigmentation, with histological examination of affected skin revealing loss of melanocytes and a deficiency in and reduced function of Tregs [180, 181]. The overexpression of ganglioside D3 (GD3) membrane antigen in melanocytes makes it an ideal target for CAR–Tregs to target melanocytes. Mukhatayev et al. [182] found that mice treated with CAR–Tregs targeting GD3 exhibited higher levels of IL‐10, inhibition of melanocyte destruction, and delayed depigmentation. These findings support the use of CAR–Tregs in treating vitiligo to control depigmentation and promote immune tolerance.

#### 4.7.5 CAR–Treg Therapy for RA

Inducing immune tolerance in affected joint synovium through the development of specific CAR–Tregs is a promising strategy for RA treatment. Wright et al. [183] utilized ovalbumin (OVA)‐specific Tregs to suppress OVA‐induced arthritis. Both primary Tregs transduced with TCR and CD4+ T cells induced with TCR‐FoxP3 showed bystander suppression by reducing the proliferation of different antigen‐specific T cells. TCR‐Tregs and TCR‐FoxP3‐induced Tregs localized to damaged tissues, reducing the number of inflammatory Th17 cells and significantly decreasing arthritis‐related bone destruction. CCP (CV) is a specific antigen present only in the extracellular matrix of inflamed synovial tissue in RA patients. In conclusion, CAR‐T cell therapy demonstrates good efficacy and safety in AIDs. This therapy shows significant effectiveness in controlling disease activity, reducing organ damage, and ultimately achieving sustained drug‐free remission. Designing complex targets holds promise for further enhancing therapeutic outcomes.

#### 4.7.6 CAR–Treg Therapy for SLE

Doglio et al. [184] described a novel therapeutic approach employing Tregs engineered to overexpress the master transcription factor FoxP3 and to express a CD19‐targeted CAR (termed Fox19CAR–Tregs). In vitro, Fox19CAR–Tregs potently suppressed the proliferation and activation of B cells—key drivers of SLE pathogenesis. In a humanized mouse model of SLE, a single infusion of Fox19CAR–Tregs effectively restricted autoantibody production, delayed the onset of lymphopenia (a hallmark of SLE), and restored the composition of the human immune compartment within lymphoid organs, with no detectable toxicity. Although the persistence of the infused cells was limited, target organs typically affected in SLE appeared protected from damage. Collectively, these findings demonstrate that repeated administration of CAR–Tregs during early disease stages can effectively modulate pathogenic circulating B cells and reshape the human immune infiltrate in lymphoid tissues. Importantly, Fox19CAR–Tregs exhibited a favorable safety profile, showing no evidence of B‐cell analysis or secretion of proinflammatory cytokines. Thus, Fox19CAR–Tregs represent a potent and safe immunomodulatory strategy capable of interrupting the vicious cycle of autoimmunity and chronic tissue injury, thereby restoring immune homeostasis in SLE. Furthermore, to further enhance the completeness of our manuscript, we have added details of ongoing clinical trials of CAR‐T therapy for AIDs, including the NCT number, status, phase, and study objectives. For full details, please refer to Table S1.

## 5 Challenges Facing CAR‐T Cell Therapy in AIDs

This section systematically dissects the core challenges, underlying mechanisms, and potential breakthrough directions of CAR‐T cell therapy in AIDs, establishing a comprehensive framework for understanding its translational bottlenecks and optimization paths. The content is structured to progress from practical clinical challenges to mechanistic explorations, and finally to technology‐driven solutions: first, it focuses on clinical practicality issues including safety risks (e.g., CRS, infections, LICATS), economic feasibility of UCAR‐T, optimal treatment timing, lymphodepletion applicability, and efficacy sustainability (Sections 4.1–4.7); second, it delves into the biological mechanisms underlying therapeutic effects and limitations, such as B/T cell dynamics, immune reset pathways, and tissue penetration characteristics of CAR‐T cells in AIDs (Sections 4.8–4.10); finally, it proposes a multidimensional omics‐based strategy to address key scientific questions—covering therapeutic heterogeneity, relapse mechanisms, product optimization, and personalized combination therapy—providing a data‐driven and mechanism‐informed roadmap for advancing CAR‐T therapy in AIDs (Section 4.11). Collectively, this section integrates clinical practice, molecular mechanisms, and cutting‐edge technologies to comprehensively clarify the current predicament of CAR‐T therapy in AIDs and point out future research priorities, laying a foundation for subsequent discussions on optimization strategies.

### 5.1 Safety of CAR‐T Cell Therapy

Compared with patients with relapsed or refractory hematologic malignancies, the mortality rate in patients with AIDs is relatively lower, necessitating a more accurate risk–benefit evaluation. Although SAEs have not been observed in AID patients receiving CAR‐T cell therapy as reported to date, the short follow‐up duration highlights the need for close monitoring of long‐term safety characteristics. In the field of hematologic malignancies, adverse reactions include CRS, immune effector cell‐associated hematotoxicity, infections, and secondary tumors. Based on existing research on AIDs, Grade 1 CRS is the most common. Recent studies indicate that after CAR‐T cell recognition of target cells, the release of perforin and granzyme B activates the caspase3–GSDME pathway leading to pyroptosis, which further stimulates macrophages, activating the caspase1–GSDMD pathway to produce IL‐6 and IL‐1β, triggering CRS [185]. Management strategies for CRS include reducing CAR‐T cell dosages, using steroids or blocking IL‐6 receptor antibodies [185], and inhibiting macrophage inflammatory cytokines release by catecholamine blockers [186]. Infections are a primary cause of mortality post‐CAR‐T cell therapy. Studies show that nearly two‐thirds of patients among 60 individuals treated with CAR‐T cells developed fever after lymphodepletion and CAR‐T cell infusion, with 25% exhibiting microbiologically or radiographically confirmed infections mainly during early hospitalization stages [187]. Early identification of high‐risk patients and standardized anti‐infective measures are crucial. In terms of hematologic suppression, neutropenia induced by lymphodepletion chemotherapy is often self‐limiting and recovers rapidly, yet high‐grade CRS and associated inflammatory cytokine release may lead to intermittent recovery patterns or bone marrow failure phenotypes [188]. Patients with sustained neutropenia two months post‐CAR‐T cell infusion have a significantly increased risk of severe infections [188]. Prophylactic use of granulocyte‐macrophage colony‐stimulating factor, autologous stem cell second consolidative transplantation, and the use of anti‐inflammatory or anticytokine medications to improve high‐inflammatory states may be effective treatment approaches. Regarding long‐term adverse reactions, Hamilton et al. [189] observed 25 cases of secondary tumors, including one fatal T‐cell lymphoma, in a study involving 724 patients receiving CAR‐T cell therapy. Although the risk of secondary tumors is low, close monitoring during long‐term follow‐up is essential.

However, based on experiences from our research team and global studies on CAR‐T cell therapy for AIDs, AID patients are typically younger, healthier, and have lower mortality rates compared with cancer patients, exhibiting lower tolerance to severe side effects. CRS appears less common in AID patients, possibly due to lower B‐cell burdens. Few reported cases of ICANS post‐CAR‐T cell therapy in SLE patients have limited clinical impact. The association between CAR structure, patient disease status, and adverse reactions remains unclear, necessitating more data to assess risk–benefit ratios. Additionally, in March 2024, the US FDA reported cases of T‐cell malignancies post‐CAR‐T cell therapy [190]. The US FDA mandated black box warnings for CAR‐T therapy. Although it remains unclear if AID patients undergoing treatment will develop T‐cell malignancies, this is another factor to consider in evaluating the risk–benefit ratio of this treatment. Long‐term monitoring is essential. Other factors associated with secondary malignancies include insertional mutations from integrating vectors and inherent cancer predisposition in patients. To mitigate the oncogenic risk of insertional mutations, the use of safer vectors or gene editing systems may be beneficial. Furthermore, evaluating the uncertain potential of CHIP may help identify patients at higher risk of secondary malignancies, requiring more intensive follow‐up screening. These reactions could lead to severe metabolic and endocrine imbalances affecting the reproductive system. Consideration of the potential reproductive toxicity of CAR‐T cell therapy is warranted.

### 5.2 Economic Feasibility of UCAR‐T Cells in the Future of CAR‐T Cell Therapy for AIDs

The advent of UCAR‐T cells has brought new hope for the future of CAR‐T cell therapy in AIDs, reducing the difficulty in cell production. Patients with AIDs typically receive glucocorticoids and various autoimmune suppressants in the early stages of treatment. These drugs may affect the quality and quantity of T cells, making it challenging to extract a sufficient number of functional T cells from patients and subsequent cell amplification. Collecting more lymphocytes may increase the clone number of autoreactive T cells, posing a risk of exacerbating the condition postinfusion. Additionally, the production process of CAR‐T cells is complex and expensive, usually requiring 2–4 weeks for the entire manufacturing cycle. Current efforts aim to reduce treatment complexity and costs by gradually reducing or eliminating lymphodepletion therapy and shifting treatment to outpatient settings. The newly developed mRNA CAR‐T cells can rapidly degrade upon infusion into the body, leading to loss of CAR expression, thereby reducing the long‐term immunosuppressive requirements and lowering dependence on lymphodepletion therapy. It significantly decreases treatment complexity and potential toxic side effects. A study involving 14 patients with severe generalized MG who were refractory to standard immunosuppressive therapy and had not undergone lymphodepletion treatment received Descartes‐08 treatment (mRNA CAR‐T cell therapy) in outpatient settings with varying doses and dosing regimens. The study observed a significant decrease in the severity of MG in patients over a 12‐month follow‐up period [94]. UCAR‐T cells, also known as allogeneic CAR‐T cells, are T cells collected from healthy donors, genetically engineered ex vivo, and massively expanded to produce off‐the‐shelf cellular therapy. Due to their origin from healthy donors, unaffected by patient T cell quantity and quality, they have a high success rate in preparation. with a single batch capable of meeting the demands of hundreds of individuals, offering significant time and cost advantages and benefiting more patients [191].

Therefore, in recent years, UCAR‐T cell therapy has sparked a research frenzy in the field of cell therapy. However, challenges persist in the development of allogeneic CAR‐T cells due to potential graft rejection reactions. Overcoming these challenges will be crucial for the future. A recent study [112] was the first internationally to report the use of allogeneic UCAR‐T cells in treating rheumatic AIDs. Their groundbreaking clinical research results demonstrate that allogeneic anti‐CD19 CAR‐T cells can effectively treat refractory and relapsing rheumatic AID patients who have failed existing treatment regimens with good tolerability. This highlights the enormous therapeutic potential and market value of UCAR‐T therapy. At the technical level, the research team utilized CRISPR–Cas9 gene editing technology to ensure cell survival and amplification in vivo while reducing off‐target effects, ensuring treatment safety and efficacy. The study results showed no occurrences of CRS or other SAEs during follow‐up, with significant clinical responses. This provides a new approach for treating refractory rheumatic autoimmune conditions, holding significant scientific importance. From a clinical application perspective, based on the high safety and feasibility of allo‐gene CAR‐T cells in treating refractory rheumatic AIDs, this study pioneers the application of allogeneic CAR‐T cells in this field, introducing a new therapeutic approach for rheumatic AID treatment with significant clinical potential. It lays the groundwork for future widespread application in a larger patient population, holding substantial clinical significance and practical value.

### 5.3 Timing of Efficacy in CAR‐T Cell Therapy

Currently, CAR‐T cell therapy is primarily used in the early stages for patients with refractory and high‐risk conditions as salvage therapy. However, these patients often have existing organ damage, and preliminary data indicate that CAR‐T cell therapy targeting B cells can sustainably eliminate inflammation but may not reverse tissue damage, limiting the extent of disease improvement posttreatment. Therefore, in considering patients for clinical trials or actual clinical treatment processes, researchers and clinicians may need to focus on those with refractory diseases without organ damage, as these patients may ultimately benefit the most, especially given the current high cost of CAR‐T cell therapy [192]. Furthermore, most clinical trials evaluating CAR‐T cell therapy for AIDs are inspired by the success in treating B‐cell malignancies, utilizing CAR‐T cells targeting CD19 alone or in combination with BCMA to eliminate B cells. In future developments for AIDs, there is a need to refine target selection: whether single‐targeting CD19 is more effective or combination targeting of CD19/BCMA; whether targeting CD19 is more suitable or targeting CD20, CD22, and CD38 is preferable. Some studies indicate that plasma cells and LLPCs could be sources of autoantibodies. Throughout the adaptive immune response, antigen‐specific B cells differentiate into memory B cells and antibody‐secreting cells (including plasma cells and LLPCs). The specific roles of these cells in AIDs may vary depending on the type of disease. The primary sources of autoantibodies in AIDs are complex, likely involving plasma cells and LLPCs, with potential differences between diseases or individuals [193].

### 5.4 The Applicability of Lymphodepletion in CAR‐T Cell Therapy for AIDs

Lymphodepletion plays a crucial role in CAR‐T cell therapy, primarily to eliminate lymphocytes within patients, creating an immunological environment conducive to the expansion of CAR‐T cells. In hematologic malignancies, lymphodepletion can eradicate endogenous lymphocytes, modulate the tumor microenvironment to support the expansion and long‐term survival of CAR‐T cells, and reduce tumor burden. However, lymphodepletion also brings adverse effects: the chemotherapeutic agents used in lymphodepletion indiscriminately kill or inhibit hematopoietic stem cells, making patients vulnerable to infections. For autoimmune patients where tumor cell eradication is unnecessary, and there is no tumor cell infiltration in the homing tissues of CAR‐T cells in bone marrow and lymph nodes, lymphodepletion may act by eliminating endogenous lymphocytes. In the treatment experience of lupus patients, the use of CTX and fludarabine for standard immunodepletion can complicate result interpretation. These drugs have demonstrated efficacy as standalone treatments for SLE and LN but come with significant side effects, including increased rates of bacterial and viral infections. Current research [194] suggests that lymphodepletion remains important for CAR‐T therapy in autoimmune patients, but the optimal lymphodepletion drug regimen and dosage are yet to be determined, necessitating exploration through preclinical and clinical experiments. This research is of low priority and can be deferred until the efficacy and safety of CAR‐T therapy in various AIDs are established using existing lymphodepletion drugs. Moreover, from a pharmacoeconomic perspective, considering the high costs associated with CAR‐T cell therapy is crucial. If the therapy can provide sustained medication‐free remission and minimal toxicity for all patients, its necessity becomes clear. However, if it only benefits some patients without achieving remission and comes with concerning side effects, it is essential to match specific CAR‐T regimens with the most suitable patients to improve pharmacoeconomic balance.

### 5.5 The Sustainability of CAR‐T Cell Therapy Efficacy

The duration of medication‐free sustained remission following CAR‐T cell therapy for AIDs remains uncertain. In SLE patients, B cells often experience rapid reconstitution several months posttreatment, indicating a low likelihood of long‐term persistence of CAR‐T cells [65, 195]. Moreover, new B cell proliferation and maturation can occur after new infections or vaccinations, potentially stimulating the reactivation of B cells producing autoantibodies, leading to disease relapse. Reasons for the rapid reconstitution of B cells include minimal cytotoxic drug use in autoimmune patients, making it easier for B cells to occupy ecological niches in bone marrow or lymph nodes; the rapid depletion of target antigens triggers early exhaustion of CAR‐T cells. While the sustained presence of CAR‐T cells may aid in seroconversion, prolonged depletion of B cells can also impact immune function. Muller and colleagues [125] recently reported a patient with refractory Jo‐1‐positive ASS who achieved an initial robust remission following CD19‐directed CAR‐T cell therapy. However, disease relapse subsequently occurred, and a second infusion of the same CD19–CAR‐T cells failed to reinduce control. Notably, the patient subsequently responded to a distinct therapeutic modality—BCMA‐targeted CAR‐T cell therapy—achieving a second drug‐free remission. This case underscores a critical challenge in the application of CAR‐T cell therapy to AIDs: the limited efficacy of repeated dosing with identical CAR constructs, likely due to immune reconstitution, antigen escape, or loss of CAR‐T cell persistence. In a parallel report, Pecher et al. [196] described a 32‐year‐old woman with concomitant severe RA and relapsing–remitting MS who experienced relapse after initial response to anti‐CD19 CAR‐T cell therapy but was successfully rescued with autologous HSCT. The patient was diagnosed with MS at the age of 20 years and, due to high disease activity, received IFN β‐1a followed by fingolimod. In spring 2016, she achieved sustained MS remission after a 5‐day course of alemtuzumab (12 mg/day). However, in March 2017, she developed symmetric, erosive polyarthritis and was diagnosed with seronegative RA. The arthritis followed a highly aggressive course and proved refractory to multiple therapeutic modalities, including methotrexate, RTX, abatacept, anakinra, several JAK inhibitors, tocilizumab, TNF inhibitors, six cycles of CTX (750 mg/m^2^), and secukinumab—none of which provided durable remission. Faced with a lack of effective options, the patient received investigational CD19‐targeted CAR‐T cell therapy in May 2023 under an academic protocol. She was discharged on Day 11 postinfusion and successfully tapered off hydrocortisone by Day 150. Early clinical improvement was evident, with rapid declines in CRP and DAS in 28 joints (DAS28), achieving clinical remission by Day 28. However, severe synovitis recurred 8 months later, coinciding with B‐cell reconstitution (>200 CD19^+^ B cells/µL) several weeks prior. As a salvage strategy, autologous HSCT was performed in April 2024. Stem cells were mobilized using CTX (CYC) plus granulocyte colony‐stimulating factor and CD34^+^‐selected for reinfusion. The conditioning regimen consisted of high‐dose CYC (4 × 50 mg/kg) combined with rabbit antithymocyte globulin (Grafalon, total dose 23.5 mg/kg). Notably, no posttransplant immunosuppression was administered. Following HSCT, DAS28 and CRP levels continued to decline, and the patient reported marked subjective improvement in overall health. A SAE—CNS varicella‐zoster virus reactivation—occurred despite prophylactic antiviral therapy. Immunophenotyping revealed preserved B‐cell reconstitution but profound and sustained T‐cell depletion post‐HSCT. To our knowledge, this represents the first reported case of successful sequential intervention with HSCT following failed sustained remission after CD19 CAR‐T cell therapy in a B‐cell‐driven AID. Of particular interest, the patient's seronegative polyarthritis emerged after alemtuzumab treatment and exhibited atypical features. Although it met the 2010 ACR/EULAR classification criteria for RA, we hypothesize that this highly treatment‐refractory phenotype may represent alemtuzumab‐induced secondary autoimmune arthritis. While alemtuzumab is well recognized for triggering secondary AIDs—most commonly thyroid autoimmunity or ITP—drug‐induced arthritis remains comparatively rare. Collectively, these cases highlight both the transformative potential and current limitations of CAR‐T cell therapy in AIDs. They reinforce that anti‐CD19 CAR‐T cells remain a highly effective strategy for classical (seropositive) RA, while also underscoring the need for alternative or sequential approaches—such as HSCT—in patients with atypical, treatment‐refractory phenotypes or those who relapse after initial CAR‐T cell response. Therefore, there is a need to balance the duration of CAR‐T cell persistence with the risk of B cell depletion to optimize efficacy and safety.

### 5.6 Predictive Biomarkers for Efficacy and Safety of CAR‐T Cell Therapy

In addition to traditional efficacy evaluation indicators (such as anti‐dsDNA antibody levels in SLE), specific biomarkers for monitoring the efficacy of CAR‐T cell therapy exist. Prior to infusion, a high proportion of initial T cells and central memory T cells expressing markers may indicate stronger antitumor effects [197], which could be applied in the field of AIDs. Postinfusion, dynamic changes in peripheral blood B cells can assess the clearance time of B cells; the immunophenotype of reconstituted B cells can also indicate the depth of clearance and the extent of immune reconstitution. Furthermore, early memory differentiation‐related transcription factors (such as T cell factor 7, lymphoid enhancer‐binding factor), T cell activation markers (such as CD25, CD69, CD137), and exhaustion markers (such as CD57, PD‐1, Tim‐3) can be used to evaluate patient sensitivity to treatment and the killing capacity of T cells [198]. In terms of safety assessment, identifying biomarkers associated with CRS and ICANS is crucial. Upon binding to target antigens in the body, CAR‐T cells secrete cytokines (such as IFN‐γ, IL‐2, IL‐6) and activate endogenous immune cells, triggering inflammatory responses. Therefore, monitoring the effector cytokines secreted by CAR‐T cells and proinflammatory factors secreted by immune cells (such as monocytes, macrophages, etc.) can evaluate the level of inflammatory response. Studies have shown that the levels of IL‐8, IL‐10, and monocyte chemoattractant protein 1 in CSF can predict the occurrence of ICANS [199], while an infection‐related model composed of IL‐8, IFN‐γ, and IL‐1β can effectively predict postinfusion infection risk [200].

### 5.7 Potential Future Adverse Effects of CAR‐T Cell Therapy in AIDs

Current research on the adverse effects of CAR‐T cell therapy has predominantly focused on observations in cancer patients; the specific side effects in patients with AIDs remain poorly understood. In anticipation of emerging challenges related to novel AEs, we must closely monitor newly arising side effects during CAR‐T therapy in AID contexts. For instance, a recent study by Georg Schett's team [201] revealed that 77% of AID patients treated with CD19‐targeted CAR‐T cells developed a mysterious “organ‐specific inflammation” that superficially resembles disease relapse but is fundamentally distinct. The researchers named this phenomenon “local immune effector cell‐associated toxicity syndrome” (LICATS). Is this a warning sign—or an inevitable step toward cure? This study enrolled 39 patients with AIDs who received CD19‐targeted CAR‐T cell therapy between March 1, 2021 and October 31, 2024, at two German medical centers. All patients were followed for at least 30 days postinfusion to evaluate organ‐specific local reactions. Observed reactions were documented according to anatomical site, onset time, duration, and graded by severity. Among the 39 patients, 20 had SLE, 13 had SSc, and six had IIM; 25 were female and 14 male, with ages ranging from 22 to 44 years (mean: 36 years). The research team documented a total of 54 AEs, collectively designated as LICATS. Thirty patients (77%) experienced 54 episodes of LICATS, with a median onset of 10 days postinfusion and a median duration of 11 days. Among the 30 affected patients, 15 experienced only one episode, while the remaining 15 had multiple episodes. Notably, LICATS occurred exclusively during the period of B‐cell aplasia and was confined to organs previously involved by the underlying AID. The most commonly affected organs were skin (erythema, oral ulcers, etc.; 19 out of 54 cases, 35%), kidneys (transient proteinuria/creatinine elevation; 12 cases, 22%), and the musculoskeletal system (transient CK elevation; 10 cases, 19%). The majority of LICATS events were mild: Grade 1 (35 cases, 65%) or Grade 2 (16 cases, 30%). Only three cases reached Grade 3 severity, requiring hospitalization; none required intensive care. Of all 54 LICATS events, 35 (65%) resolved spontaneously without intervention, 16 (30%) resolved with short‐course, low‐dose corticosteroid therapy, and only three (5%) required hospitalization. Ultimately, all LICATS episodes fully resolved without long‐term damage or sequelae. Inspired by these findings, Ye et al. from the Department of Rheumatology at Renji Hospital [202] conducted a systematic analysis of LICATS cases emerging in two ongoing clinical trials of CAR‐T therapy for rSLE at their center (one single‐center Phase I trial and one multicenter Phase I‐II trial). They observed a similar phenomenon. In these trials, which employed autologous dual‐target CD19–BCMA CAR‐T cells for rSLE, 19 patients completed at least 30 days of follow‐up. Four patients (21%) experienced a total of seven localized inflammatory episodes meeting LICATS criteria. The median onset of LICATS was 14 days postinfusion, with a median duration of 3 days. Most reactions were mild, primarily affecting joints and kidneys, followed by rash, pleuritis, and diarrhea. All AEs were mild (Grade 1–2) and resolved without sequelae. Notably, arthritis and diarrhea occurred in two patients with no prior history of these manifestations, and their timing clearly differed from the typical CRS course. The authors suggest these clinical features indicate that LICATS may exhibit broader clinical heterogeneity than currently recognized. Therefore, there is an urgent need to establish a unified, cross‐platform diagnostic criterion for LICATS and to systematically integrate clinical toxicity data to further elucidate its mechanisms, risk factors, and clinical spectrum. In summary, LICATS exhibits distinct characteristics that differentiate it from disease flares: (1) self‐limiting nature: 65% of cases resolved without intervention; 30% responded to short‐term corticosteroids; only 5% required hospitalization. (2) Temporal association: occurs exclusively during B‐cell aplasia and correlates with CAR‐T cell activity. (3) Absence of immunological biomarkers: no complement activation, autoantibody re‐emergence, or B‐cell infiltration in affected tissues. (4) Organ‐specific restriction: confined to previously involved organs, sparing healthy tissues. (5) Potential mechanisms: may involve macrophage overload (due to clearance of apoptotic B cells) and transient inflammatory responses during immune complex clearance. Recognizing LICATS is critical to avoid misdiagnosis as disease relapse and to prevent unnecessary immunosuppressive interventions.

### 5.8 Mechanisms of Immune Reset by CAR‐T Cells in AIDs—B Cell Depletion and Plasma Cell Dynamics

The primary mechanism underlying the efficacy of CAR‐T cell therapy in AIDs involves targeted elimination of pathogenic B‐cell lineages. However, therapeutic outcomes appear to transcend simple B‐cell depletion, instead promoting a profound phenomenon of immune reconstitution. Following CAR‐T infusion, patients experience rapid depletion of CD19+ B cells within days, with B‐cell reconstitution typically commencing between 2 and 6 months posttreatment. Critically, the reconstituted B‐cell compartment exhibits a predominantly naïve phenotype, with marked reduction of CD27+ memory B cells and near‐complete elimination of pathogenic subsets implicated in SLE pathogenesis—such as CD27+CD38+ plasmablasts and CD11c+ memory B cells [203]. This immune reset is further corroborated by serological changes, including rapid decline of autoantibodies (anti‐dsDNA, anti‐Smith, antinucleosome) and normalization of complement levels. The post‐CAR‐T B‐cell repertoire demonstrates reduced autoreactivity while preserving responsiveness to foreign antigens, indicating a recalibration of immune homeostasis rather than mere immunosuppression. This phenomenon may explain the sustained clinical benefit observed even after B‐cell repopulation and waning CAR‐T persistence. Importantly, significant distinctions exist between different targeting strategies. CD19‐directed CAR‐T cells efficiently deplete B‐lineage cells across all differentiation stages but exhibit limited efficacy against CD19‐negative LLPCs. This limitation may account for incomplete autoantibody reduction in certain patients and potential disease relapse driven by residual plasma cells. In contrast, BCMA‐targeted approaches demonstrate superior clearance of plasma cells, including long‐lived populations residing within the bone marrow niche. However, BCMA targeting carries a higher risk of profound hypogammaglobulinemia due to elimination of antibody‐producing cells. Innovative dual‐targeting strategies (CD19/BCMA CAR‐T) aim to balance comprehensive depletion with acceptable safety profiles, although clinical experience remains limited. Notably, despite minimal BCMA expression on circulating B cells, BCMA‐directed CAR‐T cells can still eliminate these cells—suggesting alternative mechanisms, potentially involving soluble BCMA or bystander effects. This observation underscores the biological complexity of CAR‐T cells, extending beyond direct target engagement.

### 5.9 T Cell Dynamics and Persistence of CAR‐T Therapy in AIDs

CAR‐T cell persistence patterns differ markedly between AIDs and malignancies. In contrast to the prolonged persistence often observed in lymphoma, CAR‐T cells in autoimmune patients typically exhibit shorter durability. This reduced persistence may stem from several factors: (1) lower antigen burden, resulting in diminished sustained CAR‐T activation; (2) differences in host immunogenicity toward the CAR construct; (3) the unique inflammatory microenvironment of AIDs; and (4) rapid resolution of inflammation, which attenuates persistent signaling cues. Despite shorter persistence, clinical benefits commonly extend well beyond the detectable lifespan of CAR‐T cells, suggesting that transient but potent B‐cell depletion may suffice to disrupt pathological immune circuits and permit resetting of tolerance mechanisms. Emerging data [203] reveal that CAR‐T therapy profoundly reshapes the endogenous T‐cell landscape. Single‐cell analyses demonstrate exhaustion of certain T‐cell subsets and emergence of novel transitional populations. In MM models, specific transitional CD8+ T‐cell populations have been associated with inferior therapeutic outcomes, implying that analogous mechanisms may operate within autoimmune contexts. The dynamic interplay between CAR‐T cells and the endogenous immune milieu represents a critical frontier for future research.

### 5.10 Tissue Penetration and Organ‐Specific Effects of CAR‐T Cells in AIDs

In AIDs, pathogenic immune cells exhibit markedly distinct distributions between the circulation and tissue compartments. Antibody‐producing cells often reside within protected microenvironments—such as lymph nodes, bone marrow, and inflamed tissues. Traditionally, the therapeutic efficacy of CAR‐T cell therapy in AIDs has been attributed primarily to the systemic depletion of pathogenic B cells or plasma cells in the circulation. However, accumulating preclinical and early clinical evidence indicates that the therapeutic impact of CAR‐T cells extends far beyond broad clearance in peripheral blood or lymphoid organs. A critical determinant of treatment depth, durability, and even the potential for “functional cure” lies in whether CAR‐T cells can effectively infiltrate disease‐targeted organs—such as the kidneys, skin, joints, CNS, and pancreatic islets—and exert direct or indirect immunomodulatory effects within local tissue microenvironments. Successful CAR‐T therapy must overcome delivery barriers to access these tissue “sanctuaries.” Evidence suggests that CAR‐T cells can indeed penetrate target tissues, as demonstrated by clinical improvements in organ‐specific manifestations such as LN, cutaneous fibrosis, and myositis. The organ‐restricted emergence of local inflammatory reactions in previously affected tissues further supports the tissue‐penetrating capacity of CAR‐T cells. Advanced monitoring technologies, such as multiplex digital PCR, enable precise quantification of CAR‐T cells as a proportion of total T cells, thereby providing deep insights into their expansion kinetics and tissue distribution patterns. These tools are invaluable for establishing correlations between CAR‐T cell PK and clinical outcomes. Tissue penetration refers to the efficiency—and limitations—with which CAR‐T cells traverse “blood‐tissue barriers.” Not all CAR‐T cells reach all inflammatory sites with equal efficacy. This capacity is governed by multiple factors: (1) compatibility between chemokines and their cognate receptors; (2) the influence of CAR construct design on migratory potential; and (3) physical barriers and challenges posed by immune‐privileged microenvironments. Within target organs, CAR‐T cells exert effects beyond direct killing of antigen‐expressing cells. More crucially, they possess the ability to “reprogram” the local immune ecosystem, including: (1) eliminating pathogenic resident immune cells within tertiary lymphoid structures in organs such as the kidney, skin, and salivary glands; (2) modulating local innate immune and stromal cells; and (3) promoting tissue repair and establishment of a tolerogenic microenvironment.

However, tissue penetration is a double‐edged sword. While efficient homing to target organs enhances therapeutic efficacy, it may concurrently increase the risk of “off‐target” toxicity to healthy tissues, particularly when the target antigen is expressed at low levels on nonimmune cells. For example, although CD19 is predominantly expressed on B cells, studies have identified CD19+ B cell populations in the meninges, potentially involved in central immune surveillance. Could massive infiltration of CAR‐T cells into the CNS and subsequent elimination of these cells disrupt meningeal immune homeostasis or induce cognitive alterations? Although current clinical data report no significant neuropsychiatric AEs, long‐term follow‐up remains essential. Similarly, BCMA‐targeted CAR‐T cells entering the brain parenchyma in large numbers could theoretically pose a risk of demyelination due to low‐level BCMA expression on neurons or oligodendrocytes—though no such events have been reported to date, vigilance is warranted.

To maximize organ‐specific efficacy while minimizing toxicity, next‐generation CAR‐T designs must integrate “tissue navigation” and “microenvironment sensing” capabilities, giving rise to “organ‐intelligent” CAR‐T cells. Strategies include engineering CAR constructs that are fully activated only in microenvironments rich in inflammatory signals (e.g., TNF‐α, IFN‐γ, hypoxia)—for instance, placing CAR expression under the control of NF‐κB‐ or HIF‐1α‐responsive promoters, thereby “arming” CAR‐T cells exclusively within inflammatory lesions. For organ‐restricted diseases (e.g., primary biliary cholangitis, localized scleroderma), localized delivery routes (e.g., hepatic artery infusion, intralesional injection) may be explored to enhance local concentration while reducing systemic exposure. In vivo CAR‐T technologies, such as organ‐targeted LNP delivery, hold promise for achieving tissue‐specific transfection in organs like the liver and spleen. Furthermore, personalized homing strategies based on multiomics profiling—mapping disease‐specific chemokine landscapes via single‐cell RNA sequencing and spatial transcriptomics of patient biopsy tissues—could enable the customization of CAR‐T products expressing matched chemokine receptors, thereby achieving “precision navigation.”

Assessment of organ‐specific CAR‐T effects should not rely solely on peripheral blood biomarkers but must integrate multimodal approaches: imaging‐guided functional evaluation, liquid biopsy, and analysis of tissue‐specific biomarkers. Therapeutic biopsies performed at critical timepoints (e.g., 3–6 months postinfusion), though invasive, offer irreplaceable scientific value by directly quantifying CAR‐T cell infiltration density, target cell clearance, extent of immune microenvironment remodeling (e.g., Treg/Teff ratios, macrophage polarization states), and signs of tissue repair. Such data are essential for mechanistic understanding and guiding subsequent interventions and must be implemented within rigorous ethical frameworks.

The future of CAR‐T therapy in AIDs will no longer be satisfied with merely “depleting circulating B cells,” but will strive to “precisely locate and reset the immune microenvironment of diseased organs.” By engineering CAR‐T cells with capabilities for “tissue navigation,” “microenvironment sensing,” and “local immunomodulation,” this goal may be realized. The advent of the “organ‐intelligent CAR‐T” era will mark a paradigm shift in AID therapy—from “systemic immunosuppression” to “localized precision repair and cure.”

### 5.11 Advancing Key Scientific Questions in CAR‐T Cell Therapy for AIDs Through Multidimensional Omics: From Data‐Driven Insights to Mechanistic Understanding and Precision Intervention

The rapid evolution of CAR‐T cell therapy in AIDs (AIDs) is transitioning from empirical clinical exploration toward a new era of mechanism‐driven, precision immunomodulation. However, core scientific questions—including therapeutic heterogeneity, mechanisms of relapse, organ‐specific effects, trajectories of long‐term immune reconstitution, and strategies for personalized therapy—still lack systematic, molecular‐level resolution. Traditional “single‐omics + clinical phenotype” paradigms are insufficient to capture the high complexity and dynamism of AIDs. The integrated application of multidimensional omics technologies—encompassing single‐cell transcriptomics, epigenomics, TCR/BCR immune repertoire profiling, spatial transcriptomics, proteomics, metabolomics, and microbiome analysis—is emerging as the central engine for decoding mechanisms of CAR‐T response, predicting efficacy and toxicity, optimizing cell product design, and enabling individualized therapeutic pathways [204]. This section systematically outlines how multidimensional omics data can drive breakthroughs across six key scientific domains.

#### 5.11.1 Deciphering Therapeutic Heterogeneity: Constructing a “CAR‐T Response Atlas” and Predictive Models

Clinical observations reveal significant inter‐patient variability in response magnitude, depth of remission, and durability—even among patients with identical diseases (e.g., SLE) and identical targets (e.g., CD19). This heterogeneity stems from multilayered factors including host immune background, pathogenic clone characteristics, and microenvironmental states. (1) Single‐cell multiomics reveals immune landscape disparities between responders and nonresponders: integrated scRNA‐seq, scATAC‐seq, and CITE‐seq (surface protein) profiling of pretreatment peripheral blood and tissue biopsies can identify predictive cellular subsets—such as CXCR5‐high B cells, low Treg/Teff ratios, or specific macrophage polarization states—and their regulatory networks. For instance, lupus patients exhibiting high proportions of “activated memory B cells (CD27+CD21−)” or “plasmablasts (CD38+CD138+)” at baseline are more likely to achieve deep remission. (2) Dynamic tracking of pathogenic clones via BCR/TCR repertoire sequencing: high‐throughput immune repertoire sequencing enables longitudinal tracking of autoreactive BCR/TCR clones before and after CAR‐T therapy. Responders typically show complete eradication of pathogenic clones (e.g., anti‐dsDNA+ BCR clones) and restoration of repertoire diversity; relapsers often exhibit clonal escape (e.g., BCR mutations leading to antigen loss) or expansion of novel pathogenic clones. Machine learning can integrate these dynamics to construct a “Clonal Clearance Index” as a predictive biomarker of efficacy. (3) Integrative clinical‐omics algorithms for response prediction: AI models (e.g., graph neural networks, random forests) can synthesize baseline clinical metrics (SLEDAI, complement levels), serum proteomics (Olink platform), metabolomics (LC–MS), and immune repertoire data to generate a “CAR‐T Response Probability Score,” enabling pretreatment patient stratification and enrichment in clinical trial design.

#### 5.11.2 Elucidating Relapse Mechanisms: Systematic Decoding From “Antigen Escape” to “Immune Ecosystem Imbalance”

Relapse remains a central challenge in CAR‐T therapy for AIDs. While conventional views focus on “target antigen loss” (e.g., CD19‐negative B‐cell escape), multiomics reveals far more complex underlying mechanisms: (1) spatial multiomics pinpoints the anatomical “source” of relapse: spatial transcriptomics (e.g., Visium, MERFISH) combined with multiplex immunofluorescence can precisely localize residual or emergent pathogenic cell populations and their niche microenvironments within relapsed tissues. For example, in recurrent LN, CD19‐negative but BCMA‐positive plasma cell nests surrounded by PD‐L1+ macrophages and IL‐6+ fibroblasts have been identified in peritubular regions, forming an immune‐privileged niche. (2) Epigenetic reprogramming drives therapeutic resistance: scATAC‐seq and ChIP‐seq analyses reveal that relapse‐associated B or T cells frequently exhibit specific chromatin accessibility states (e.g., enhancer regions enriched for NF‐κB or STAT3 binding sites), conferring intrinsic resistance to CAR‐T‐mediated killing. Targeting these epigenetic regulators (e.g., with BET inhibitors) represents a rational combinatorial strategy. (3) Metabolic–immune axis imbalance promotes relapse: metabolomic profiling (e.g., Seahorse + mass spectrometry imaging) demonstrates accumulation of immunosuppressive metabolites—such as lactate and kynurenine—in tissues of relapsing patients, which impair CAR‐T cell function. Concurrently, autoreactive T cells adopt a “glycolysis–glutaminolysis dual‐dependency” metabolic phenotype, presenting a potential target for metabolic intervention.

#### 5.11.3 Optimizing CAR‐T Cell Product Design: From Empirical Screening to Data‐Driven Engineering

Current CAR construct designs (e.g., scFv selection, costimulatory domain combinations) are largely based on oncology experience or in vitro functional assays, lacking rational optimization for the unique AID microenvironment. Multiomics can provide a “design blueprint”: (1) homing receptor engineering guided by tissue chemokine maps: spatial transcriptomics can generate chemokine expression heatmaps of target organs (e.g., kidney, skin, CNS), informing engineering of CAR‐T cells to express matched homing receptors (e.g., CCR2/CXCR3 for kidney targeting; CXCR4 for CNS targeting). Animal models confirm that such “navigation‐enhanced CAR‐T” cells exhibit twofold to threefold improved efficacy. (2) Smart design of microenvironment‐responsive CARs: integration of scRNA‐seq and ATAC‐seq data can identify transcription factors specifically activated in inflamed tissues (e.g., NF‐κB, HIF‐1α, STAT1). Placing their response elements upstream of the CAR promoter enables construction of “lesion‐activated CARs” that express CAR only within inflammatory sites, minimizing off‐target toxicity. (3) Development of metabolically adapted CAR‐T cells: metabolomics reveals that AID lesions commonly exhibit “low glucose, high lactate, hypoxic” conditions. Accordingly, engineering CAR‐T cells with enhanced metabolic fitness—such as overexpression of MCT1 lactate transporters or LDHA knockout to reduce acidosis sensitivity—can significantly improve survival and function within hostile microenvironments.

#### 5.11.4 Unveiling Mechanisms of Organ‐Specific Effects: Immune Ecosystem Remodeling at Spatial Resolution

Organ‐specific efficacy of CAR‐T therapy is thought to depend on local infiltration and microenvironmental reprogramming. Multiomics, particularly spatial multiomics, offers revolutionary tools to dissect this process: (1) spatiotemporal decoding of “immune reset” via spatial transcriptomics + proteomics: in serial kidney biopsies from LN patients pre‐ and post‐CAR‐T, spatial multiomics can reconstruct the spatiotemporal cascade: CAR‐T infiltration trajectory → sequential clearance of pathogenic B cells/plasma cells → macrophage polarization (M1 → M2) → fibroblast deactivation → Treg recruitment → expression of tissue repair factors (e.g., VEGF, TGF‐β), thereby generating an “immune reset roadmap.” (2) Identification of organ‐specific toxicity signals: in brain tissue, spatial proteomics (e.g., CODEX) can detect neuronal damage markers (e.g., elevated S100β, GFAP) or signs of demyelination (MBP loss) within CAR‐T‐infiltrated zones—even in the absence of clinical symptoms—providing early warning of potential NT.

#### 5.11.5 Guiding Personalized Combination Strategies: “CAR‐T + X” Precision Combinations Based on Multiomics Subtyping

Given the profound heterogeneity of AIDs, monotherapy with CAR‐T cannot address all pathogenic axes. Multiomics enables molecular subtyping to guide rational combinations: (1) subtype 1: B‐cell/plasma cell dominant → CAR‐T (CD19/BCMA) + BAFF inhibitor (to consolidate B‐cell tolerance reconstitution); (2) subtype 2: T‐cell/cytokine storm dominant → CAR‐T + JAK inhibitor or anti‐IL‐6R (to control inflammation); (3) subtype 3: fibrosis/stromal activation dominant → CAR‐T + fibroblast activation protein (FAP)‐CAR or anti‐TGF‐β (to target fibroblasts); (4) subtype 4: gut microbiome dysbiosis dominant → CAR‐T + customized FMT or probiotics (to restore gut–immune axis). In sum, pretreatment multiomics subtyping allows matching each patient with an optimal “CAR‐T + X” combination, enabling true precision medicine.

#### 5.11.6 Building a Global, Shared “CAR‐T in AIDs Multiomics Knowledgebase”

**FIGURE 5:**
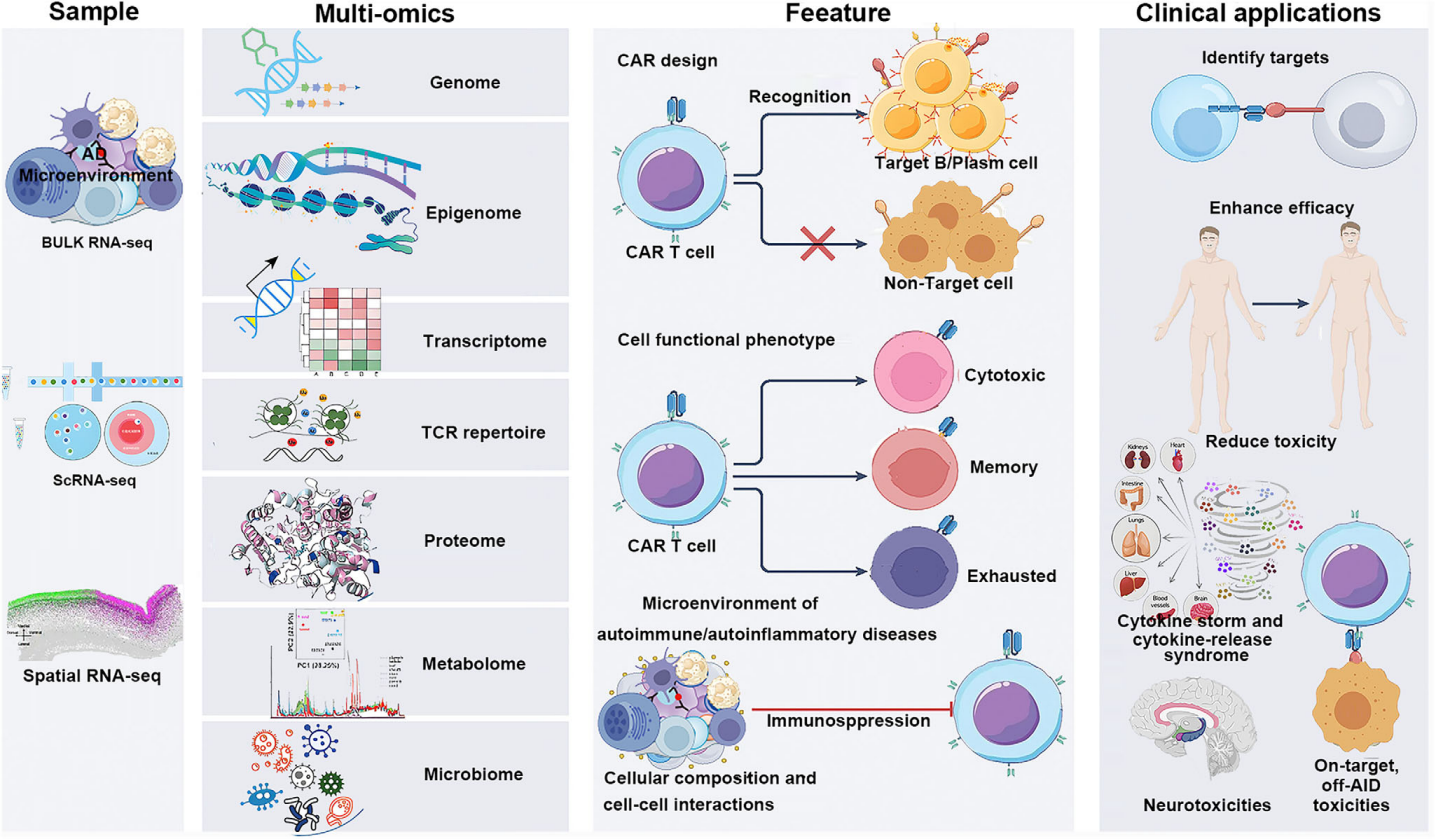
Overview of applications of multiomics data in CAR‐T cell therapy for AIDs. Several types of samples, including bulk tissue specimens, single‐cell suspensions, or spatially preserved tissue slides (left panel), can be used for multidimensional omics analyses, including genomics, epigenomics, transcriptomics, T cell receptor (TCR) repertoire profiling, proteomics, metabolomics, and/or microbiomics (middle left panel), across a multitude of applications in the field of chimeric antigen receptor (CAR) T cell therapy for autoimmune disorders. The valuable multiomics data facilitate the characterization of diverse immune cell features, pathogenic cell populations, cell functional phenotypes (e.g., of CAR‐T cells both prior to and after infusion), and the immunological landscape of target tissues in autoimmune contexts (middle right panel). These insights enable advances in the safety and efficacy of CAR‐T cell therapy through improved target identification, precision cell engineering, and modulation of pathogenic immune circuits (right panel). (This figure was created using BioRender.com.)

To accelerate field‐wide progress, a standardized, open‐access data platform is urgently needed: (1) unified data standards: establish community‐wide guidelines for multiomics data acquisition, processing, and annotation in CAR‐T‐treated AID patients (adhering to FAIR principles); (2) integrated clinical‐omics databases: link electronic health records, imaging, biopsy data, longitudinal follow‐up, and multiomics layers to construct “digital twin patient” models; (3) AI‐driven knowledge discovery: employ federated learning to uncover hidden patterns across institutions while preserving data privacy—facilitating discovery of novel targets, biomarkers, and combination strategies. By harnessing the full power of multidimensional omics, we can transform CAR‐T therapy for AIDs from a promising intervention into a predictable, mechanism‐informed, and precisely tailored modality—ushering in a new paradigm of immune restoration over immune suppression. The investigation of key scientific questions in CAR‐T cell therapy for AIDs—through multidimensional omics technologies, specifically including genomics, epigenomics, transcriptomics, TCR repertoire profiling, proteomics, metabolomics, and microbiomics, analyzed at both bulk and single‐cell levels—is illustrated in Figure 5.

## 6 Prospect of CAR‐T Cell Therapy in AIDs

CAR‐T cell therapy has demonstrated initial application potential in AIDs. Existing technologies such as UCAR‐T cell therapy, mRNA CAR‐T cell therapy, and multitarget CAR‐T cell therapy have shown efficacy in this field and are poised for further optimization in future research endeavors. UCAR‐T cells constructed from T cells obtained from healthy donors play a crucial role in enhancing the accessibility of such therapies. Scalable production and preparation not only reduce treatment costs but also effectively circumvent issues related to suboptimal efficacy of autologous CAR‐T cells due to T cell dysfunction. However, UCAR‐T cell therapy still encounters challenges like graft‐versus‐host reactions and rapid clearance by the host immune system, leading to suboptimal sustained presence in the body. Future improvements can be achieved through stronger pretreatment regimens or gene editing techniques. Unlike the clearer and more easily targeted characteristic molecules found in hematologic malignancies (such as CD19, CD20, BCMA), most self‐antibody‐mediated specific antigen targets in AIDs remain unidentified, making the quest for suitable therapeutic targets a focal point of future research. Based on preliminary evidence of the efficacy of targeting CD38 in SLE, it is speculated that CAR‐T cells targeting both CD19+ B cells and CD38+ plasma cells hold similar application potential [30].

**FIGURE 6:**
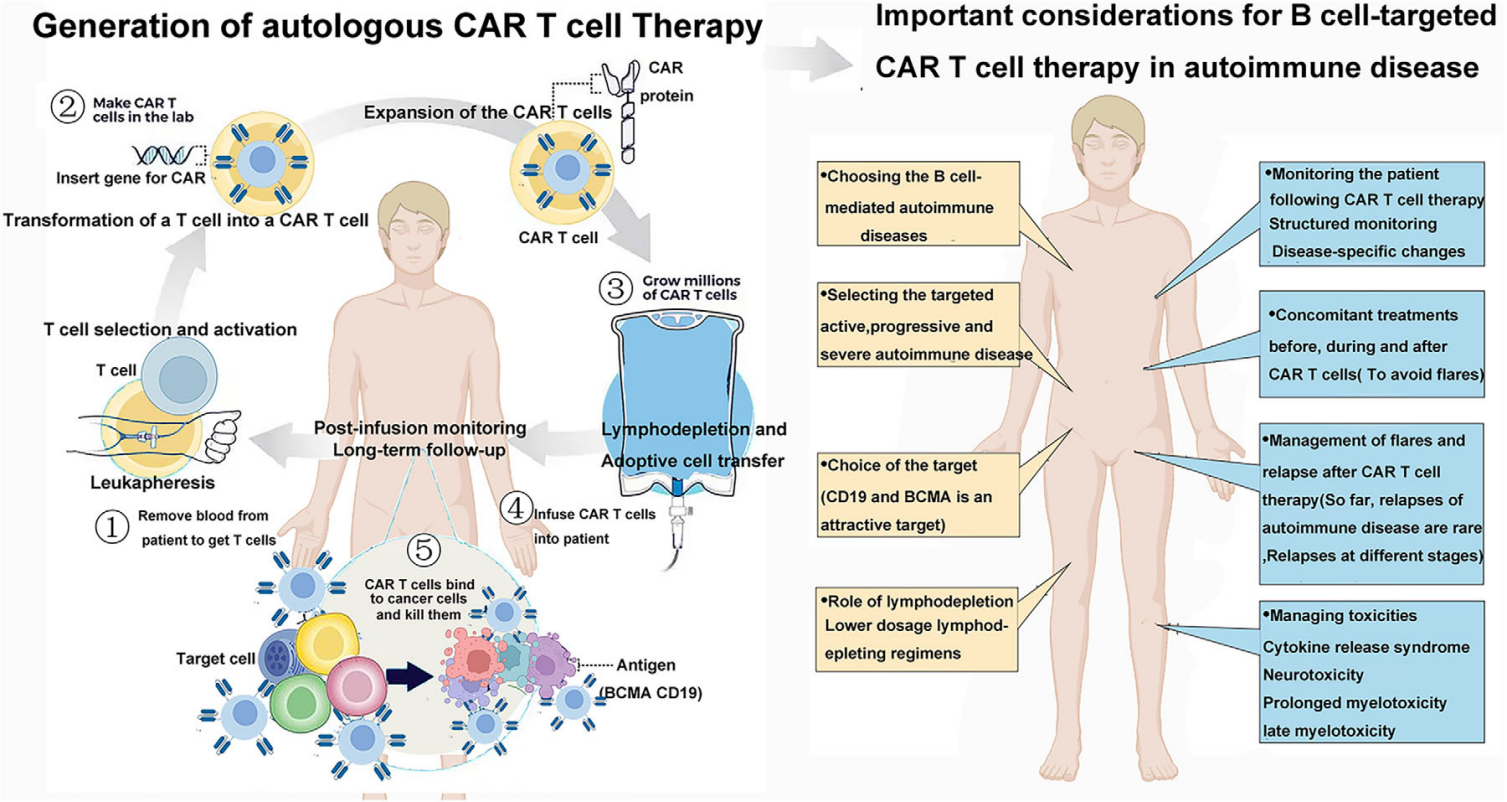
The safety and cost effectiveness of autologous CAR‐T cell therapy in AIDs, with a focus on patient‐specific targets, stages of disease progression, and associated challenges. Profound B‐cell depletion, including the eradication of autoreactive B‐cell clones, is essential for the successful implementation of CD19‐ or BCMA‐targeted CAR‐T cell therapies. Consequently, only those diseases predominantly driven by B cells are likely to respond favorably to CAR‐T cell therapy. Conditions such as systemic lupus erythematosus, idiopathic inflammatory myopathy, and systemic sclerosis are potential candidates for B‐cell‐targeted CAR‐T cell therapy. At present, patients with refractory disease, minimal organ damage, but a high risk of permanent organ failure or mortality, appear to benefit most from CAR‐T cell therapy. Long‐term follow‐up monitoring is crucial for all patients undergoing CAR‐T cell therapy, as infections are among the most common intermediate‐term adverse effects associated with this treatment. Consequently, it is advisable to conduct comprehensive monitoring of immune reconstitution, encompassing T‐cell and NK‐cell subsets, in addition to tracking CAR‐T cell and B‐cell dynamics, to enhance the understanding of immune cell population changes following therapy. Posttreatment surveillance should also encompass evaluations of organ function and immunological parameters, with repeated assessments of reductions in autoantibody levels. Moreover, the prioritization of disease activity biomarkers, composite disease scores, and patient‐reported quality‐of‐life metrics as essential monitoring indicators is recommended. (This figure was created using BioRender.com.)

Moreover, the reduction or dysfunction of Tregs is a potential pathogenic factor in AIDs. Researchers are exploring the incorporation of CAR structures on the surface of Tregs to develop CAR–Tregs. Currently, CAR–Tregs cells have shown promising efficacy in mouse models of IBD, MS, T1D, and RA [50]. Studies on IL23R–CAR Tregs in CD treatment indicate their ability to migrate to IL23R‐expressing tissues and exhibit specific activation in active colonic tissue biopsies [205]. Additionally, transfection with FOXP3 enables Tregs to regain regulatory activity, addressing the issues of low peripheral Treg cell numbers and intrinsic Treg cell functional deficits [206].mRNA CAR‐T cells, due to their shorter in vivo duration of action, can swiftly degrade upon completing intended therapy, reducing long‐term side effects and immunosuppression requirements. However, this transient effect also brings about the challenge of a shorter effective duration. Current investigations are exploring technologies such as sustained‐release mRNA delivery systems, mRNA sequence optimization, or constructing circular RNA to prolong the duration of CAR expression, aiming to enhance efficacy [207, 208] (Figure 6). Recently, an international expert panel convened by the European Society for Blood and Marrow Transplantation and the International Society for Cell & Gene Therapy conducted a comprehensive review of all available evidence regarding the use of CAR‐T cell therapy in patients with AIDs involving rheumatological, neurological, and gastroenterological indications, based on current literature and expert clinical practice [209]. The consensus guidelines emphasize that the decision to proceed with CAR‐T cell therapy in AD patients must be guided by a multifactorial risk–benefit assessment. Key considerations include, first and foremost, disease severity and refractoriness—typically reserved for patients with severe AD who have failed to respond to at least two lines of immunosuppressive therapy. Second, the patient's overall fitness is critical and should be evaluated using performance status criteria (e.g., ECOG <2, Karnofsky score >60%, or Lansky score >60% in pediatric patients) and adequate organ function, particularly renal function (e.g., creatinine clearance >30 mL/min). Active infection constitutes a contraindication to CAR‐T cell therapy, and latent infections (e.g., HIV) may interfere with the manufacturing process of autologous CAR‐T cell products. Additional factors include fertility status, psychological readiness, and the patient's capacity to adhere to complex postinfusion monitoring and management protocols. The panel also highlighted several areas requiring further development: enhancing the safety of next‐generation CAR platforms—including risks associated with CRISPR–Cas9 gene editing and incorporation of suicide switches; advancing the development of allogeneic “off‐the‐shelf” CAR‐T cell products; and optimizing novel CAR designs for improved specificity and durability in autoimmune contexts.

From a clinical management perspective, careful attention must be paid to pretreatment considerations such as washout periods for prior immunomodulatory agents and lymphodepletion regimens. Postinfusion care necessitates structured, multidisciplinary follow‐up to monitor for efficacy, toxicities (e.g., CRS and ICANS), and long‐term immune reconstitution.

In summary, the innovative application of CAR‐T cell therapy in AIDs has overcome many limitations of traditional therapies, achieving sustained medication‐free remission in severe, refractory patients and holding potential for profound immune reconstitution. Nonetheless, further preclinical studies are necessary to provide a solid theoretical foundation for the initiation of multicenter, large‐scale clinical trials. Moreover, there is a need to continue refining the efficacy prediction and treatment safety assessment system for CAR‐T cell therapy to develop more precise, personalized treatment strategies that maximize patient benefits.

## 7 Future Strategies and Innovative Approaches for CAR‐T Cell Therapy in AIDs

This chapter systematically elaborates on the innovative directions and implementation paths to overcome the current bottlenecks of CAR‐T cell therapy in AIDs, focusing on precision optimization, platform iteration, multimodal synergy, and clinical translation, aiming to provide a comprehensive framework for advancing CAR‐T therapy from “deep remission” to “durable cure.” The content is structured in a progressive logic from core technical optimization to integrated application, and finally to translational governance: first, it explores the expansion of therapeutic targets (Section 6.1) and the iteration of next‐generation CAR‐T platforms (universal allogeneic and in vivo CAR‐T, Sections 6.2–6.3), laying the foundation for efficacy enhancement and accessibility improvement; second, it extends to diversified therapeutic strategies, including salvage therapy for relapsed patients (sequential antigen targeting, Section 6.4), non‐CAR‐T cellular therapy development and combination (Section 6.5), smart multitarget CAR design (Section 6.6), and multidimensional combination regimens (Section 6.7), to address the heterogeneity and complexity of AID pathogenesis; finally, it focuses on regulatory and translational challenges (Section 6.8), covering endpoint design, safety supervision, ethical considerations, and regulatory system reconstruction, to bridge the gap from scientific breakthrough to clinical application. Collectively, this chapter integrates cutting‐edge technologies, clinical needs, and regulatory practice, depicting a multifaceted, intelligent, and synergistic future landscape of CAR‐T therapy in AIDs.

### 7.1 Future Targets for CAR‐T Cell Therapy in AIDs

Currently, CD19 remains the most extensively studied and clinically applied target in AIDs, owing to its high specificity for the B‐cell lineage and broad expression across the entire B‐cell differentiation pathway—from early pro‐B cells to mature B cells. CD19‐directed CAR‐T cells effectively deplete CD19‐expressing B cells and have demonstrated remarkable efficacy in multiple B‐cell‐driven, refractory AIDs—including SLE and ASS—enabling patients to achieve sustained, drug‐free remission. However, B cells eventually reconstitute following CAR‐T cell contraction. Although this immune reconstitution differs from that observed in oncology and is associated with relatively low relapse rates, it nonetheless suggests the need for more durable control or strategies targeting distinct cellular subsets.

Targets specific to plasma cells, such as BCMA and CD38, offer complementary profiles to CD19. BCMA is highly expressed on LLPCs; BCMA‐directed CAR‐T cells efficiently eliminate plasma cells within lymphoid tissues and rapidly reduce autoantibody titers. Clinical studies indicate that BCMA‐ or BCMA/CD19 dual‐targeted CAR‐T cells induce swift autoantibody decline, albeit frequently accompanied by hypogammaglobulinemia—though in most patients, IgG concentrations recover within 6–12 months. Thus, BCMA‐ and CD38‐targeted strategies may be particularly suited for disease stages or subtypes requiring deep plasma cell depletion to eradicate pathogenic autoantibodies. CD20 and CD22, as other key B‐cell surface antigens, represent highly promising alternative targets. CD20 is expressed during specific stages of B‐cell development and has been successfully exploited in monoclonal antibody therapies for B‐cell lymphomas. CD22 is broadly expressed on B cells and represents a critical strategy to overcome CD19‐negative escape. Dual‐targeted CAR‐T cells (e.g., CD19/CD20, CD19/CD22) have already demonstrated enhanced efficacy and reduced antigen escape in hematologic malignancies—a strategy with equally broad potential in AIDs, offering more comprehensive and durable B‐cell depletion. Future target discovery should not be confined to the B‐cell lineage. CAR‐T therapies targeting nonimmune cells, such as FAP, are already under exploration, opening new avenues for treating AIDs involving tissue remodeling and fibrosis (e.g., SSc). Furthermore, the development of targets specific to autoreactive T‐cell clones represents the next frontier, aiming to directly eliminate the core drivers of disease pathogenesis.

### 7.2 Next‐Generation CAR‐T Platforms—The Future of Allogeneic (Universal) CAR‐T Therapy in AIDs

UCAR‐T therapy—derived from healthy donors, genetically edited (e.g., CRISPR–Cas9‐mediated TCR knockout to mitigate GvHD risk), engineered with CAR constructs, and manufactured as “off‐the‐shelf” products for multiple patients—offers compelling advantages: lower cost, improved accessibility, and elimination of the prolonged wait for autologous cell manufacturing. Globally, multiple off‐the‐shelf UCAR‐T therapies for severe, refractory AIDs have entered clinical trials, demonstrating high safety and efficacy. Studies show that CD19‐targeted UCAR‐T cells can robustly expand in vivo, completely deplete B lymphocytes, and enable B‐cell reconstitution by 3 months postinfusion. However, the primary challenges facing UCAR‐T include host immune rejection of allogeneic cells and residual risk of GvHD. Although initial clinical data indicate favorable safety profiles in AIDs—with lower incidences of CRS and NT compared with oncology applications—long‐term safety and durability require validation in large‐scale trials. Future strategies must focus on optimizing gene‐editing technologies to fully eliminate immunogenicity, developing safer “safety switch” systems for precise control of CAR‐T activity, and exploring reduced‐intensity conditioning regimens to improve patient tolerability. UCAR‐T holds the potential to become a standardized, scalable solution for AID therapy.

### 7.3 Next‐Generation CAR‐T Platforms—The Future Strategy and Prospects of in Vivo CAR‐T Therapy for AIDs

In vivo CAR‐T therapy represents a paradigm‐shifting direction for next‐generation cell therapy. This approach bypasses the complex ex vivo process of cell extraction, genetic modification, expansion, and reinfusion. Instead, it employs “smart delivery vehicles”—such as viral vectors or nanoparticles (e.g., LNPs)—to directly deliver mRNA or DNA encoding the CAR construct into the patient's own T cells in situ, thereby reprogramming them into functional CAR‐T cells within the body. This “one‐shot reprogramming” strategy dramatically simplifies the therapeutic workflow, reduces costs, and minimizes manufacturing complexity.

Capstan Therapeutics’ CD19‐targeted in vivo CAR‐T therapy (CPTX2309) has entered Phase I clinical trials for B‐cell‐mediated AIDs [210]. Pioneering research from the First Affiliated Hospital of University of Science and Technology of China has established, for the first time, the clinical feasibility of LNP‐targeted in vivo CAR‐T therapy for SLE, setting an international benchmark in the field. In vivo CAR‐T holds promise for rapid immune reset and may reduce off‐target effects through precision delivery. Future research must address key challenges: improving targeting efficiency of delivery systems, ensuring controllable and durable CAR expression, and mitigating potential immunogenicity. With continuous advances in nanotechnology and gene delivery tools, in vivo CAR‐T is poised to emerge as a mainstream, convenient therapeutic modality for AIDs (Table 2).

**TABLE 2:** Core technical and translational characteristics of different CAR‐T platform technologies for autoimmune diseases.

Characteristic	Conventional autologous CAR‐T	Universal allogeneic CAR‐T	In vivo CAR‐T
Manufacturing process	Complex ex vivo expansion	Scalable ex vivo production	Direct in vivo generation
Production time	2–3 weeks	Premanufactured	Nearly immediate
Cost considerations	High	Potentially 90% reduction	Expected significant reduction
Lymphodepletion required	Yes	Variable	No
Persistence	Weeks to months	May be limited by host immunity	Transient (days to weeks)
GvHD risk	None	Requires careful editing	None
Redosing potential	Limited	High	High
Current clinical data	Extensive	Emerging	Preliminary human data
References	[211]	[212]	[213]

### 7.4 Salvage Therapy With Sequential CAR‐T Targeting Alternative Antigens in Patients Relapsing After Initial CAR‐T Failure

Despite the remarkable efficacy of CAR‐T therapy in AIDs, a subset of patients may experience relapse. Potential mechanisms include target antigen loss (e.g., CD19‐negative escape) or suboptimal CAR‐T cell expansion and persistence in vivo. For these patients, sequential CAR‐T therapy targeting an alternative antigen represents a highly promising salvage strategy. Clinical successes have already been documented: the team led by Jun Shi at the Institute of Hematology & Blood Diseases Hospital, Chinese Academy of Medical Sciences, reported the world's first successful cases of two patients with multiply relapsed, treatment‐refractory autoimmune hemolytic anemia who achieved remission following salvage therapy with a BCMA‐targeted bispecific T‐cell engager after failing autologous CD19 CAR‐T therapy [214]. This demonstrates the feasibility of sequential targeting across distinct B‐cell or plasma cell subsets. For instance, if initial CD19‐directed therapy fails to eliminate LLPCs—leading to relapse—subsequent administration of BCMA‐ or CD38‐targeted CAR‐T cells may effectively eradicate residual pathogenic plasma cells. Similarly, for patients relapsing with CD19‐negative disease, switching to CD20‐ or CD22‐directed CAR‐T offers a viable alternative. This “target relay” strategy leverages the differential antigen expression profiles across B‐cell developmental stages, providing renewed therapeutic opportunities for relapsed patients. Future efforts must establish comprehensive systems for relapse monitoring and rational target selection to guide individualized sequential therapy.

### 7.5 Future Strategies for AID Therapy Using Non‐CAR‐T Cell Types: CAR‐NK, CAR–Treg, CAR‐γδT, and CAR‐M—Alone or in Combination

**FIGURE 7:**
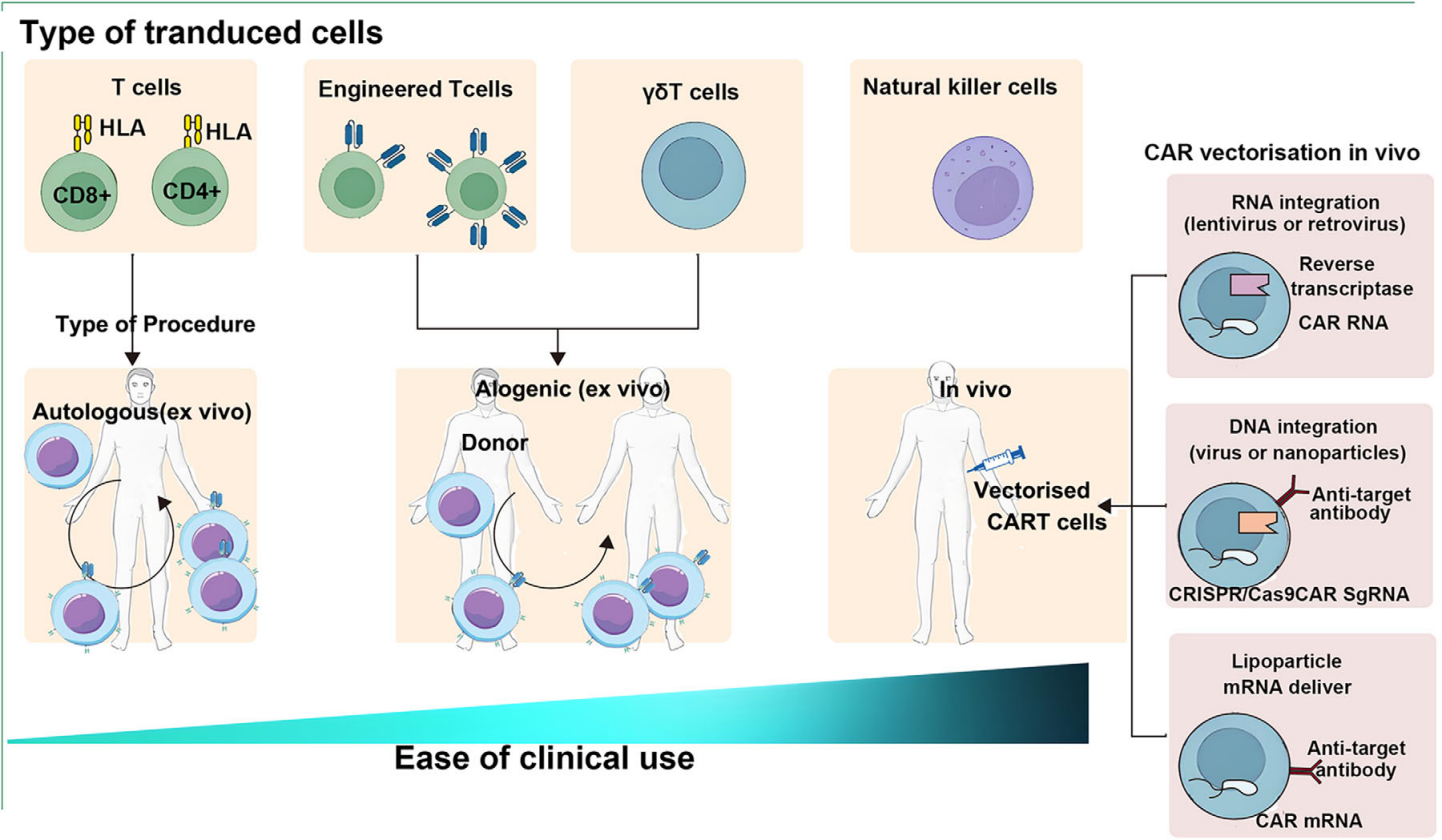
Current and future strategies for CAR use in humans. This schematic illustrates the diverse cellular sources, procedural approaches, and vectorization methods used in chimeric antigen receptor (CAR) therapy, highlighting the progression from current ex vivo strategies to emerging in vivo approaches. On the left, different types of transduced cells are shown, including conventional T cells (CD8+ and CD4+), engineered T cells (e.g., with enhanced signaling domains), γδ T cells, and natural killer (NK) cells, each offering distinct immunological properties. The central panel depicts two main procedural modalities: autologous (ex vivo) therapy, where patient‐derived cells are genetically modified and reinfused, and allogeneic (ex vivo) approaches using donor cells, which may enable off‐the‐shelf therapies. On the right, recent advances in in vivo CAR delivery are summarized, including RNA‐based strategies (via lentivirus/retrovirus or reverse transcriptase‐mediated CAR RNA expression), DNA integration using viral vectors or nanoparticles (with optional antitarget antibodies to enhance specificity), CRISPR/Cas9‐mediated CAR gene editing guided by sgRNA, and lipoparticle‐mediated mRNA delivery. The gradient bar at the bottom reflects the relative ease of clinical implementation, with current ex vivo methods being more established but complex, while in vivo approaches offer greater scalability and accessibility but remain under active investigation. These evolving technologies aim to improve safety, efficacy, and broad applicability of CAR‐based therapies in autoimmune and inflammatory diseases, including rheumatoid arthritis and other rheumatic conditions. *Abbreviations*: HLA = human leukocyte antigen; sgRNA = single guide RNA; CAR = chimeric antigen receptor. (This figure was created using BioRender.com.)

The future landscape of cellular therapy will extend beyond conventional αβ T cells to encompass diverse immune cell types, aiming for safer, more precise, and more comprehensive immunomodulation. CAR–Tregs: Tregs are central to maintaining immune tolerance. Engineering CAR–Tregs enables them to home to specific tissues or recognize disease‐relevant antigens (e.g., myelin proteins, HLA molecules), thereby locally restoring immune homeostasis. Preclinical studies have demonstrated that CAR–Tregs effectively suppress autoimmune responses—and even reverse disease—in animal models of MS, T1D, and colitis. Their key advantage lies in immunosuppression without cytotoxicity, theoretically offering superior safety, particularly for chronic diseases requiring long‐term immune regulation. CAR‐NK cells: NK cells exhibit MHC‐unrestricted cytotoxicity, lower risk of CRS, and potential for allogeneic (“off‐the‐shelf”) use. CAR‐NK cells have already shown therapeutic potential in SLE patients with heterogeneous manifestations, including LN. Their “off‐the‐shelf” nature and favorable safety profile position them as powerful complements—or alternatives—to UCAR‐T therapies. CAR‐γδT cells: γδ T cells bridge innate and adaptive immunity and can recognize non‐MHC‐restricted antigens. Although research in AIDs remains limited, their unique recognition mechanisms and strong tissue‐homing capacity suggest potential advantages in targeting specific inflammatory niches. CAR‐M (macrophages): macrophages are potent phagocytes and APCs. While primarily explored in solid tumors, their robust phagocytic function and ability to remodel microenvironments—for example, repolarizing proinflammatory M2 macrophages toward anti‐inflammatory M1 phenotypes—hold unique promise for AIDs characterized by immune complex deposition or chronic inflammation, such as RA and LN. Future strategies will likely transcend single‐cell‐type therapies, instead exploring rational combinations. For example: CAR‐T cells could be used first for deep depletion of pathogenic B cells, followed by CAR–Treg infusion to promote tolerance re‐establishment; or CAR‐NK cells could be coadministered to provide broad immune surveillance. Such multicellular, synergistic approaches may enable more complete and durable disease control (Figure 7).

### 7.6 Future Strategies for Multitarget Next‐Generation CAR‐T Cells in AIDs

Next‐generation CAR‐T cell design is evolving toward greater intelligence, safety, and efficacy. Multitargeting represents a core strategy to overcome limitations of single‐antigen targeting, such as antigen escape and incomplete cell depletion. (1) Tandem CAR (TanCAR) or dual‐target CAR: these constructs simultaneously recognize two antigens (e.g., CD19 and CD20), activating T cells only when both antigens are present (AND‐gate logic) or when either is present (OR‐gate logic). This not only enables more comprehensive B‐cell lineage clearance but also enhances specificity, minimizing “off‐target” damage to healthy tissues. For instance, dual‐target CAR‐T products for SLE have already entered clinical development. (2) Logic‐gated CARs: advanced logic‐gated systems (e.g., AND‐NOT gates) allow CAR‐T cells to recognize target antigens while avoiding cells expressing specific “safety” antigens—enabling unprecedented precision. Although primarily explored in solid tumors, this approach holds immense potential in AIDs to distinguish pathogenic immune cells from healthy counterparts, dramatically widening the therapeutic safety window. (3) Armored CAR‐T cells: next‐generation CAR‐T cells may be further engineered to express immunomodulatory molecules—such as IL‐10 or TGF‐β—to promote local tolerance, or to incorporate “safety switches” (e.g., inducible caspase9) for rapid elimination in case of severe toxicity.

### 7.7 Combination Strategies and Adjunctive Approaches: Multidimensional Coordinated Intervention in Autoimmune Pathogenesis

Although CAR‐T cell therapy has demonstrated revolutionary efficacy in depleting pathogenic B cells and inducing immune reconstitution, the pathogenesis of AIDs is highly heterogeneous and multifactorial, involving diverse immune cell subsets (e.g., Tfh, Th17, plasma cells, macrophages), cytokine networks (e.g., IL‐6, BAFF, IFN‐I, IL‐17, IL‐23), defects in immune tolerance, epigenetic dysregulation, and tissue microenvironment remodeling. While B‐cell‐targeted CAR‐T therapy can induce deep remission, it may not eradicate all pathogenic circuits—particularly in T‐cell‐driven or innate‐immune‐mediated diseases (e.g., certain subtypes of RA, psoriasis, T1D). Thus, the future therapeutic paradigm must shift from “monotherapy” to “systems synergy”—employing rationally designed combination strategies and adjunctive therapies to coordinately intervene across multiple dimensions of autoimmune pathogenesis, thereby achieving more durable and comprehensive immune homeostasis. For example, combining cytokine modulation with CAR‐T therapy may actively shape a favorable immune microenvironment during the “immune gap” or “immune reconstitution” phase post‐CAR‐T: (1) low‐dose IL‐2 therapy to selectively expand Tregs; (2) anti‐IL‐6R or anti‐IL‐6 monoclonal antibodies to suppress inflammatory storms and plasma cell survival; (3) BAFF/BLyS inhibitors to modulate the “quality” rather than the “quantity” of B‐cell reconstitution; (4) CAR‐T combined with antigen‐specific peptide/MHC multimers or nanoparticle‐based vaccines to re‐educate the immune system; (5) CAR‐T combined with adoptive Treg transfer or CAR–Treg therapy to reinforce tolerance.

In summary, biomarker‐guided integration will enable truly personalized approaches based on individual disease features, B‐cell subset dynamics, and CAR‐T PK parameters. Future AID management will no longer rely on “one‐size‐fits‐all” CAR‐T monotherapy, but will dynamically tailor “CAR‐T + X” combinatorial regimens based on patient‐specific disease subtypes, immune phenotypes, biomarker profiles (e.g., autoantibody repertoire, cytokine signatures, TCR/BCR clonality), and genomic features. Examples include: (1) for SLE patients with high IFN‐I signatures: CAR‐T + JAK inhibitor (e.g., baricitinib) or anti‐IFNAR monoclonal antibody; (2) for psoriatic arthritis dominated by the Th17/IL‐23 axis: CAR‐T + anti‐IL‐23p19 (e.g., guselkumab); (3) for patients harboring pathogenic T‐cell clones: CAR‐T + TCR‐based vaccines or TCR‐T cell clearance therapy; (4) for patients with severe gut dysbiosis: CAR‐T + customized fecal microbiota transplantation (FMT) + gut barrier restoratives (e.g., glutamine). This multidimensional, dynamic combinatorial strategy will propel AID therapy beyond “disease control” toward “immune cure,” ultimately realizing the vision of “Precision Immunology”—delivering the right immune intervention, with the right combination, to the right patient, at the right time.

### 7.8 Regulatory Considerations and Clinical Translation of Next‐Generation CAR‐T Cells: Systemic Challenges and Pathway Restructuring From Scientific Breakthrough to Patient Bedside

The rapid advancement of CAR‐T cell therapy in AIDs has transitioned from proof‐of‐concept to accelerated clinical translation. With diversification of targets (CD19, BCMA, CD20, FAP, etc.), cell sources (autologous, allogeneic “off‐the‐shelf,” in vivo generated), structural sophistication (dual‐targeting, logic‐gated circuits, controllable switches), and increasingly complex combination strategies (with cytokines, tolerance induction, microbiome modulation), the complexity of next‐generation CAR‐T products is growing exponentially. This scientific leap poses unprecedented challenges to existing regulatory frameworks, clinical trial designs, manufacturing and quality control systems, safety assessment standards, and health economic models. To bridge the gap from “laboratory breakthrough” to “clinical routine,” a forward‐looking, flexible, and patient‐centered regulatory and translational ecosystem tailored to the unique features of AIDs must be constructed.

Current AID clinical trials predominantly rely on DASs (e.g., SLEDAI, DAS28), autoantibody titers, and corticosteroid dosage as primary endpoints. However, the goal of CAR‐T therapy is “immune reset” and “sustained drug‐free remission,” necessitating efficacy assessments that transcend short‐term symptom control and instead focus on fundamental immunological reconstitution. (1) Core endpoint: “duration of drug‐free remission” (DFRD)—defined as the time during which patients maintain clinical remission (e.g., SLEDAI ≤4) and immunological stability (e.g., negative anti‐dsDNA, normalized complement levels) after complete discontinuation of all ISs. DFRD should serve as the primary endpoint—not a surrogate—in registrational trials. (2) Incorporation of “immune reconstitution profiles” as key biomarkers—including B‐cell/T‐cell subset reconstitution kinetics, TCR/BCR repertoire diversity restoration, Treg/Th17 balance, and clearance rate of autoreactive clones. These metrics can predict long‐term remission or relapse risk and guide personalized interventions. (3) Establishment of “functional cure” criteria—drawing inspiration from the oncology concept of “minimal residual disease,” develop an “immune residual disease” detection system for AIDs, such as high‐throughput sequencing to track the persistence of pathogenic BCR/TCR clones. Concurrently, regulatory agencies (e.g., US FDA, EMA, NMPA) must collaborate with academia and industry to establish AID‐specific guidelines for CAR‐T efficacy evaluation, avoiding the uncritical adoption of endpoints designed for oncology or conventional ISs.

Moreover, the safety profile of CAR‐T in AIDs differs fundamentally from that in oncology: patients typically lack tumor burden, have not undergone deep immunosuppression via chemotherapy, and receive milder preconditioning regimens (e.g., low‐dose CTX ± fludarabine), resulting in significantly lower incidences of CRS and ICANS. However, novel mechanisms introduced by next‐generation CAR‐T platforms—such as in vivo generation, allogeneic formats, and multitargeting—bring new safety considerations. Regulatory agencies should spearhead the establishment of a global “CAR‐T in AIDs safety registry” to enable data sharing and early signal detection of emerging risks. Traditional oncology CAR‐T trials often employ single‐arm, open‐label designs due to the dire prognosis and lack of effective alternatives. In contrast, AID patients typically have multiple therapeutic options (e.g., biologics, JAK inhibitors) and a relatively indolent disease course, necessitating more rigorous controlled trial designs. Regulatory bodies should issue “guidelines for clinical trial design of CAR‐T cell therapy in AIDs,” clearly specifying design requirements for different product types (autologous/allogeneic/in vivo) and disease stages (treatment‐naïve/refractory).

Currently, CAR‐T in AIDs is mostly reserved for “end‐stage refractory patients,” yet its potential for cure suggests earlier intervention may be warranted. This raises critical ethical questions: (1) timing of intervention: should CAR‐T be administered early in disease (e.g., within 1–2 years of diagnosis) to prevent irreversible organ damage? This requires balancing the potential curative benefit against long‐term unknown risks (e.g., fertility impairment, secondary malignancies). (2) Complexity of informed consent: patients must understand the meaning of “immune reset,” consequences of B‐cell depletion (e.g., loss of vaccine immunity), necessity of lifelong follow‐up, and potential risk of de novo autoimmunity. Interactive, visualized consent tools must be developed. (3) Special considerations for children and reproductive‐age populations: data on CAR‐T's impact on fertility and fetal development remain scarce. Dedicated registries and prospective studies are urgently needed. In vivo CAR‐T, lacking ex vivo manipulation, may be particularly suitable for pediatric populations. Over the next 5–10 years, regulatory science must evolve in parallel with technological innovation. We call for: (1) establishment of an “international regulatory consortium for advanced Therapies in AIDs” (e.g., joint US FDA–EMA–NMPA–PMDA task force) to harmonize technical standards and review pathways; (2) development of a “risk‐tiered regulatory framework for AID CAR‐T products,” implementing graded oversight based on risk profiles (e.g., autologous vs. in vivo, single‐target vs. logic‐gated); (3) investment in “foundational regulatory science research,” including organoid models to predict long‐term CAR‐T safety, AI‐driven toxicity prediction algorithms, and standardized platforms for immune reconstitution assessment.

Only through deep collaboration among science, regulation, industry, and patient communities can the revolutionary potential of CAR‐T therapy in AIDs be translated into safe, effective, accessible, and affordable clinical reality—ultimately fulfilling the medical dream of “one‐time treatment, lifelong remission.”

In summary, the future strategy for CAR‐T cell therapy in AIDs is multifaceted, intelligent, and synergistic. Through precision targeting, off‐the‐shelf manufacturing, in vivo reprogramming, sequential targeting, multicell‐type combinations, and smart multitarget designs, we aim to transform today's “deep remissions” into “durable cures,” offering revolutionary therapeutic options for millions of patients worldwide. The rapid progress in this field marks our arrival at the threshold of a new era—one in which the immune system can be safely and effectively “reprogrammed” to restore its fundamental capacity for self‐tolerance.

## Author Contributions

Zhidan Fan and Haiguo Yu contributed to the research design. Zhidan Fan and Li Zhang contributed to the data management and wrote the manuscript. All authors have read and approved the published version of the manuscript.

## Funding

This study was supported by the China Postdoctoral Science Foundation (Certificate Number: 2024M751498).

## Ethics Statement

The authors have nothing to report.

## Conflicts of Interest

The authors declare no conflicts of interest.

## Supporting information



Supporting File 1: mco270658‐sup‐0001‐TableS1.xlsx

## Data Availability

The authors have nothing to report.
